# Structural mechanism of extranucleosomal DNA readout by the INO80 complex

**DOI:** 10.1126/sciadv.add3189

**Published:** 2022-12-09

**Authors:** Franziska Kunert, Felix J. Metzner, James Jung, Markus Höpfler, Stephan Woike, Kevin Schall, Dirk Kostrewa, Manuela Moldt, Jia-Xuan Chen, Susanne Bantele, Boris Pfander, Sebastian Eustermann, Karl-Peter Hopfner

**Affiliations:** ^1^ Gene Center, Department of Biochemistry, Ludwig-Maximilians-Universität München, Munich, Germany.; ^2^ DNA Replication and Genome Integrity, Max Planck Institute of Biochemistry, Martinsried, Germany.; ^3^ Institute of Molecular Biology (IMB), Mainz, Germany.; ^4^ European Molecular Biology Laboratory (EMBL), Heidelberg, Germany.

## Abstract

The nucleosomal landscape of chromatin depends on the concerted action of chromatin remodelers. The INO80 remodeler specifically places nucleosomes at the boundary of gene regulatory elements, which is proposed to be the result of an ATP-dependent nucleosome sliding activity that is regulated by extranucleosomal DNA features. Here, we use cryo–electron microscopy and functional assays to reveal how INO80 binds and is regulated by extranucleosomal DNA. Structures of the regulatory A-module bound to DNA clarify the mechanism of linker DNA binding. The A-module is connected to the motor unit via an HSA/post-HSA lever element to chemomechanically couple the motor and linker DNA sensing. Two notable sites of curved DNA recognition by coordinated action of the four actin/actin-related proteins and the motor suggest how sliding by INO80 can be regulated by extranucleosomal DNA features. Last, the structures clarify the recruitment of YY1/Ies4 subunits and reveal deep architectural similarities between the regulatory modules of INO80 and SWI/SNF complexes.

## Teaser

Cryo-EM and functional analyses reveal how the nuclear actin module senses linker DNA to regulate nucleosome remodeling by INO80.

## INTRODUCTION

Chromosomal DNA is predominantly organized in the form of nucleosome core particles (NCPs)—~147 base pairs (bp) of DNA wrapped around the histone octamer (two copies of histones 2A, 2B, 3, and 4)—along with interspersed extranucleosomal linker DNA as well as larger nucleosome-free regions (NFRs) or nucleosome-depleted regions (NDRs) (*1*). NFRs and NDRs are important regulatory regions and are found at promoters, enhancers, and origins of replication in *Saccharomyces cerevisiae* (*2*). Nucleosomal packaging not only condenses and protects DNA but also generates epigenetic information in the form of nucleosome occupation, histone modifications, and histone variant composition (*2*).

The location, composition, and epigenetic modifications of nucleosomes play key roles in the regulation of gene expression, DNA replication, and DNA repair and are shaped by the collective action of chromatin remodelers and epigenetic modifiers. Chromatin remodelers are molecular machines that use the energy of adenosine triphosphate (ATP) hydrolysis to slide, position, evict, or edit nucleosomes (*3*, *4*). They are generally grouped into four main families: INO80/SWR1, SWI/SNF, ISWI, and CHD. Common to all remodelers is a Swi2/Snf2-type adenosine triphosphatase (ATPase) domain that uses ATP hydrolysis to translocate DNA. This basal activity is converted into the diverse remodeling reactions by additional remodeler-specific domains or subunits (*5*).

INO80 is a >1-megadalton chromatin remodeler that is conserved from yeast to human (*6*, *7*) and emerges as a central multisubunit enzyme complex that determines chromatin structure around NDRs/NFRs (*8*). INO80 slides canonical nucleosomes and hexasomes (i.e., nucleosomes lacking one H2A-H2B dimer), forms regularly spaced nucleosomal arrays, and exchanges histone variants in vitro (*9*–*11*). Hereby, INO80 shows a uniquely robust ability to position +1 (i.e., transcription start site) and −1 (opposite side) nucleosomes that generate the boundary to the nucleosome-free DNA in NDRs/NFRs in genome-wide in vitro chromatin reconstitution assays (*12*). In vivo, INO80 is implicated in NDR/NFR and array formation as well (*13*, *14*).

A comprehensive mechanistic framework for the different biochemical activities of INO80 and how they are regulated or work together is still largely elusive. For instance, the detailed structural mechanism by which INO80 determines +1 and −1 nucleosome positions remains unclear. Not only NFR located barrier factors such as *S. cerevisiae* Reb1 and DNA ends but also NFR features such as promoter DNA mechanics and shape recently emerged as regulators of INO80-mediated nucleosome positioning in whole-genome chromatin reconstitutions (*15*–*17*). In mammals, INO80 might be regulated, in part, by the DNA sequence because the DNA binding transcription factor YY1 (Yin Yang 1), an early developmental regulator and structuring factor of promoter-enhancer elements, is a component of the human INO80 complex. Together, current evidence suggests that INO80 acts as an information processing hub that integrates diverse sources of information to properly shape chromatin around gene promoter regions (*15*, *16*).

Structural studies on INO80 and other remodelers revealed basic principles of how these molecular machines (or subcomplexes) bind nucleosomes and mobilize nucleosomal DNA using cycles of ATP binding and hydrolysis (*18*–*25*). Even in light of this process, we are far from understanding how complex remodeling reactions are carried out in a highly regulated manner, owed in part to their complex, dynamic, and modular architecture. INO80 contains more than 15 subunits, organized in three structural modules that we denote “N,” “A,” and “C.” Up to now, structural information is available for the C-module bound to the nucleosome, as well as parts of the A-module in the absence of DNA. The Ino80 polypeptide itself carries the core ATPase motor activity and acts as a scaffold for the three modules. The C-module is the core nucleosome sliding unit: It contains the Swi2/Snf2 ATPase motor domain of Ino80p (Ino80^motor^), the scaffolding AAA^+^ ATPases Rvb1 and Rvb2, and nucleosome binding subunits Ies2 (Ino eighty subunit 2), Ies6, and Arp5 (actin-related protein 5) (*20*, *21*). The NCP is bound by Ino80^motor^-Ies2 at DNA superhelical location SHL-6 and by Arp5-Ies6 at DNA SHL-2. Furthermore, the Arp5 “grappler” insertion domain interacts with the nucleosome “acidic patch,” a motif at the H2A/H2B interface that is a binding site for numerous chromatin proteins (*20*). In this configuration, Ino80^motor^ pumps extranucleosomal entry DNA into the NCP, a model that can explain its sliding activity (*10*, *20*, *22*). The function of N- and A-modules is less clear. The N-module is evolutionarily rather divergent, binds DNA, and has autoregulatory functions to ensure switch-like activation of INO80 by extranucleosomal DNA (*26*). The A-module is highly conserved in evolution and contains an HSA (helicase-SANT–associated) domain (Ino80^HSA^) in the middle of the Ino80p polypeptide chain, along with actin (Act1), Arp4, Arp8, Ies4, and Taf14. The complex of Ino80^HSA^ with Arp4, actin, and Arp8 has been crystallized, and low-resolution structural along with functional analysis suggests that the Ino80^HSA^ domain acts as an extranucleosomal DNA sensor, which is required for robust nucleosome sliding (*27*, *28*) and positioning in whole-genome chromatin reconstitution (*15*, *16*).

It is yet unclear how the A-module binds DNA and how it regulates the C-module. A-modules are found in all multisubunit remodelers of the INO80/SWR1 family and carry nuclear actin, and while their functional importance is well established, the underlying regulatory and sensing mechanisms are unclear. Here, we present cryo–electron microscopy (cryo-EM) structures of the regulatory INO80 A-module (*Chaetomium thermophilum, S. cerevisiae,* and *Homo sapiens*), the A-module bound to DNA (*C. thermophilum, S. cerevisiae*), and an overall structure of the INO80 A- and C-modules in an extranucleosomal DNA sensing configuration (*C. thermophilum*). Supported by yeast in vivo studies, the structures reveal the mode of extranucleosomal DNA binding and identify both Ino80^HSA^ and Arp8 as core DNA binding elements. DNA can bind along the A-module in a notably curved fashion, which, together with biochemical analysis, supports a function as a DNA feature sensor. The overall structure of the A-module and C-module–nucleosome complex, along with high-resolution views of the motor domain in nucleotide-free (*apo*) and adenosine diphosphate (ADP)∙BeF*_x_* states, suggests how extranucleosomal DNA sensing and DNA mechanical features might regulate INO80 through an allosteric link to the motor domain. Last, we reveal that yeast/fungal Ies4 and human YY1 are structural homologs. A double tryptophan (2W)–anchored hairpin of Ies4/YY1 emerges as an evolutionarily conserved Arp4-actin anchor motif that unifies core A-module compositions across INO80 and SWI/SNF-type remodelers and provides links to polycomb repressive complexes. Together, our data provide a structural framework for regulation of INO80 by extranucleosomal DNA.

## RESULTS

### Architecture of the INO80 regulatory A-module

**Fig. 1.**
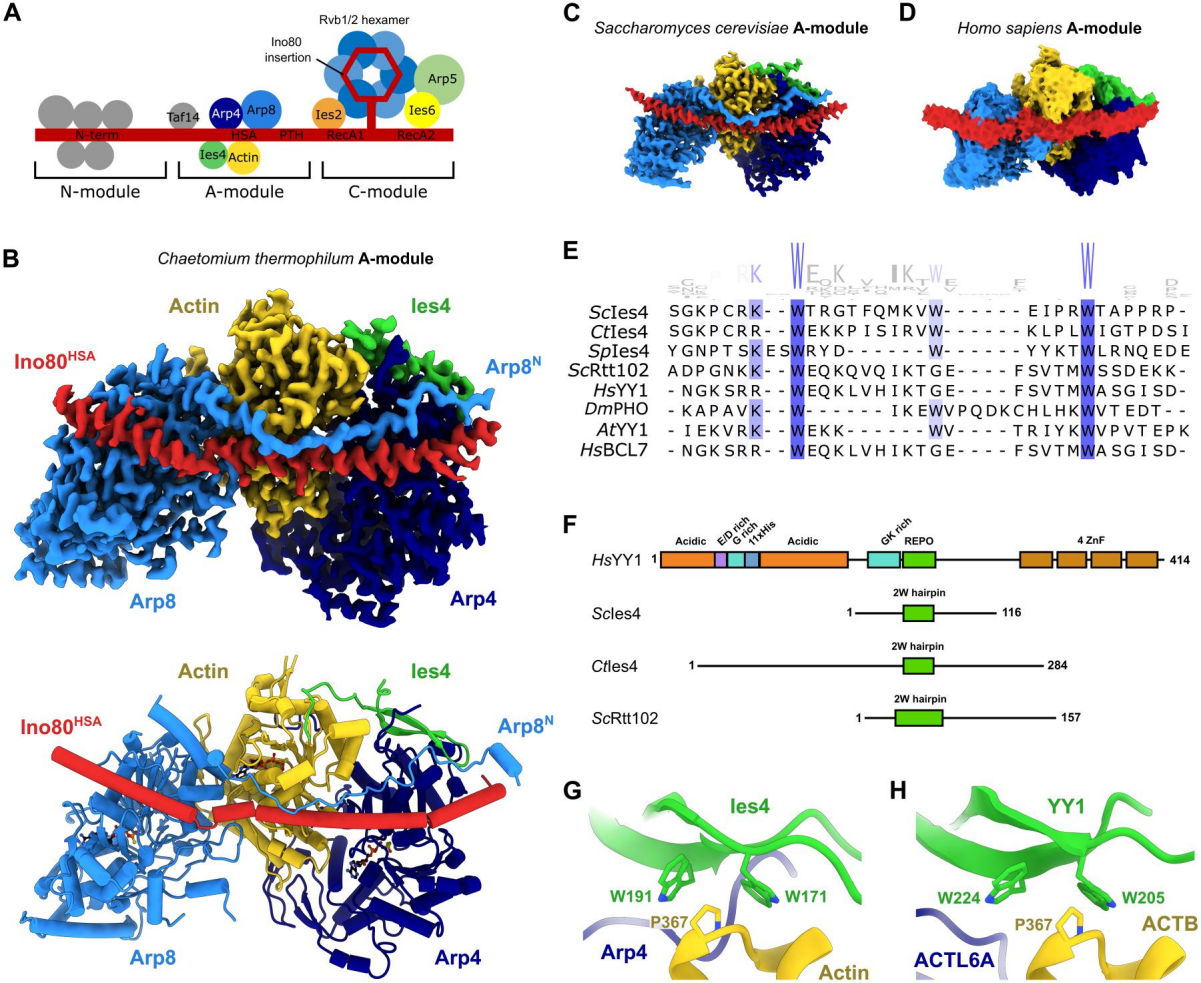
Structure of the INO80 A-module. (**A**) Schematic of INO80 complex submodule and subunit organization. (**B**) Cryo-EM reconstruction (top) and structural model (bottom) of *C. thermophilum* (*Ct*) A-module. The protein subunits are color-coded and annotated. (**C**) Cryo-EM reconstructions of *S. cerevisiae* (*Sc*) and (**D**) cryo-EM reconstitution of *H. sapiens* (*Hs*) A-modules color-coded as in (B). (**E**) Multiple sequence alignment (*75*) of the REPO/2W-motif of *S. cerevisiae* Ies4 and related actin/Arp-interacting proteins. *Sp*, *Schizosaccharomyces pombe*; *Dm*, *Drosophila melanogaster*; *At*, *Arabidopsis thaliana.* (**F**) Domain architectures of *H. sapiens* YY1, *S. cerevisiae* Ies4, *C. thermophilum* Ies4, and *S. cerevisiae* Rtt102. The positions of the REPO/2W-motifs are indicated in green. (**G**) Detailed view of the Ies4-actin interface in *C. thermophilum*. The conserved tryptophan and proline residues are shown. (**H**) Detailed view of the proposed YY1-ACTB interface in *H. sapiens*. An AlphaFold multimer model of YY1-ACTB was used as guidance for rigid-body docking into the A-module density. The conserved tryptophan and proline residues are shown.

To determine the complete modular architecture of INO80 A-modules (Fig. 1A) and to gain insight into their interactions with DNA, we used cryo-EM to obtain high-resolution structures of A-modules from *C. thermophilum* and *S. cerevisiae* (Fig. 1, B and C). Structures were obtained either directly from recombinantly produced A-modules or as individually processed and refined A-module classes in cryo-EM datasets on various INO80 or INO80:nucleosome complexes (table S1). The qualities of the maps were good enough to model the polypeptide chain (Fig. 1B and fig. S1A) using previous crystal structures as starting models or de novo (Arp8 N-terminus and Ies4). The releases of AlphaFold2 (*29*) allowed us to interpret less well-defined regions of the maps, as well as interpret a medium resolution map of the *H. sapiens* A-module (Fig. 1D).

The A-modules from all three species revealed similar overall architectures and conformations (Fig. 1, B to D). As observed in a previously reported partial crystal structure of *S. cerevisiae* Ino80^HSA^-Arp4-actin-Arp8^ΔN^ (Arp8 N-terminus deletion), the cryo-EM structures showed a sequential arrangement of Arp4, actin, and Arp8 along approximately 20 helical turns of the Ino80^HSA^ domain. However, the cryo-EM analysis enabled us to define two functionally important elements of the INO80 A-module that were missing in the previous crystallographic analysis, the N-terminal extension of Arp8 (i.e., amino acids preceding the actin fold) and the Ies4 subunit (Fig. 1, B and C).

We could visualize most of the *Ct*Arp8 N-terminal extension (residues 14 to 98) with only residues 1 to 13 missing. It forms an extended yet defined chain that folds along INO80^HSA^ toward the actin fold part of Arp8, with additional contacts to Ies4, Arp4, and actin (Fig. 1B). *S. cerevisiae* Arp8’s N-terminal extension (residues 1 to 266) harbors additional 170 amino acids, which are not visible in our structure and are not an evolutionarily conserved feature. However, the conserved region of the Arp8 N-terminal extensions adopts a remarkably similar geometry and uses similar contacts along the actin folds and Ino80^HSA^, despite the lack of secondary structures, suggesting a high degree of evolutionary and functional conservation (Fig. 1, B and C).

The C-terminal part of Arp8 N-terminal extension forms a helix that binds into the interface of actin and Arp8 and thus might be affected by the nucleotide state of the actin folds. To test this, we imaged A-modules in the presence of different nucleotides (fig. S1, A to C). In the order ADP>ATPγS>ATP, we observe a very small conformational change in the *S. cerevisiae* Arp8-actin pair and an ordering of the N-terminal segment of Arp8 along actin and Arp4 in the ATP state (fig. S1, D and E). This may indicate a potential differential role of ATP/ADP at *S. cerevisiae* Arp8. Typical for actin fold proteins, the underlying conformational changes are very subtle, making it difficult to distinguish them from experimental variability in the cryo-EM analyses at this stage. In the case of *C. thermophilum* A-module, imaging without nucleotides (DNA-bound classes) or adding ATPγS (without DNA) resulted in the presence of ATP/ATPγS at the nucleotide binding sites of all three actin fold proteins (fig. S2, A and B). In any case, in both *S. cerevisiae* and *C. thermophilum* A-modules, we observed constitutive ATP binding at Arp4 and actin, while nucleotide binding to *S. cerevisiae* Arp8 is at least variable (figs. S1, A to C, and S2, A and B).

The resolution of the maps allowed us to unambiguously define and model the central part of the Ies4 subunit and define its interaction within the A-module (Fig. 1B). *Ct*Ies4^173–192^ forms a β-hairpin that binds across actin (subdomain I) and Arp4, stabilizing and fixing their mutual arrangement. The same β-hairpin structure and interaction architecture is seen in the case of *S. cerevisiae* Ies4^35–74^, despite low sequence similarity (Fig. 1C). Comparing both structures sheds light onto two tryptophan residues (2W), which emerge as key anchor points to actin and are highly conserved among Ies4 homologs (Fig. 1, E to G). While the β-hairpin element (denoted 2W-hairpin) and some flanking parts are defined in the structures, further N- and C-terminal parts of Ies4 are not resolved.

The two tryptophans bind a Gly^366^-Pro^367^ linker between the last two helices of actin (subdomain I; Fig. 1G and figs. S2C and S3A). Here, Pro^367^ is situated in an aromatic “corner” formed by the nearly right-angled tryptophan side chains. A similar type of interaction to human ACTB (β-actin) Pro^367^ through two tryptophans organized in a β-stranded structure is seen in the extracellular actin sensor C-type lectin DNGR-1, suggesting a more widely evolved actin interaction principle (fig. S3B) (*30*). Furthermore, the β-stranded fold and the presence of two tryptophans are broadly similar to the WW domains that bind proline-rich peptides (fig. S3C) (*31*, *32*).

Ies4 also interacts with the N-terminal tail of Arp8 as well as with Ino80^HSA^ (Fig. 1B and fig. S2, D and E). These contacts are mediated by the tip of the β-hairpin element and are probably important to assemble a “defined” INO80 A-module because the Arp4-actin pair is also present in BAF/PBAF, SWR1, and NuA4 complexes as part of different molecular assemblies. The 2W-hairpin motifs of Ies4 are furthermore interesting, as they resemble the structure of Rtt102 bound to Arp7-Arp9. Arp7-Arp9 are the orthologs of Arp4-actin in *S. cerevisiae* SWI/SNF family remodelers SWI/SNF and RSC (Fig. 1, E and F). Rtt102 displays a similar 2W attachment to Arp9 as Ies4 (Fig. 1E and fig. S3D), revealing an architectural conservation of A-modules across INO80 and SWI/SNF remodelers that goes well beyond the Arp4-actin pair and the HSA domain.

### Mammalian YY1 is the structural homolog of yeast and fungal Ies4

Mammalian INO80 does not have a clearly recognizable Ies4 homolog based on sequence conservation. However, YY1, a GLI/Krüppel-like transcription factor associated with chromosome loop formation, stem cell biology, and early development, has been shown to interact with a module of human INO80 containing INO80^HSA^, ACTL6A (Arp4 homolog), and ACTR8 (Arp8 homolog) (*33*). To see whether YY1 could be the evolutionary ortholog of Ies4, we produced recombinant *H. sapiens* A-module INO80^HSA^, ACTL6A , ACTB, ACTR8 , and YY1. These proteins assemble in a stoichiometric and stable complex that we used for cryo-EM analysis (fig. S4A). From 25,652 particles, we obtained a map with a resolution of 7.5 Å (Fig. 1D and fig. S4B), but a high degree of particle orientation bias impeded a higher-resolution reconstruction. Still, it allowed unambiguous interpretation with models derived from the crystal structure of ACTR8and AlphaFold2 models of ACTL6Aand ACTB. In general, the arrangement of actin-related proteins and ACTB along INO80^HSA^ is very similar to that found in fungal and yeast complexes (Fig. 1, B to D). After docking of the actin fold proteins, residual density at the hydrophobic rim of ACTL6A–ACTB matches very well the density corresponding to the hairpin region of Ies4 on the surface of yeast and fungal Arp4-actin (Fig. 1D). Sequence analysis (Fig. 1E) and AlphaFold2 prediction of YY1 indicated that residues 201 to 226 have the appropriate β-hairpin structure with two conserved, flanking tryptophans. This part has also been crystallized in a complex with the polycomb group protein MBTD1 and shows a 2W-hairpin motif (fig. S3E) (*34*). AlphaFold2 modeling of a complex of ACTL6A and the 2W-hairpin of YY1 (Fig. 1H) resulted in a complex that matches the corresponding surface density of the *Hs*A-module. Chemical cross-linking and mass spectrometry (CX-MS) also identifies a cross-link, consistent with this location of YY1 (fig. S4, C and D). Notably, binding of the 2W-hairpin motif (denoted also REPO domain) (*35*) to ACTL6A–ACTB is distinct from its interaction with MBTD1. Superposition of both complexes via the YY1 element indicates partially overlapping binding sites to the hairpin region (fig. S3F), which may explain partitioning of the *Drosophila* YY1 ortholog Pho into INO80 and *Drosophila melanogaster* polycomb group protein Sfmbt (*35*).

Besides the INO80 complex, we identified the 2W-hairpin motif in the AlphaFold2 predictions of complex subunits of INO80 family (*S. cerevisiae*: Ies4 in INO80, Swc4 in SWR1, and NuA4; *H. sapiens*: YY1 in INO80, DMAP1 in SRCAP, and TIP60) and SWI/SNF family remodelers (*S. cerevisiae*: Rtt102 in SWI/SNF and RSC; *H. sapiens*: BCL7 in BAF and PBAF) (*36*), hinting at a pervasive binding motif between remodeler families (fig. S3, G to I).

Together, we conclude that mammalian YY1 is the ortholog of fungal and yeast Ies4 and that actin (or Arp9 in the case of *S. cerevisiae* SWI/SNF and RSC) along with Arp4 orthologs (or *Sc*Arp7) recruit a REPO/2W-hairpin element protein client (YY1, Ies4, Rtt102, and others) to assemble a conserved heterotrimeric element in SWI/SNF and INO80/SWR1 chromatin-modifying complexes (fig. S3J).

### HSA^α1^ and HSA^α2^ are critical for INO80 function in yeast

**Fig. 2.**
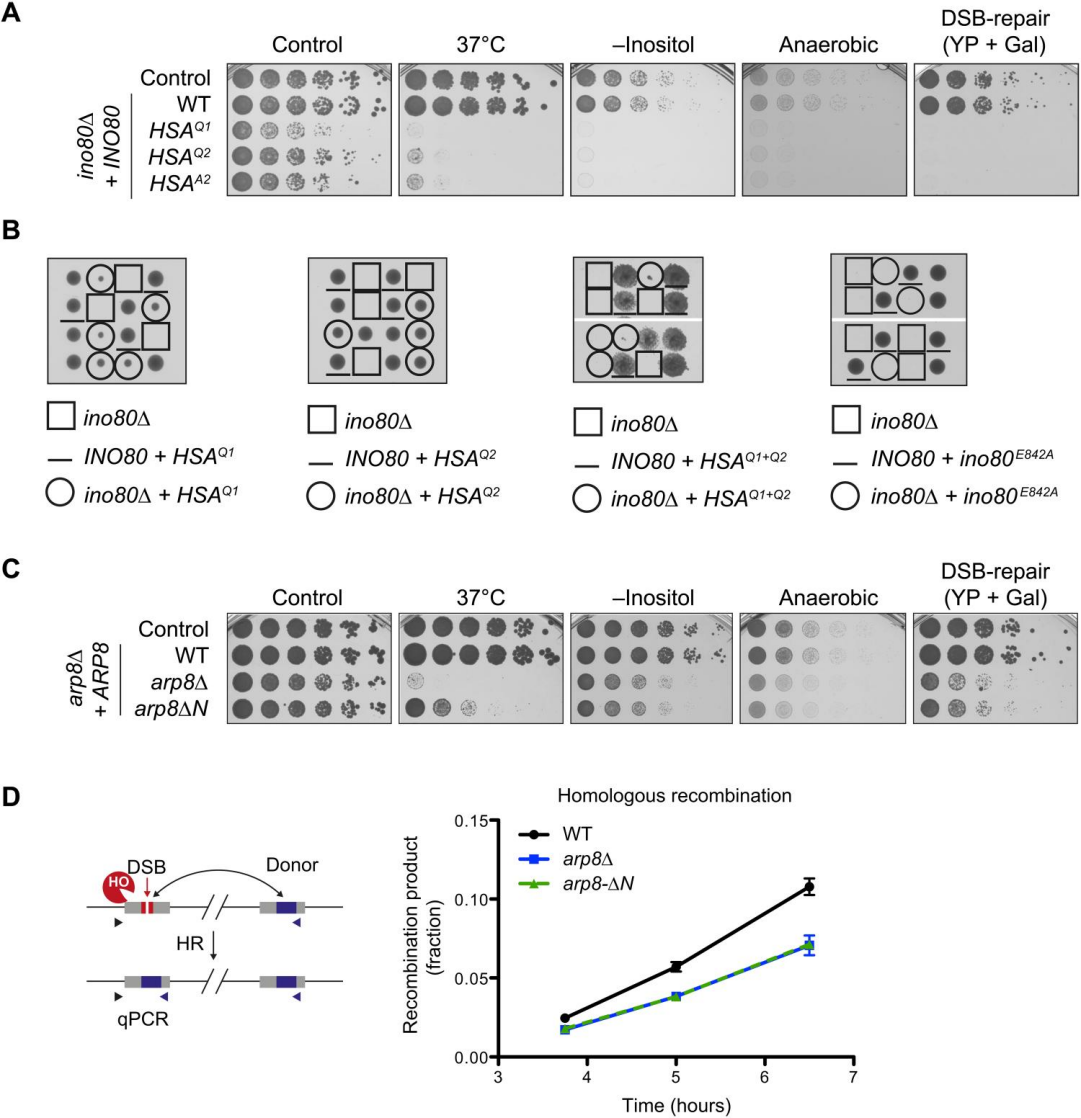
HSA surface residues are critical for INO80 function in budding yeast. (**A**) Fivefold serial dilutions of budding yeast expressing indicated Ino80 variants were grown for 2 to 7 days. (**B**) Tetrad analysis of yeast cells bearing the indicated *INO80* alleles, where each row represents four colonies of a tetrad from a single diploid progenitor cell. *ino80-HSA^Q1^* and *ino80**-**HSA*^Q2^ alleles (circled colonies in left two panels) partially rescue the *ino80*∆ lethality. The *ino80-HSA^Q1+Q2^* double mutant allele showed severely impaired or no growth (circled, third panel). The Walker B mutation (*ino80^E842A^*) is lethal (fourth panel). (**C**) The Arp8 N-terminal region is critical for tolerance to elevated temperatures (37°C), growth on medium lacking inositol, under anaerobic conditions, and for DSB repair via homologous recombination (HR). *arp8∆* cells were complemented with a full-length *ARP8* allele (WT) or an allele lacking the N-terminal 197 amino acids (*arp8∆N*) and subjected to spot dilution growth assays as in (A). (**D**) The N-terminal region is required for Arp8 function in DSB repair by HR. Left: Schematic of the quantitative real-time PCR (qPCR)–based analysis of HR (*39*). Cells express a galactose-inducible HO endonuclease that cuts a single defined HO-cleavage site (red, ChrIV 491 kb). The DSB can be repaired by HR using a noncleavable donor site as repair template (blue, ChrIV 795 kb), and HR can be quantified by amplifying a recombination-dependent PCR product (triangles indicate primer positions). Right: Emergence of the recombination product after HO endonuclease induction (*t* = 0) was normalized to completed recombination (value = 1) for the strains indicated. *n* = 3, with error bars denoting SDs.

Previous biochemical work established that the INO80 A-module is important for extranucleosomal DNA recognition and nucleosome sliding in vitro (*27*, *28*). To this end, we previously identified a series of positively charged residues on HSA^α1^ and HSA^α2^ that, upon mutation to glutamines, severely affected the nucleosome sliding in vitro (denoted HSA^Q1^ and HSA^Q2^) (*27*). We introduced these mutants, along with *arp8*Δ, *arp8ΔN* (*28*), and a Walker B mutation in Ino80 that affects ATP hydrolysis (*ino80^E842A^*) into *S. cerevisiae* (W303 background; tables S2 and S3). Because these mutants were designed before the experimental DNA complex was obtained (see below), we generated an additional set of more structure-informed (*C. thermophilum*) K/R→A mutants in *S. cerevisiae* HSA^α2^ (denoted HSA^A2^), which led to similar effects as the HSA^Q2^. We tested for viability under unchallenged conditions as well as in the presence of different stresses that had previously been linked to the INO80 function (*37*–*39*). While a wild-type (WT) *INO80* construct was able to complement the *INO80* deletion, *ino80-HSA^Q1^* gave poor growth already at unchallenged conditions and was unable to support growth upon heat stress, in the absence of inositol, under anaerobic conditions or upon induction of a DSB (DNA double-strand break; Fig. 2A; see fig. S5, A to C, for expression levels of mutant proteins). *ino80*-*HSA^Q2^* and *HSA^A2^* cells showed similar but slightly less severe phenotypes. The *ino80-HSA^Q1+Q2^* double mutant was unable to support viability in W303 background, similar to strains lacking *INO80* or the *ino80^E842A^* mutant (Fig. 2B), suggesting an additive contribution of DNA binding by HSA^α1^ and HSA^α2^. Furthermore, deletion of *ARP8* showed a growth phenotype under all stresses, but was only mildly affecting growth under nonperturbed conditions (Fig. 2C). Expression of *arp8*Δ*N* partially rescued the *arp8Δ* heat stress phenotype, but not the homologous recombination–dependent DSB repair function as tested in growth and ectopic recombination assays (Fig. 2, C and D). Together, these data validate the importance of putative DNA interacting residues of the HSA domain in rendering INO80 functional and indicate that the INO80 DNA binding surfaces might affect the diverse functional roles to different degrees.

### Structural basis of DNA binding by the INO80 A-module

**Fig. 3.**
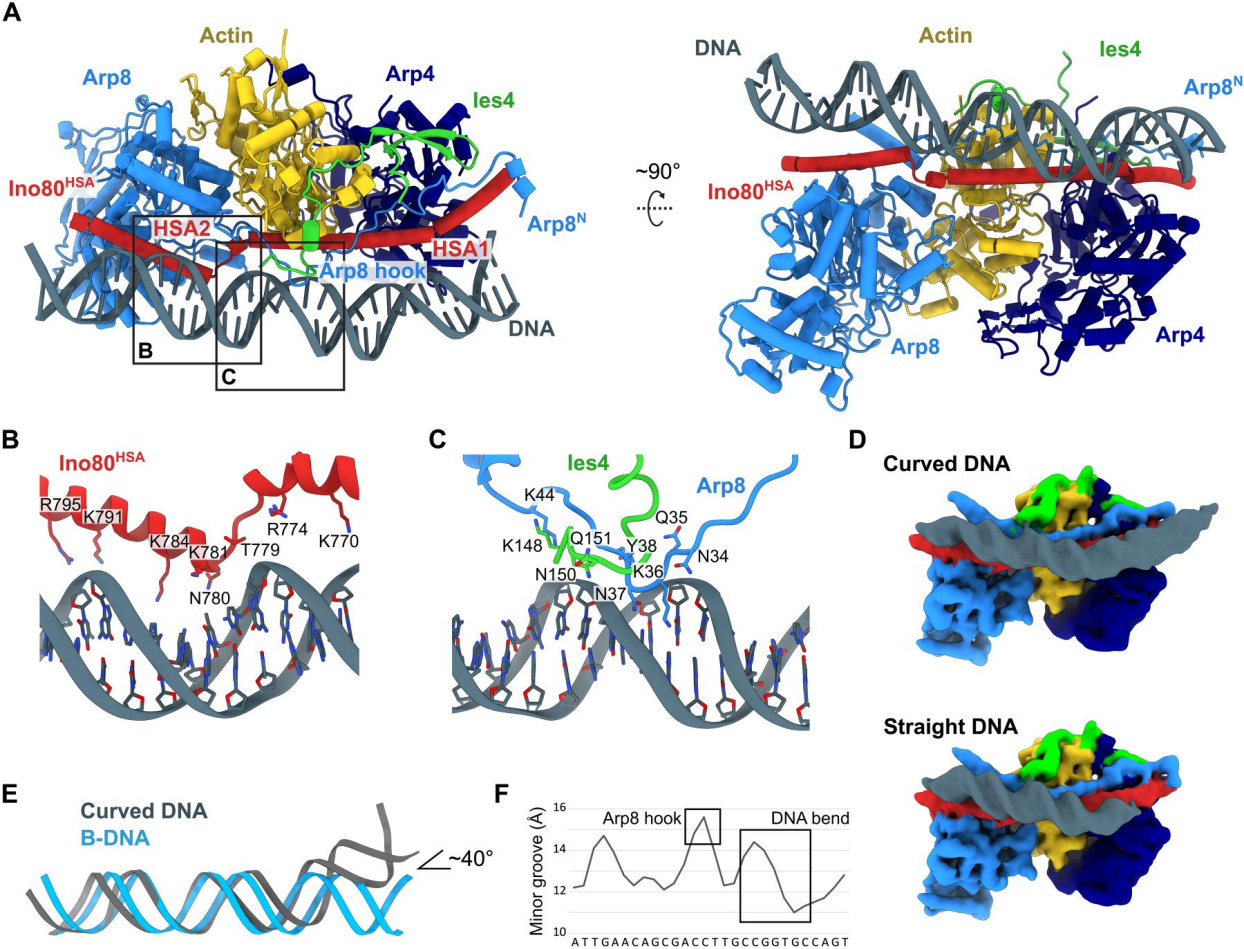
Structure of the *C. thermophilum* A-module bound to DNA. (**A**) Structural model of *C. thermophilum* A-module bound to DNA. (**B**) Detailed view of the Ino80^HSA^-DNA interaction. (**C**) Detailed view of the Arp8 hook and Ies4-DNA interaction. (**D**) Cryo-EM reconstructions of A-module bound to (top) curved and (bottom) straight DNA. Gaussian filtering was applied with a width of 1.25 (*76*). (**E**) Structural comparison of A-module bound DNA (curved DNA) and B-DNA. (**F**) Analysis of minor groove width of curved DNA (*77*). Positions of Arp8 hook interaction and the DNA bend are indicated with squares.

Having established the structure of INO80 A-module and the critical functional role of the positively charged Ino80^HSA^ surface residues in vivo, we set out to reveal the way that the A-module interacts with extranucleosomal DNA. We used a subset of two-dimensional (2D) classes in our *Ct*INO80 dataset (ADP∙AlF*_x_* and *apo*) that showed well-defined A-module:DNA complexes (Fig. 3, A to C). Using extensive 2D and 3D classification, 3D variability analysis (movie S1), and refinement, we classified and refined two states that differ somewhat in the way they bind DNA (Fig. 3D). One state was refined to 3.3-Å resolution and showed ~25-bp linear DNA. In a second state, refined to 3.4-Å resolution, additional protein DNA contacts result in binding of ~35-bp DNA that exhibits curved conformation.

In both states, most DNA interactions are formed by Ino80^HSA^, consistent with the robust effects of Ino80^HSA^ mutations in the in vivo analysis. Additional interactions are contributed by the N-terminal extension of Arp8 and by Ies4 (Fig. 3, A and C). While most Ino80^HSA^-mediated DNA interactions appear to be peripheral electrostatic interactions between Lys and Arg side chains and DNA, a central contact side is at HSA^L1^, a loop that disrupts the Ino80^HSA^ element into two helices HSA^α1^ and HSA^α2^ (Fig. 3B). Here, the N-terminal turn of HSA^α2^ binds a DNA backbone phosphate through main-chain amide nitrogens. This interaction shows a remarkable similarity to the DNA interactions of the innate immune sensor cGAS and could provide a phosphate “registry lock” (*40*). The central contact side is reinforced by a “hook” element of the Arp8 N-terminus that binds to the DNA backbone as well as to two minor groove base pairs (Fig. 3C). The hook element is stabilized by Ies4 (residues 148 to 156), which is also in direct binding distance to the DNA backbone and may contribute further interactions. Similar folds of the hook region in the *apo* states of the *C. thermophilum* and *S. cerevisiae* A-modules suggest evolutionary conservation of this DNA binding element.

In the case of curved DNA, we also observe DNA contacts around SHL-11, mediated predominantly via the HSA^α1^ region and a helix near the very N-terminus of Arp8^N^ (Fig. 3D). Binding of curved DNA is noteworthy, as it might be influenced by DNA mechanical properties. Geometrically, it is a result of the curved shape of Ino80^HSA^ at the Arp4-actin pair, which is incompatible with binding of linear DNA along the entire length of the A-module (Fig. 3, E and F).

A 7.5 Å resolution structure of *S. cerevisiae* A-module bound to DNA could also be reconstructed from 69,226 particles (fig. S1F). Here, we see predominantly contacts at HSA^α1^ and the Arp8 N-terminal extension. Again, DNA appears to be curved at this side, but the rather low resolution prevents a more detailed analysis. Predominant binding of DNA at HSA^α1^ is consistent with the somewhat stronger growth defects of HSA^Q1^ mutations in *S. cerevisiae* in vivo (Fig. 2A).

In summary, we provide a structural mechanism for extranucleosomal DNA binding of the INO80 A-module, revealing multiple DNA contact sites along the entire A-module and the possibility to interact with both curved and linear DNA through a modular set of interaction sites.

### Biochemical analysis

**Fig. 4.**
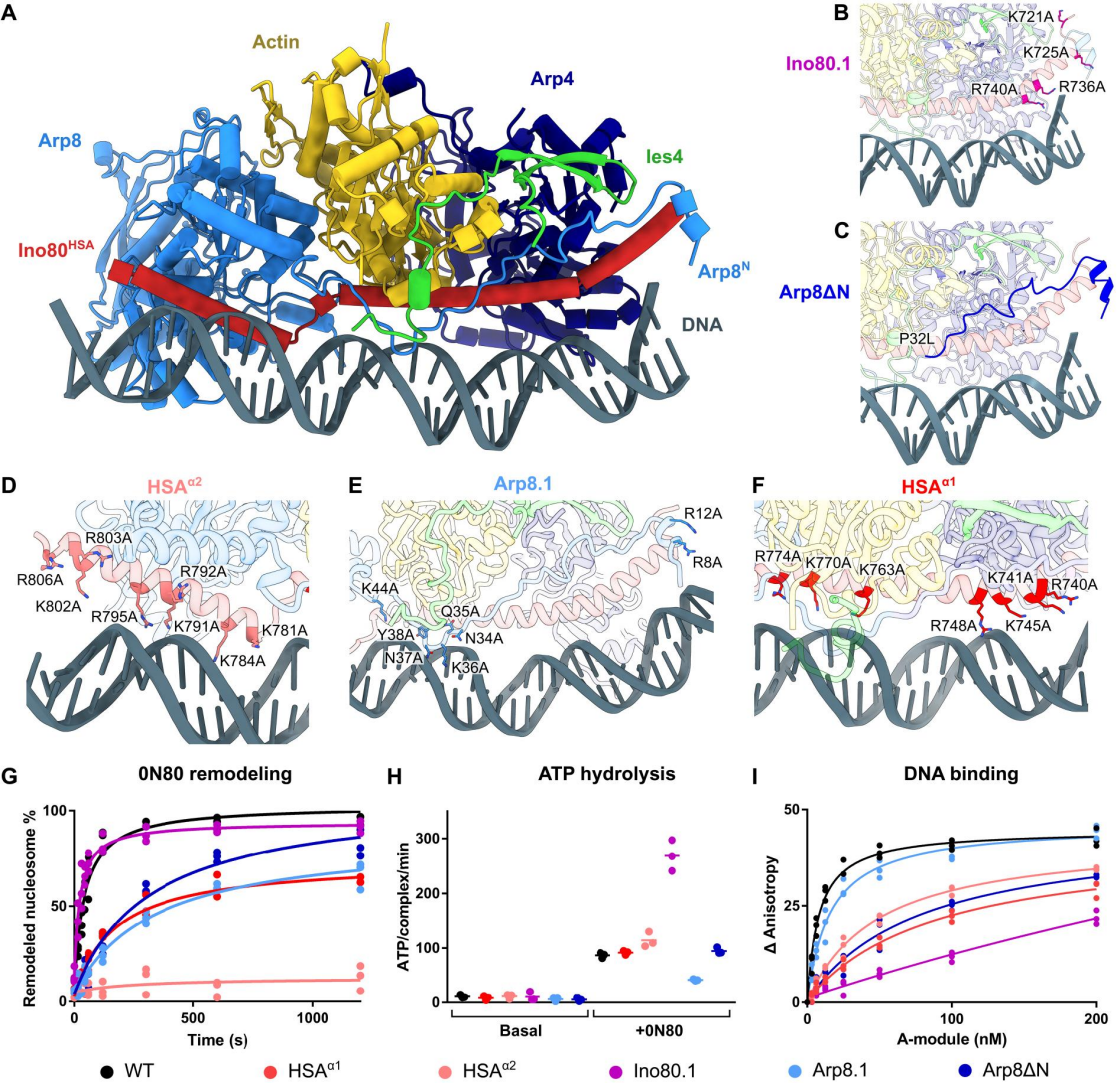
Structural basis of DNA binding by the INO80 A-module. (**A**) Structural model of *C. thermophilum* A-module bound to DNA. (**B**) Ino80.1 mutation probes the distal region of HSA^α1^. (**C**) Illustration of the truncated portion of the Arp8 N-terminus; P32L truncation site. (**D**) HSA^α2^ mutations. (**E**) Arp8 mutations in hook and N-terminal helix. (**F**) HSA^α1^ mutations probe the central region of HSA^α1^. (**G**) Evaluation of the remodeling activity of *Ct*INO80^ΔN^ mutants. Band intensities of remodeled and unremodeled nucleosome species were quantified, and the fraction of remodeled nucleosomes was plotted against time. Data points were fitted using an exponential equation. Mean and individual data points (*n* = 3, technical replicates). (**H**) ATPase rate of *Ct*INO80^ΔN^ mutants with and without stimulation by nucleosomes. Rates were calculated from the linear area of the raw data and were corrected by a buffer blank. Mean and individual data points (*n* = 3, technical replicates). (**I**) Fluorescence anisotropy assay to monitor the binding of *C. thermophilum* A-module and mutants to a 50-bp DNA. The data were fitted to a nonlinear noncooperative 1:1 binding model. Individual data points of three independent experiments are plotted.

The observation that *Ct*INO80 A-module can bind both linear and curved DNA prompted us to perform more detailed biochemical studies to analyze the role of different DNA binding sites (Fig. 4, A to F) on *Ct*INO80^ΔN^ remodeling (Fig. 4G and fig. S6, A and B), *Ct*INO80^ΔN^ ATP hydrolysis (Fig. 4H and fig. S6C), and the A-module DNA binding activities (Fig. 4I and fig. S6D) in vitro. To this end, we evaluated various structure-derived mutations in the Ino80^HSA^ and Arp8 subunit (fig. S6, B, E, and F).

Mutations in HSA^α1^ (R740A, K741A, K745A, R748A, K763A, K770A, and R774A) and HSA^α2^ (K781A, K784A, K791A, R792A, R795A, K802A, R803A, and R806A) or truncating of the Arp8 N-terminal extension (Arp8^ΔN^) did not significantly influence the ATPase rate of *Ct*INO80^ΔN^ but reduced (HSA^α1^, Arp8^ΔN^) or nearly abolished (HSA^α2^) nucleosome sliding. They also reduced the A-module DNA binding efficiency (Fig. 4, G to I). This suggests that DNA contacts of the HSA domain add proper grip or induce a particular geometry to couple ATP hydrolysis cycles with nucleosome sliding. The severe effect of the HSA^α2^ mutant in sliding, but moderate effect in DNA binding, argues for a geometric function at least for this region, but does not rule out a function as grip as well.

Arp8.1 (N34A, Q35A, K36A, N37A, Y38A, and K44A), carrying mutations in the hook as well as the N-terminal helix, leads to a reduction in sliding in the same range as Arp8^ΔN^, but this effect appears to be caused by defects other than a simple reduction of DNA affinity (Fig. 4, G and I). Again, this argues for a defective geometry of the active complex or a particular conformational state. The most remarkable effect showed the Ino80.1 mutant (K721A, K725A, R736A, and R740A), which carries mutations in the very distal extranucleosomal DNA binding region of Ino80^HSA^. Ino80.1 strongly reduces binding to the DNA but increases sliding and ATPase rate of *Ct*INO80^ΔN^ (Fig. 4, G to I). These effects could be explained if the A-module can also negatively regulate INO80 and that such a role is affected by the Ino80.1 mutation.

In summary, the mutations all affect various functions and the biochemical properties of INO80, validating our structural results. However, they indicate that the A-module plays a more complex, pleiotropic regulatory role with activating and inhibitory roles on remodeling.

### Overall structure of INO80 A- and C-modules bound to a nucleosome

**Fig. 5.**
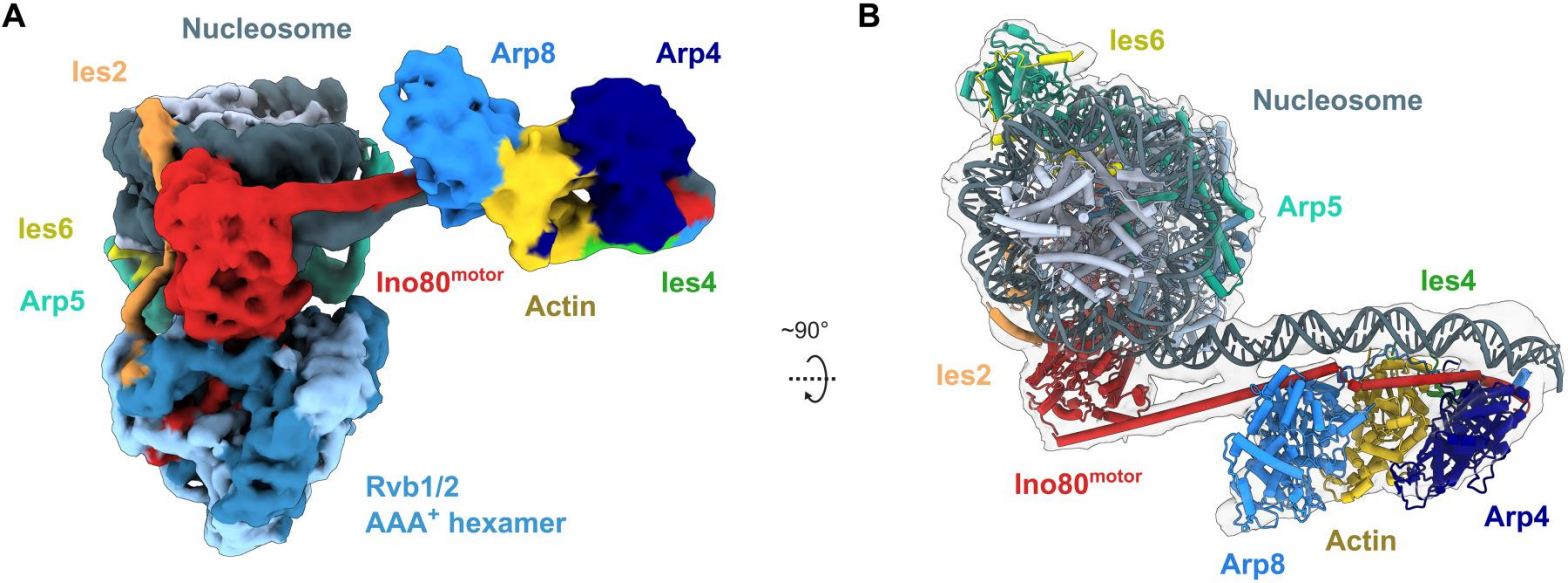
The INO80 A-module interacts with extranucleosomal DNA. (**A**) Cryo-EM reconstitution (multibody refined) of the *C. thermophilum* A- and C-modules binding to nucleosome and extranucleosomal DNA. (**B**) Structural model of INO80^ΔN^ based on structures of C-module bound to the nucleosome and A-module.

The complex regulation of nucleosome sliding by the INO80 A-module suggests an intricate regulatory coupling between A- and C-modules (Fig. 5, A and B). To reveal how the A-module could chemomechanically communicate with the C-module, we recorded and analyzed various datasets of *Ct*INO80^ΔN^ bound to 0N80 nucleosomes in the absence and presence of the ATP analog ADP∙BeF*_x_*. We used masking, particle subtraction, and focused refinement procedures to obtain well-resolved maps at various regions of the complex. Aided by AlphaFold2 modeling of structural elements, we could substantially improve our previous analysis (*20*, *27*) and add previously missing parts such as the architecture of the grappler and the post-HSA domain bound to Ino80^motor^.

**Fig. 6.**
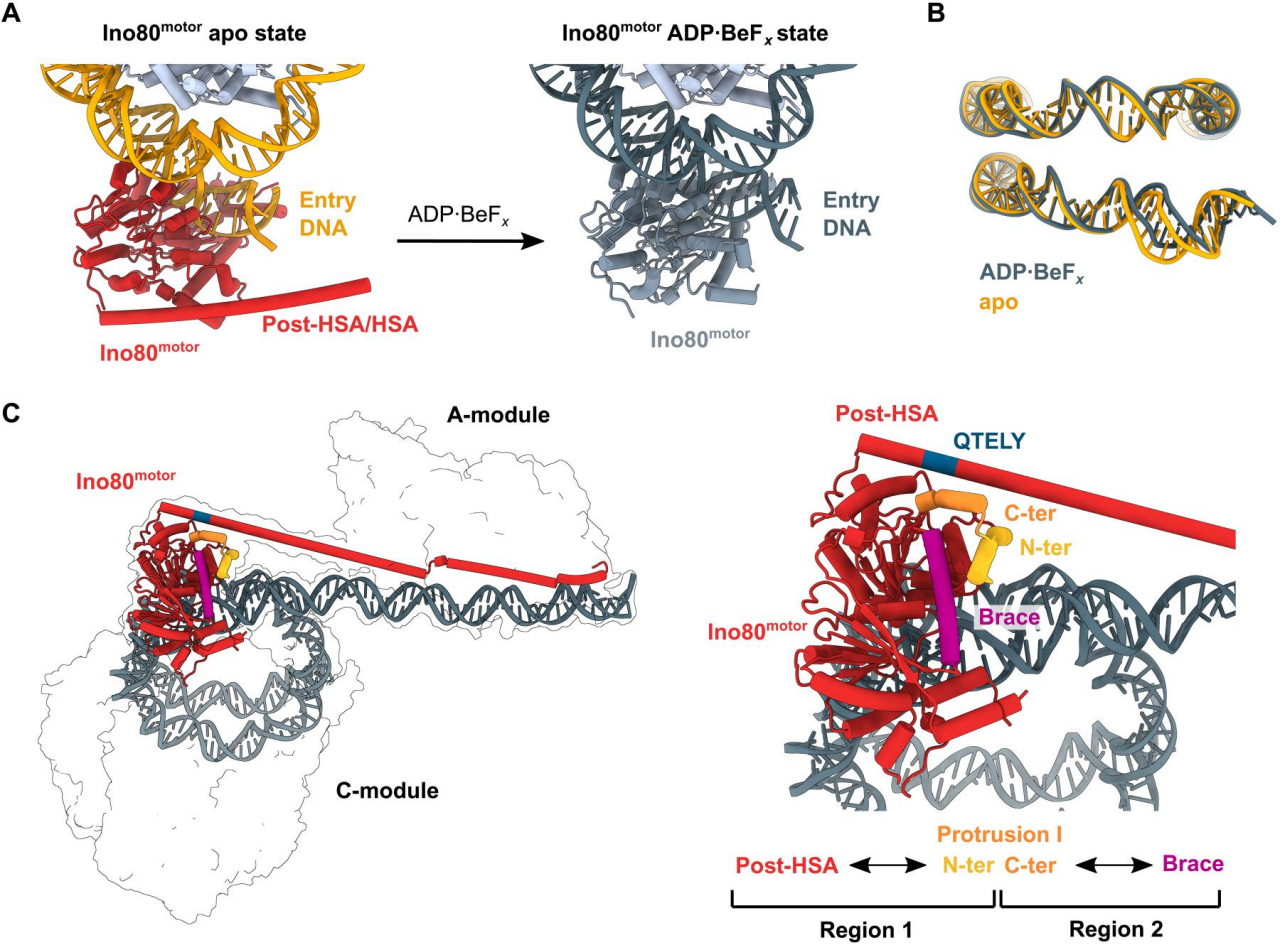
Ino80^motor^ conformations in *apo* and ADP∙BeF*_x_* states. (**A**) Structural model of *C. thermophilum* Ino80^motor^ interacting with the nucleosome at SHL-6 in *apo* state (left) and ADP∙BeF*_x_* state (right). Structured post-HSA domain is visible in *apo* state. (**B**) Comparison of the nucleosomal DNA in *apo* state (orange) and ADP∙BeF*_x_* state (gray). (**C**) Structural model of the A- and C-modules bound to nucleosome and extranucleosomal DNA. Ino80^motor^ and post-HSA/HSA (red) and nucleosomal DNA (dark gray) are highlighted. The N-terminal helix of protrusion I engages the post-HSA (region 1), whereas the C-terminal protrusion I helix contacts the brace (region 2). The conserved QTELY motif forms the post-HSA contact site toward protrusion I.

We first focused on the motor domain to see what effects ATP binding have on the way Ino80^motor^ interacts with DNA. In the *apo* state (no nucleotide), Ino80^motor^ is well resolved and substantially bends DNA as previously described (Fig. 6A). In the *apo* state, we now see clear density for the post-HSA domain, which was missing in previous analyses. It interacts as a continuous, long helix with the N-lobe of the motor domain. It occupies the same region on the motor as the regulatory elements auto-N of ISWI and the post-HSA domain of Snf2 (*41*, *42*), showing a high degree of conservation of motor regulatory elements among different remodelers (fig. S7A). However, we note that the interactions of post-HSA domains of Ino80 and Swi2 are somewhat shifted, although other parts of the motor superimpose and match very well. It was previously suggested that movements of the post-HSA could be coupled to motor activation (*42*, *43*).

ADP∙BeF*_x_* binding leads to a straightening of the DNA at the motor compared to the bent conformation with widened minor groove in the apo state (Fig. 6, A and B; fig. S7, B and C; and movie S2). Furthermore, in the ADP∙BeF*_x_* bound state, the post-HSA domain is not visible anymore, suggesting that it is a rather dynamic feature that could be coupled to the nucleotide state of the motor and/or the relative location of the A-module with respect to the motor (see below; Fig. 5A).

Comparing the nucleotide-free with the ADP∙BeF*_x_* bound state, we observe a conformational transition in Ino80^motor^ that is very similar to what has been described for Snf2 and ISWI bound to the nucleosomes (*41*, *42*). Upon transitioning from ADP∙BeF*_x_* to the *apo* conformation, a step that could resemble ATP hydrolysis and ADP + P_i_ release, extranucleosomal DNA is rotated and pulled toward the nucleosome, consistent with one part of a translocation step.

In any case, the dynamics of the post-HSA motor contacts point toward a more profound allosteric communication between A- and C-modules on both ends of the HSA/post-HSA domain, and we set out to obtain an overall structure to see how A- and C-modules could communicate. While A- and C-modules appear to be generally mobile with respect to each other in most picked particles, we could identify a subset of particles in the dataset without nucleotide addition that showed a more defined orientation between the A- and C-modules. This set of particles resulted in a 7.7 Å map that allowed us to place high-resolution structures of A- and C-modules and model the entire HSA/post-HSA helix that links A- and C-modules (Fig. 5, A and B). In this structure, the A-module is situated at SHL-9 to SHL-11, orientated such that Arp8 faces the Ino80^motor^ domain, while Arp4 points away. The HSA/post-HSA region forms a continuous helix all the way from Arp8 to the N-lobe of the motor domain. In this state, the Ino80^HSA^ domain could even use further DNA contacts between Ino80^motor^ and Arp8, supported by several Lys/Arg side chains in the vicinity of DNA. Because of the structural flexibility and moderate resolution of this state, the DNA grooves are not well defined. However, modeling canonical B-DNA into the map indicates that the A-module is not exactly bound to DNA in the same way as we observe in the high-resolution individual reconstructions. It appears to be shifted along DNA by approximal ^1^/_2_ helical turn, suggesting that the A-module might not be fully engaged and aligned with the DNA grooves in this state as observed on most of the DNA-bound classes of the A-module alone (Fig. 3, A to C). The observed configuration could be a nucleosome “sliding” state, where loose DNA binding of the A-module does not slow down nucleosome sliding, yet promotes post-HSA motor contacts. Such an interpretation would be consistent with the observation that some mutants in the HSA/A-module actually lead to a speeding up of nucleosome sliding, while others slow down or abolish sliding. In the ADP∙BeF*_x_* dataset, we were not able to identify an equivalent subset of particles with well-defined arrangements of A- and C-modules. The absence of the post-HSA domain density may suggest a more dynamic mutual orientation of A- and C-modules. From sterically considerations, the different angle of entry DNA at the motor in the ADP∙BeF*_x_* state may not allow mutual binding of the A-module to DNA along with a linear HSA/post-HSA domain between A-module and motor. It is therefore possible that post-HSA–motor interactions are quite dynamic in the ATPase cycle or additional conformations of HSA/post-HSA and their attachment to the motor exist. Recent functional and structural studies on the RSC complex (*25*) identified an intriguing structural arrangement of the post-HSA domain at protrusion I of the motor. Considering the conserved arrangement of these regulatory domains in Swi2/Snf2 ATPases, it is likely that an equivalent regulatory hub exists in INO80. Notably, the conserved QTELY motif, a homolog of the conserved SWI/SNF QTXX[F/Y] motif, forms the post-HSA contact site toward protrusion I, hinting at a critical interface for modular allostery by the A-module (Fig. 6C). Together, the mode of interaction between A- and C-modules through HSA/post-HSA, and its modulation by nucleotide binding at Ino80^motor^, provides an obvious direct chemomechanical link between Ino80^motor^ and binding of the A-module to extranucleosomal DNA.

### The Arp5 grappler interacts with entry DNA and regulates the motor domain

**Fig. 7.**
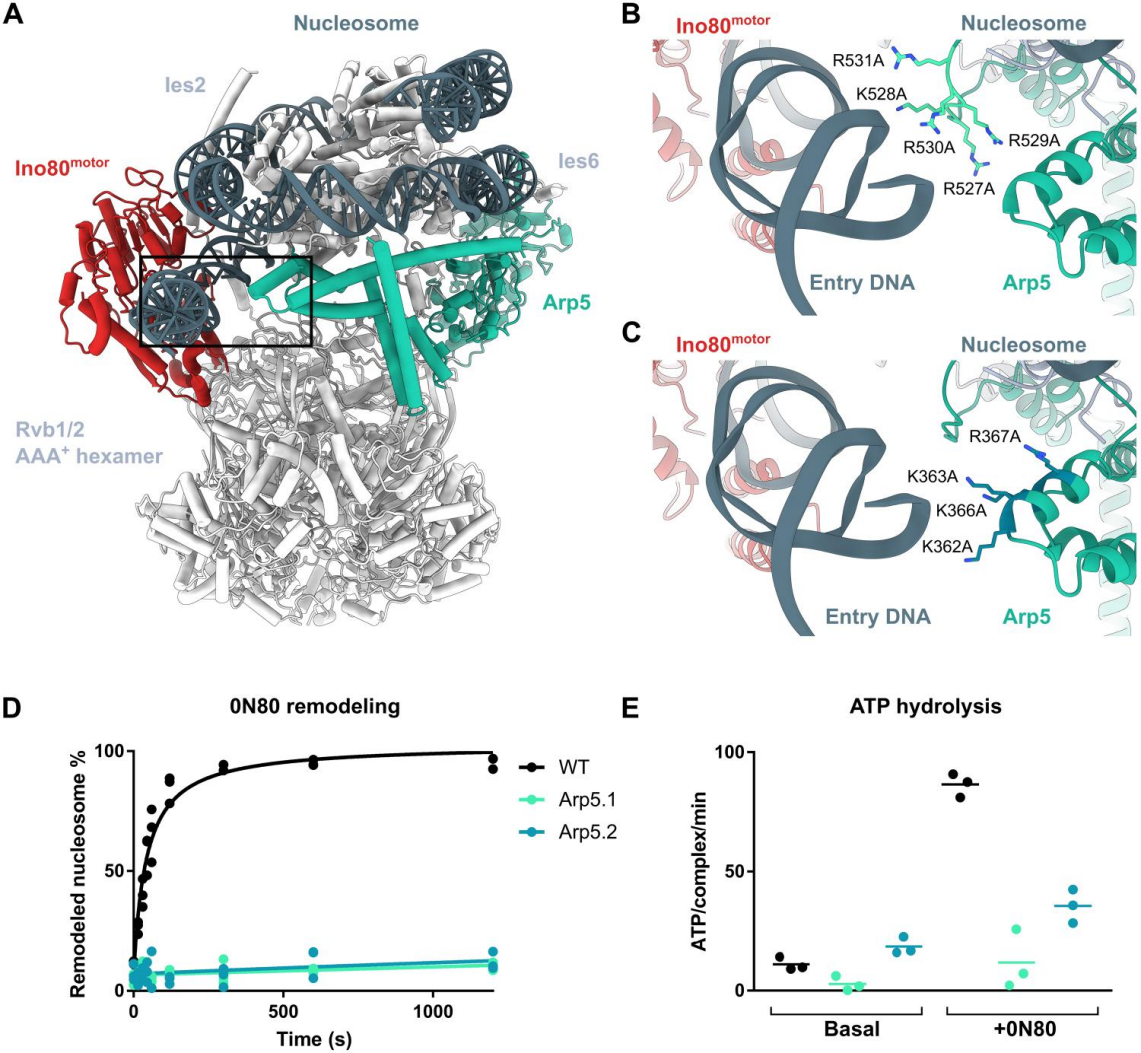
The Arp5 grappler interacts with entry DNA and regulates the motor domain. (**A**) Structural model of the *C. thermophilum* C-module, highlighting Arp5 (green, in cross configuration), Ino80^motor^ (red), and nucleosomal DNA (dark gray). (**B** and **C**) Docking model of two loop regions at or near entry DNA contains patches of Arg/Lys residues, suggesting that they form entry DNA contact sites. (**D**) Evaluation of the nucleosome sliding activity of *C. thermophilum* INO80 Arp5 grappler mutants. Band intensities of remodeled and unremodeled nucleosome species were quantified, and the fraction of remodeled nucleosomes was plotted against time. Data points were fitted using an exponential equation. Mean and individual data points (*n* = 3, technical replicates). (**E**) ATPase rate of *C. thermophilum* INO80^ΔN^ and mutants with and without stimulation by nucleosomes. Rates were calculated from the linear area of the raw data and were corrected by a buffer blank. Mean and individual data points (*n* = 3, technical replicates).

The improved maps and AlphaFold2 structure predictions allowed us to model the complete Arp5 protein, in particular its unique grappler insertion element (Fig. 7A). This led to clarification of the way the grappler “foot” binds the acidic patch of the nucleosome and allowed us to identify two additional critical DNA contacts (fig. S8A). As described previously (*20*), we observe two remarkably distinct grappler configurations (fig. S8, B and C). In the “parallel” state, its two main helical arms are arranged in a near-parallel fashion and bind DNA around the nucleosome dyad. In the “cross” configuration, one helical arm binds along the DNA gyre, placing its tip at the entry DNA opposite the motor domain. Using 3D variability analysis (movie S3), formation of contacts between the tip of the cross arm appears to coincide with a better-defined HSA/post-HSA and a properly curved entry DNA, suggesting a functional link. We noticed two patches of Arg/Lys residues in loop regions that are properly placed to interact with the entry DNA and may account for this effect. Although the density map is not good enough to directly visualize these loops, the supporting helical elements are nevertheless defined well enough to confidently provide a location for the positively charged loops using AlphaFold2 models (Fig. 7, B and C).

We generated two sets of point mutations in these Arp5 loop regions, Arp5.1 (R527A, K528A, R529A, R530A, and R531A) and Arp5.2 (K362A, K363A, K366A, and R367A; Fig. 7, B and C), and analyze their effects on nucleosome remodeling (Fig. 7D and fig. S8D) and ATP hydrolysis (Fig. 7E and fig. S8E). Both sets of Arp5 mutations nearly abolished nucleosome sliding activity and led to a markedly reduced ATPase rate of *Ct*INO80^ΔN^. This might indicate a functional interplay between Ino80^motor^ and the grappler on opposing sides of the entry DNA, enabling a geometry necessary for proper activation of Ino80^motor^, or by stabilizing the “unwrapped” (from H3/H4) geometry of entry DNA.

### Regulation of remodeling by DNA features

**Fig. 8.**
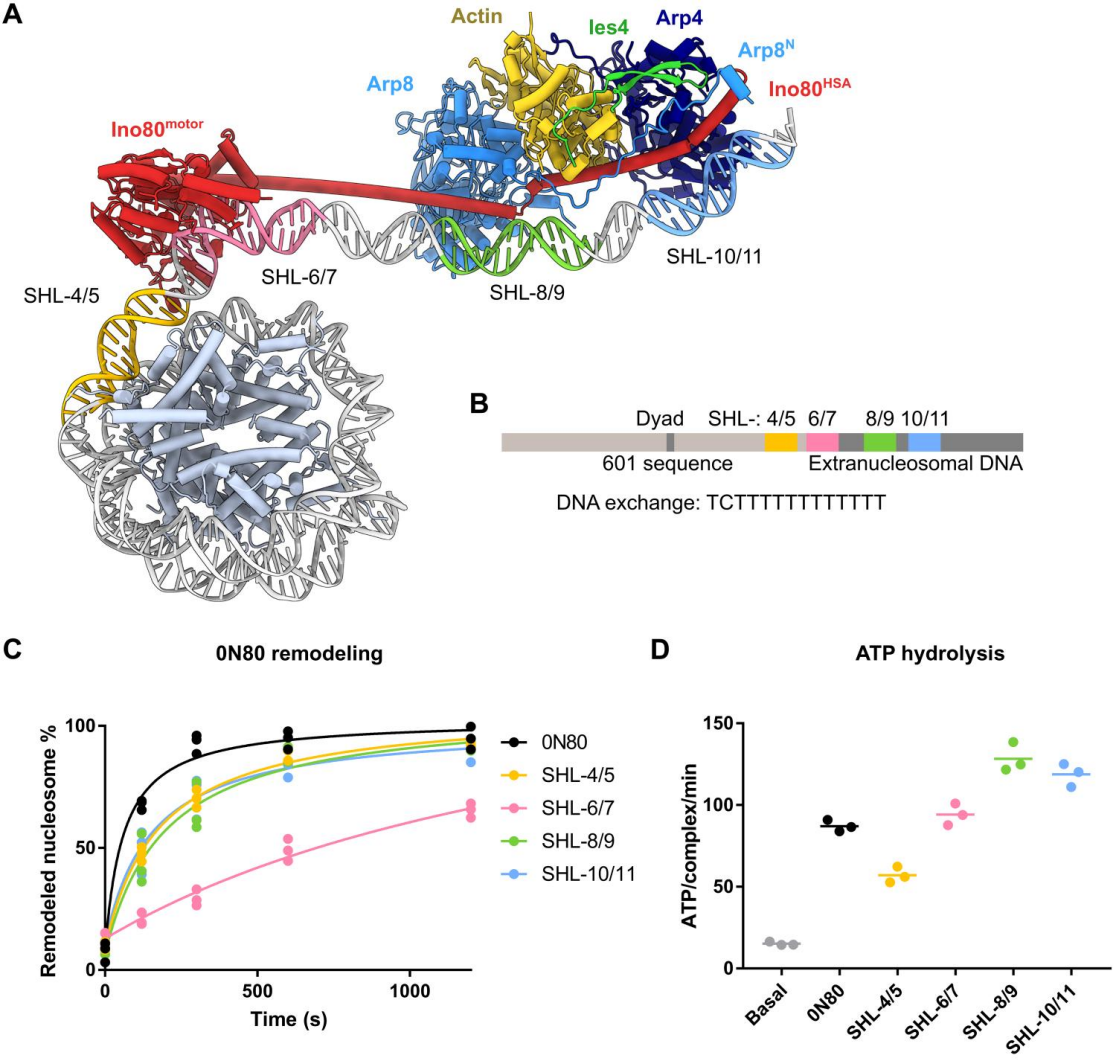
Influence of A/T-rich DNA on *Ct*INO80^ΔN^ nucleosome remodeling. (**A**) Location of exchanged DNA cassettes on the structural docking model. (**B**) Schematic visualization of exchanged DNA sequence cassettes in distance to the dyad of the nucleosome. (**C**) Evaluation of the sliding activity of *Ct*INO80^ΔN^ with different nucleosomal substrates. Band intensities of remodeled and unremodeled nucleosome species were quantified, and the fraction of remodeled nucleosomes was plotted against time. Data points were fitted using an exponential equation. Mean and individual data points (*n* = 3, technical replicates). (**D**) ATPase rate of *Ct*INO80^ΔN^ with and without stimulation by different nucleosomes. Rates were calculated from the linear area of the raw data and were corrected by a buffer blank. Mean and individual data points (*n* = 3, technical replicates).

Both the path of DNA around Ino80^motor^/Arp5 and the A-module show curved DNA regions, which are geometrically linked with the relative placement of A- and C-modules and a linear HSA/post-HSA helix, or the binding of extranucleosomal DNA along the entire Ino80^HSA^ domain. Previous experimental and statistical analysis indicated that the *S. cerevisiae* INO80 remodeling activity is influenced by DNA shape/mechanical features in extranucleosomal DNA. To test the generality of these observations for the *C. thermophilum* complex and also clarify the contribution of different modules of INO80 to DNA feature readout, we replaced the sequence of our model substrate with an A/T-rich, rigid sequence cassette derived from the *URA3 *promoter at four different locations (Fig. 8, A and B, and fig. S9A), probing contributions of distal (SHL-10/11) and proximal (SHL-8/9) extranucleosomal DNA binding sites of the A-module and the motor domain (SHL-6/7) and inside the nucleosome behind Ino80^motor^ (SHL-4/5).

Rigid DNA at SHL-8/9 and SHL-10/11 and inside the nucleosome (SHL-4/5) had a nearly equal, moderately reducing effect on nucleosome sliding by the *Ct*INO80^ΔN^ complex, whereas changing the DNA at the Ino80^motor^ binding site more markedly reduced sliding efficiency (Fig. 8C and fig. S9B). The sliding efficiencies did not correlate well with alterations in the ATPase rates of *Ct*INO80^ΔN^ because only the SHL-4/5 insertion had a reduced ATPase rate, while all nucleosome variants showed similar binding efficiency (Fig. 8D and fig. S9, C and D). ATP hydrolysis by Ino80^motor^ and sliding efficiency were also not correlated in the analysis of A-module mutations, hinting toward futile ATP cycles when stiff DNA is located at Ino80^motor^ or in extranucleosomal DNA. When DNA is inserted into the nucleosome, it is plausible that the underlying nucleosome is structurally weakened, leading to proficient sliding despite reduced ATPase rates. It should be noted that in this analysis, DNA elements are obviously pushed along different DNA binding sites during the remodeling reaction, and thus, the effects might be, to some extent, integrated. Nevertheless, the strongest effect is observed at the motor domain, which is also the site where DNA shows the most profound bend. In summary, these data show that inserting DNA cassettes with a stiff DNA sequence leads to a general reduction of nucleosome sliding, not only corroborating the influence of extranucleosomal DNA sequence but also revealing that, in particular, the motor domain is sensitive to DNA features.

## DISCUSSION

In the past years, groundbreaking structures of different remodelers bound to the nucleosome shed light on the basic principles of nucleosome recognition (*18*–*25*), while structural and functional analyses of selected single-subunit remodelers (*4*, *19*) suggested paths of allosteric activation by core nucleosome binding. While we begin to understand from these studies how remodelers grip and move DNA at nucleosomes, revealing the large-scale nucleosome reconfiguration steps and their regulation at atomic detail is the next frontier. For instance, the INO80 complex shows pleiotropic biochemical activities such as nucleosome spacing and editing, as well as the positioning of nucleosomes at NFR flanking regions (*12*). These diverse reactions depend on a basic nucleosome or hexasome sliding/mobilization activity (*11*), where the ATPase motor pumps extranucleosomal entry DNA into the nucleosome. To place a nucleosome at the +1 position, rather than sliding it further into the NFR, however, requires a regulation of the ATPase activity itself or the coupling between Ino80^motor^ and nucleosome sliding. Regulatory signals could arise when the remodeler encounters a neighboring nucleosome, a barrier factor at the NFR/NDR, and, at least in the case of *S. cerevisiae*, DNA with particular mechanical or shape features such as those found in NFRs/NDRs (*15*–*17*). Functional and previous structural work suggested that a key regulative principle is the sensing of extranucleosomal DNA by the INO80 A-module (*15*, *16*, *27*, *28*).

**Fig. 9.**
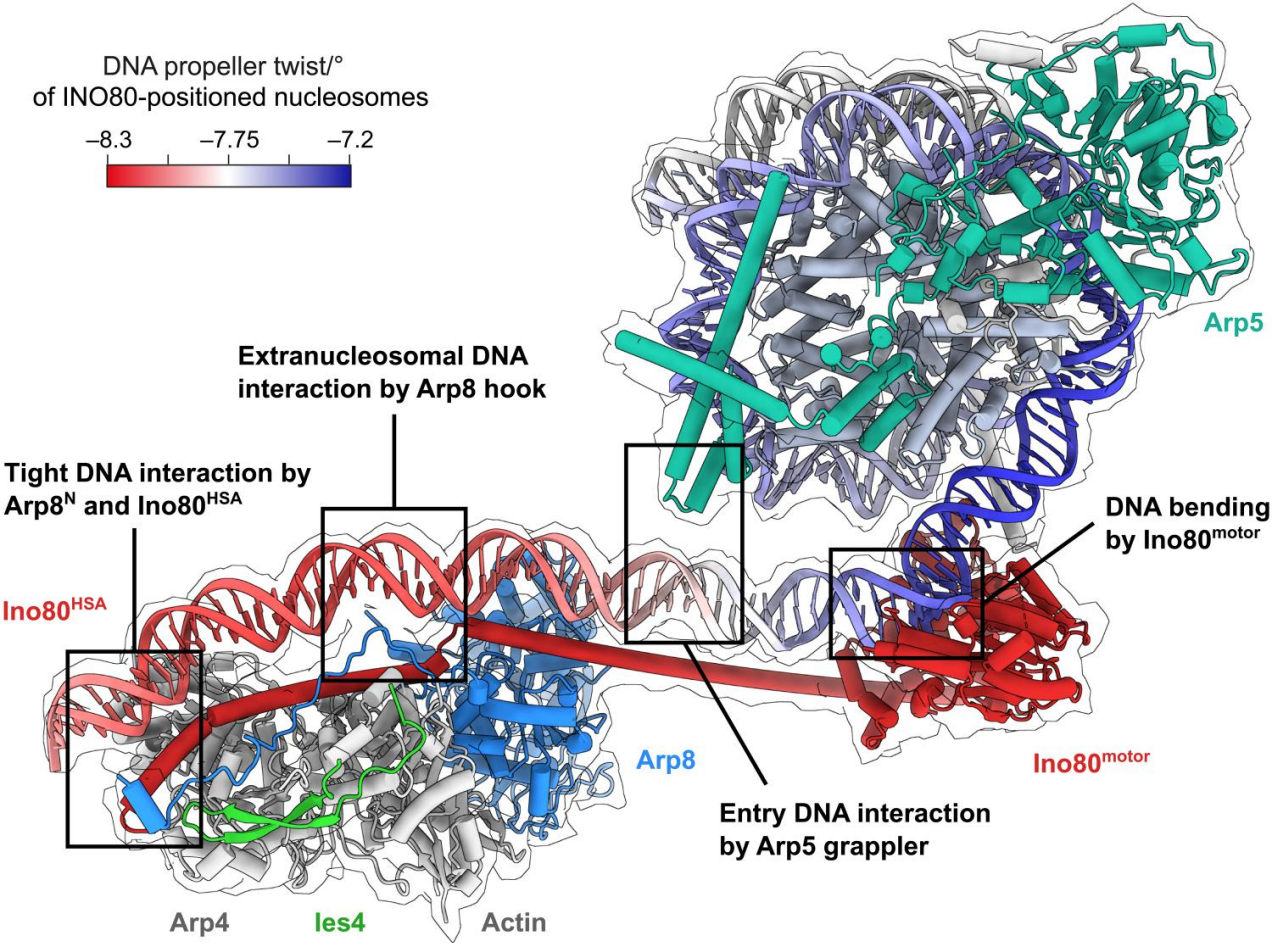
Model of multivalent INO80^ΔN^-DNA interactions. The unified model integrates our structural and biochemical analysis. Ino80^motor^ engages the nucleosome at SHL-6. The Arp5 grappler contacts entry DNA opposite of Ino80^motor^. The A-module binds extranucleosomal DNA and is linked to Ino80^motor^ via the post-HSA/HSA domain. Propeller twist DNA shape data of INO80-positioned nucleosomes (*15*) were mapped onto model of linker and nucleosomal DNA by using red-white-blue color gradient.

Here, we provide a structural basis for this regulation and reveal how INO80 interacts simultaneously with nucleosomal and extranucleosomal DNA (Fig. 9). This work extends the analysis of multisubunit remodelers from NCP binding to recognition of linker DNA and reveals how binding of extranucleosomal DNA by the A-module is chemomechanically coupled to the remodeling motor. We provide details of the linker DNA binding and identify multiple sites where DNA shape features might tune the biochemical activity. Hereby, in particular, not only do Arp8 and Arp5 subunits emerge as critical regulators but also the Ino80^motor^ domain itself might play a central role in DNA feature readout. These properties of the motor domain could provide an explanation for the unique way INO80 has evolved to interact with the nucleosome, compared to other remodelers (*5*).

The first important outcome of our analysis is the extension of the A-module architectures from the previously recognized Arp4-actin-Ino80^HSA^ core element to include highly diverse REPO/2W-hairpin–containing client proteins. The similarity of the INO80 Ies4/YY1 subunits with the RSC Rtt102 subunit (*44*) with respect to the 2W-hairpin and the apparent exceptional conservation of the 2W-motif identify a conserved nuclear actin anchor that is evolutionarily conserved among INO80/SWR1 and SWI/SNF family remodeler (Fig. 1E). Although still lacking structural evidence, the human BAF complex subunit BCL7A is predicted by AlphaFold2 to harbor a similar 2W-motif (fig. S3I), further unifying A-module compositions across INO80 and SWI/SNF-type remodeler. The 2W-hairpin bears similarity to the abundant and structurally well-characterized WW domains (*45*), which binds proline-rich regions of their target proteins (*31*, *32*). The classic WW domain is predominantly found in protein complexes involved in cell signaling, most prominently in the Hippo pathway (*46*). The two-stranded β-sheet in Ies4 and YY1 comprises two conserved tryptophans, but they assemble on opposing sites on the respective β-strand and form a hydrophobic pocket that accommodates one proline of an actin helix-turn motif. This minimal WW domain, which we denote 2W-hairpin, was described in Rtt102, where it tethers it to RSC A-module constituents Arp7/Arp9 (*25*, *44*).

The 2W-hairpin also provides an interesting, unanticipated connection between DNA sequence feature recognition and remodeler regulation. Mammalian YY1 and *Drosophila* Pho both have additional DNA binding domains, which are absent in, e.g., Ies4 and Rtt102 orthologs/paralogs. Ies4 is linked to roles of INO80 in the DNA damage response (*47*, *48*) and in targeting to centromeric chromatin (*49*) but does not have recognizable DNA binding domains such as YY1 and Pho. We speculate that while the Arp4-actin-Ino80^HSA^ module serves as a core regulator of remodelers, the REPO/2W-hairpin clients provide a rapidly evolvable, variable adaptor to add remodeler-specific and species-specific regulatory and/or targeting features to the core A-module.

In YY1, the 2W-hairpin was characterized as the REPO (recruitment of polycomb) domain because it facilitates recruitment of polycomb group complexes (PcGs) (*34*). This mirrors the bivalent nature of YY1’s context-dependent transcriptional activation and repression: YY1 recruits either activating (INO80) or repressive (PcG) complexes to their respective genomic loci dependent on the cellular context. As an integral subunit of the *Hs*INO80 complex, YY1 has been implicated in the recruitment of INO80 to promoter sites. A coactivation between YY1 and INO80 was proposed because, as a transcription factor acting on accessible DNA, YY1 also relies on the INO80 nucleosome sliding activity to gain access to its cognate promoter sites (*33*). Epigenetic regulation of YY1 binding to DNA could also influence INO80 recruitment. YY1 binding is inhibited by methylation of certain CpG sites (*50*, *51*), which might conceivably control INO80 engagement, or activity, at promoter sites. Recently, a YY1-dependent recruitment of not only INO80 but also the BAF complex was shown in embryonic stem cells (*52*). Because BAF and INO80 share the BAF53^Arp4^/ACTB pair, our structural results offer a possible mechanistic explanation, although whether YY1 binds BAF’s A-module like it binds the INO80 A-module needs further investigation.

The way YY1/Pho interacts with the INO80 A-module or polycomb-associated factors (*34*) suggests that it cannot bind two complexes at the same time, which explains the partitioning and also the different roles in, e.g., cell survival (*53*). To this end, however, our structures might be useful to design point mutations that selectively perturb the YY1 interactions with either INO80 or polycomb complexes, thus helping to functionally dissect its different roles in vivo.

Previous functional evidence identified the Ino80^HSA^ domain and the Arp8 N-terminus as critical for extranucleosomal DNA sensing (*27*, *28*). Here, we provide a structural basis for this activity, showing how the A-module specifically recognizes DNA. Both the Arp8 N-terminal extension and the Ino80^HSA^ domain directly bind DNA, altogether spanning three helical turns. We observed binding of curved DNA, which is of interest in the context of distinguishing nucleosome-depleted promoter DNA elements from DNA in nucleosome-bound gene bodies. Because the A-module binds to the concave side of the curved DNA, like the histones in the nucleosome, it might help distinguish nucleosome-receptive DNA in gene bodies from more rigid DNA in NDRs. We also find that the hook element of Arp8 widens the minor groove upon DNA binding and could contribute to DNA feature readout.

In addition to the A-module, the Arp5 subunit emerges as a critical regulator of the remodeling reaction, thus identifying all actin-related proteins of the INO80 as core regulators. We consistently observe two major configurations in the helical insertion domain of Arp5 denoted grappler (*20*), which might point to rather complex functional roles in sliding or exchange reactions. While understanding the function of the parallel state and the precise role of the conformational switch needs to be addressed in future work, the cross state of the Arp5 grappler binds to the entry DNA opposing the Ino80^motor^ domain. This interaction appears to stabilize the path of entry DNA, allowing a continuous HSA helix to chemomechanically couple the extranucleosomal DNA-bound A-module to the N-lobe of the motor domain. To enable this configuration, DNA needs to be bent at or ahead of Ino80^motor^, which provides a possible additional DNA feature sensing mechanism. Placing rigid DNA at this region severely affects sliding efficiency; thus, Ino80^motor^ directly, or indirectly through the Ino80^HSA^–A-module geometry, could be responsive to DNA mechanical properties. For instance, the extended Ino80^HSA^ domain and the A-module could act like a lever arm in this regard. Such a scenario might also help rationalize the peculiar NCP recognition mode of INO80 complexes as opposed to other remodelers. In INO80, the motor is placed at SHL-6 on the entry DNA, while in others, the motor is placed within the nucleosome at SHL-2. When the motor is positioned at SHL-6, it is able to not only pump DNA into the nucleosome but also monitor DNA features at the same time. In contrast, a motor at SHL-2 might be more blind to shape features because the histones prebend DNA anyway.

A-module and Ino80^motor^ are chemomechanically coupled to the HSA/post-HSA helix. The allosteric regulation of Swi2/Snf2 motor domains by helical regulatory elements at the N-lobe is well founded (*54*, *55*). In structural studies, these elements are often not visible, and might be rather transient, or show large conformational variabilities (*25*). In our structures, we observed that switching Ino80^motor^ from *apo* to nucleotide-bound states affects the interaction of the post-HSA with the N-lobe, a feature that could be intimately linked to remodeling. Because mutations in Ino80^HSA^ severely reduce remodeling without substantially affecting the ATPase rate, it is plausible that the post-HSA might not switch the motor on or off but rather provides a critical functional connection in a remodeling step. It could couple the motor activity to productive, directional DNA translocation and reduce futile ATP hydrolysis steps without DNA translocation. If this is the case, it is unlikely that the A-module is simply a floating lever arm on extranucleosomal DNA but could undergo positional changes to help translocate DNA.

In summary, we provide a detailed mechanism for extranucleosomal DNA binding by Ino80^HSA^ and A-module and reveal how it is chemomechanically coupled to the motor of the C-module. The overall architecture reveals multiple instances of extranucleosomal curved DNA, indicating an integrative monitoring of DNA features [propeller twist (*15*)] as one way to tune INO80 sliding (Fig. 9). Future studies need to address how the INO80 complex interacts with other substrates such as hexasomes and nucleosomal arrays. This will allow us to gain further insights into the conformational spectrum of the complex, the way INO80 has “ruler” functions in the generation of nucleosomal arrays (*16*), and possibly understand the suggested histone exchange activities as well (*10*). To this end, it will be important to visualize the evolutionary highly variable N-terminal modules, which may add not only additional targeting but also negative regulatory activities (*26*). Nevertheless, our analysis provides an important step forward in the mechanistic understanding of these complex and fascinating chromatin-shaping molecular machines.

## MATERIALS AND METHODS

### Expression and purification of the INO80 complex from *C. thermophilum*

Subunits of the *Ct*INO80 complex and mutants were cloned and expressed by using the MultiBac technology. The gene coding for Ino80^718–1848^ (*Ct*Ino80^ΔN^)–2xFLAG® was cloned in pACEBac1; genes for Rvb1 and Rvb2 were cloned in pIDC; and genes coding for Arp5, Ies6, and Ies2 were cloned in the pIDK vector. Together, they were combined in one bacmid. *Ino80*^545–850^ (INO80 A-module) was also cloned in pACEBac1. *Ino80*^1–850^ (INO80ΔC) and *Ies1* were also cloned in pACEBac1. Genes coding for HMG and Iec3 were cloned in pIDC. Genes coding for ZnF and FHA were cloned in the pIDS vector. Genes coding for Ies4 and Taf14 were cloned in pACEBac1, and genes coding for Arp8, actin, and Arp4 were cloned in a pIDK vector and combined on a separate bacmid. *PirHC* (Geneva Biotech) and *Escherichia coli* XL1-Blue (Stratagene) cells were used for all recombination steps by the addition of the Cre recombinase (NEB). From each bacmid (generated in *E. coli* DH10 MultiBac cells), baculoviruses were generated in *Spodoptera frugiperda* (Sf21) insect cells (Thermo Fisher Scientific, #11497013). Each baculovirus (1:100) was transferred to 1 liter of *Trichoplusia ni* High Five cells (Invitrogen, #B85502), thereby coinfecting the cells. Cells were cultured for 60 hours at 27°C and harvested by centrifugation at 4°C.

Cells were disrupted in lysis buffer [30 mM Hepes (pH 7.8), 400 mM NaCl, 10% glycerol, 0.25 mM dithiothreitol (DTT), pepstatin A (0.28 μg/ml), phenylmethylsulfonyl fluoride (PMSF; 0.17 mg/ml), benzamidine (0.33 mg/ml), and 2 mM MgCl_2_] for complex purification and gently sonified for 2 min (duty cycle, 50% and output control, 5). Raw lysate was cleared by centrifugation at 20,500*g* and 4°C for 30 min. Supernatant was incubated with 2 ml of ANTI-FLAG® M2 Affinity Gel (Sigma-Aldrich) for 1 hour and washed with 50 ml of lysis buffer and 75 ml of wash buffer [30 mM Hepes (pH 7.8), 150 mM NaCl, 5% glycerol, and 0.25 mM DTT]. The protein was eluted from the matrix by incubation with 4.5 ml of wash buffer [supplemented with FLAG® peptide (0.2 mg/ml)] in three incubation steps of 20 min each.

The elution fractions were loaded onto a Mono Q 5/50 GL column (GE Healthcare) and eluted by an increasing salt gradient (200 mM NaCl to 1 M NaCl), resulting in highly pure INO80. The Ino80^ΔN^ and A-module mutants were generated by site-directed mutagenesis and expressed and purified as described above (table S4).

### Expression and purification of the INO80 complex from *S. cerevisiae*

The coding sequences of the INO80 subunits were cloned into pFBDM vectors. One vector contained the C-terminally 2xFLAG-tagged Ino80 coding sequence *Ino80*^1–598^ (INO80ΔC), and a second vector contained the remaining coding sequences for the subunits of the A- and N-modules (actin, Arp4, Arp8, Taf14, Ies4, Ies1, Ies3, Ies5, and Nhp10; table S4). Bacmids were generated using *E. coli* DH10 MultiBac cells. Baculoviruses were generated in *S. frugiperda* (Sf21) insect cells (Thermo Fisher Scientific, #11497013). *T. ni* High Five cells (Invitrogen, #B85502) were coinfected with two viruses (1:100, v/v) and cultured for 60 hours at 27°C. The cells were harvested by centrifugation at 4°C.

For purification of the INO80 complexes, cells were resuspended in lysis buffer [50 mM tris (pH 7.9), 500 mM NaCl, 10% glycerol, 0.25 mM DTT, pepstatin A (0.28 μg/ml), PMSF (0.17 mg/ml), and benzamidine (0.33 mg/ml)] and disrupted by sonication (4 × 1 min; duty cycle, 50%; and output control, 5). The lysate was cleared by centrifugation at 20,500*g* and 4°C for 40 min. Supernatant was incubated with 3 ml of ANTI-FLAG® M2 Affinity Gel (Sigma-Aldrich) for 1 hour and washed with 50 ml of wash 1 buffer [25 mM Hepes (pH 8), 500 mM KCl, 10% glycerol, 0.05% IGEPAL CA630, 4 mM MgCl_2_, and 0.25 mM DTT], 50 ml of wash 2 buffer [25 mM Hepes (pH 8), 200 mM KCl, 10% glycerol, 0.05% IGEPAL CA630, 4 mM MgCl_2_, and 0.25 mM DTT], and 10 ml of buffer A [25 mM Hepes (pH 8), 150 mM KCl, 2 mM MgCl_2_, and 0.25 mM DTT]. The protein was eluted from the matrix by incubation with 4.5 ml of buffer A [supplemented with FLAG® peptide (0.2 mg/ml)] in four incubation steps of 15 min each.

The elution fractions were loaded onto a Mono Q 5/50 GL column (GE Healthcare) and eluted by a linear salt gradient (150 mM KCl to 1 M KCl), resulting in highly pure INO80. The A-module was generated by site-directed mutagenesis and expressed and purified as described above (table S4).

### Expression, purification, and grid preparation of *Hs*A-module + YY1

Human A-module (*ACTR8*, *ACTB*, *ACTL6A*, and *YY1*) open reading frames (ORFs) were ordered and optimized for insect cell expression at GeneArt (Thermo Fisher Scientific) and assembled on a single pBIG1ab vector using the biGBac cloning system. The 2xFLAG®-tagged *INO80*^HSA^ (*INO80*^267–487^-2xFLAG®) was cloned separately on a pBIG1a vector. After virus generation in Sf21 cells (*S. frugiperda,* Thermo Fisher Scientific, #11497013), the complexes were recombinantly expressed in High Five insect cells (*T. ni*; Invitrogen, #B85502) by adding the two viruses at 1:150 (volume virus:medium) to 3 liters of insect cell culture. The cells were incubated for 60 hours at 27°C and harvested by centrifugation at 4°C. For lysis, the pellet was resuspended in lysis buffer [20 mM Hepes (pH 8.0), 500 mM NaCl, 0.25 mM DTT, and 1× protease inhibitor (cOmplete, Roche)] and gently sonicated three times for 1.5 min. The lysate was incubated with ANTI-FLAG® M2 affinity gel (Sigma-Aldrich) for 1.5 hours and submitted to a gravity flow column. First, the agarose beads were washed with 10 column volumes (CV) lysis buffer [20 mM Hepes (pH 8.0), 500 mM NaCl, and 0.25 mM DTT] followed by 20 CV wash buffer [20 mM Hepes (pH 8.0), 150 mM NaCl, and 0.25 mM DTT]. Next, the protein complex was eluted three times by incubation with 1 CV wash buffer supplemented with 1xFLAG® peptide (0.4 mg/ml) for 15 min each. The elution fractions were applied onto a Capto HiRes Q 5/50 column (Cytiva), and the protein complex was separated via a salt gradient (100 mM NaCl to 1000 mM NaCl) using buffer A [20 mM Hepes (pH 8.0), 100 mM NaCl, 20 mM ZnCl_2_, 4 mM MgCl_2_, and 0.25 mM DTT] and buffer B [20 mM Hepes (pH 8.0), 100 mM NaCl, 20 mM ZnCl_2_, 4 mM MgCl_2_, and 0.25 mM DTT]. Protein target peak fractions were concentrated to 2 mg/ml in centrifugal filters (Centricon; 70-kDa cutoff, Millipore) and flash-frozen in liquid nitrogen.

For cryo-EM analysis, the purified A-module + YY1 was vitrified on glow-discharged R2/1 copper mesh 200 grids (Quantifoil). β-Octyl glucoside (Roth, Germany) was added at a final concentration of 0.05%. The sample (4.5 μl) was preincubated on the grid for 20 s before blotting.

### Purification of nucleosomes

Canonical human histones (HistoneSource) were resuspended in an unfolding buffer [7 M guanidinium chloride, 20 mM tris (pH 7.5), and 1 mM DTT], respectively, under rotation for 30 min at room temperature. Histones were mixed in 1.1-fold excess of H2A and H2B and dialyzed against 4× 1-liter refolding buffer [20 mM tris (pH 7.5), 2 M NaCl, 1 mM DTT, and 0.5 mM EDTA (pH 8)] for 16 hours at 4°C. Histone octamers were purified by size exclusion chromatography using a Superdex 200 16/60 column (GE Healthcare). After concentrating to 4 mg/ml in centrifugal filters (Centricon; 10-kDa cutoff; Millipore), histone octamers were stored in 50% glycerol at −20°C.

Widom 601 DNA with 80-bp extranucleosomal DNA in the 0N80 orientation for reconstituting nucleosomes was used as a DNA template (table S5). DNA was amplified by polymerase chain reaction (PCR), followed by purification using anion exchange chromatography, and the DNA was concentrated in vacuum after the DNA was dialyzed to H_2_O overnight. DNA was mixed at a 1.1-fold excess with the histone octamer at 2 M NaCl. The NaCl concentration was decreased to 50 mM over 16 hours at 4°C. After this, nucleosomes were purified by anion exchange chromatography using a SourceQ 1-ml column, and fractions containing nucleosomes were pooled and dialyzed to 50 mM NaCl, concentrated to 1 mg/ml (Centricon; 10-kDa cutoff, Millipore), and stored at 4°C.

### Nucleosome sliding assays

0N80 nucleosomes with 5′-fluorescein–labeled extranucleosomal DNA were used for monitoring the sliding activity of *Ct*INO80^ΔN^ and mutants. Nucleosome (150 nM) was incubated with 50 nM *Ct*INO80^ΔN^ in sliding buffer [25 mM Hepes (pH 8), 60 mM KCl, 7% glycerol, bovine serum albumin (BSA; 0.10 mg/ml), 0.25 mM DTT, and 2 mM MgCl_2_] at 25°C. By the addition of 1 mM ATP, the sliding reaction was started and stopped at several time points (15, 30, 45, 60, 120, 300, 600, and 1200 s) by addition of Lambda DNA (0.2 mg/ml; NEB). Nucleosome species were separated by native polyacrylamide gel electrophoresis (PAGE) on a 3 to 12% acrylamide bis-tris gel (Invitrogen) and visualized using the Typhoon imaging system (GE Healthcare). Experiments were performed in triplicates. For gel band quantification, ImageJ was used and the fraction of remodeled band was plotted against the reaction time in percent. Data describe a saturation curve and was fitted in Prism (GraphPad) using an exponential equation.

### NADH-coupled ATPase assay

NADH (reduced form of nicotinamide adenine dinucleotide)–coupled ATPase assays were used to determine the ATPase rate of *Ct*INO80^ΔN^ and mutants. *Ct*INO80^ΔN^ (30 nM) was incubated in assay buffer [25 mM Hepes (pH 8), 50 mM KCl, 1 mM DTT, 2 mM MgCl_2_, and BSA (0.1 mg/ml)] with 0.5 mM phosphoenolpyruvate, 1 mM ATP, 0.1 mM NADH, and lactate dehydrogenase (25 U/ml) and pyruvate kinase (Sigma-Aldrich) at 25°C in a final volume of 50 μl. Decreasing NADH concentration was monitored fluorescently over 1 hour in nonbinding, black, 384-well plates (Greiner Bio-One) using 340 nm for excitation and an emission of 460 nm with Tecan Infinite M100 (Tecan). Where indicated, ATPase rate was determined in the presence of 200 nM nucleosome. Experiments were performed in triplicates. ATP turnover was calculated using maximal initial linear rates, corrected for a buffer blank.

### Affinity measurement by fluorescence anisotropy

Increasing protein concentrations of the *Ct*INO80 A-module and mutants (final concentrations for *C. thermophilum* A-module: 0, 3.125, 6.25, 12.5 25, 50, 100, and 200 nM) were prepared in assay buffer [25 mM Hepes (pH 8), 100 mM KCl, 2 mM MgCl_2_, 2% glycerol, 0.01% Triton X-100, and 1 mM DTT] and mixed with 50-bp 6-FAM–labeled DNA (table S5) in assay buffer (final concentration, 5 nM) in a 1:1 (v/v) ratio (final volume: 20 μl; Greiner Flat Bottom Black 384-well plate). The reaction was incubated for 30 min at room temperature, and the fluorescence anisotropy was subsequently measured at an excitation wavelength of 470 nm and an emission wavelength of 520 nm using a TECAN Infinite M1000 plate reader. Experiments were performed in triplicates. The background signal (no protein sample) was subtracted from each value of a dilution series, and the datasets were analyzed with Prism (GraphPad Software). Data were analyzed and fitted to a nonlinear, noncooperative 1:1 binding model [*y* = *A*_f_ − (*A*_f_ − *A*_b_) × (*x*/(*K*_d_ + *x*)), where *y* is the anisotropy, *A*_f_ is the anisotropy of free ligand, *A*_b_ is the anisotropy of bound ligand, *K*_d_ is the dissociation constant, and *x* is the receptor concentration] to calculate the apparent dissociation constants.

### Electrophoretic mobility shift assay

Electrophoretic mobility shift assays were used to monitor the interaction between INO80 and 0N80 nucleosomes. Nucleosomes were labeled at the 5′ end of their extranucleosomal DNA with fluorescein. Nucleosome (40 nM) was incubated with 80 nM INO80 in electrophoretic mobility shift assay buffer [25 mM Hepes (pH 8), 60 mM KCl, 7% glycerol, 0.25 mM DTT, and 2 mM CaCl_2_] for 30 min on ice. Samples were analyzed at 4°C by native PAGE on a 3 to 12% acrylamide bis-tris gel (Invitrogen) and visualized using the Typhoon imaging system (GE Healthcare).

### Purification and vitrification of *Ct*INO80^ΔN^:0N80 complex and INO80 A-module

*Ct*INO80^ΔN^ and 0N80 nucleosomes were mixed in a ratio of 2:1 and dialyzed to binding buffer [20 mM Hepes (pH 8), 60 mM KCl, 0.25 mM CaCl_2_, 20 μM ZnCl_2_, and 0.25 mM DTT] for 1 hour in Slide-A-Lyzer dialysis tubes (Thermo Fisher Scientific). The complex was vitrified at a concentration of 1 mg/ml on Quantifoil R2/1 grids in the presence of 0.05% octyl-β-glucoside using a Leica EM GP (Leica). The same was done for INO80ΔC and INO80 A-module with or without DNA and nucleotides. *Ct*INO80A-module with ATPγS bound was purified further and mildly cross-linked by GraFix using an SW40-Ti rotor (Beckman Coulter). The glycerol (10 to 30%) and glutaraldehyde (0 to 0.025%) co-gradient was generated using Gradient Station *ip* 153 (BioComp Instruments). The samples were fractionated and monitored for 280/260-nm absorbance using Triax UV Flow Cell (BioComp Instruments). The fractions were visually inspected and selected by uranyl acetate (2%) negative staining.

*S. cerevisiae* N–A-module and DNA (58 bp; table S5) were mixed in an equimolar ratio (1.5 μM each) in cryo-EM buffer [20 mM Hepes (pH 8), 60 mM KCl, 2 mM MgCl_2_, and 1 mM DTT]. The respective nucleotide was added (final concentration: 1 mM), and the sample was incubated on ice for 10 min. Octyl-β-glucoside was added (0.045%), and 4.5 μl was applied onto a glow discharged Quantifoil R2/1 Cu200 grid. The sample was vitrified in liquid ethane using an EM GP plunge freezer (Leica; 10°C and 90% humidity).

### Data collection

Movies of *Ct*INO80^ΔN^-nucleosome or A-module particles embedded in vitreous solution were collected at liquid nitrogen temperature using a Titan Krios G3 transmission electron microscope (Thermo Fisher Scientific) equipped with a K2 Summit direct electron detector (Gatan) and BioQuantum LS Imaging Filter (Gatan). The movies were recorded in counting mode using EPU acquisition software (Thermo Fisher Scientific) at ×130,000 magnification with a pixel size of 1.059 Å/pixel and nominal defocus range of 1.1 to 2.9 μm. The total electron dosage of each movie was ~40 to 46 *e*/Å^2^, fractionated into 40 movie frames with an exposure time of 250 ms/frame.

### Cryo-EM data processing of *S. cerevisiae* A-modules

The movie frames were motion-corrected using MotionCor2 (*56*). All subsequent cryo-EM data processing steps were carried out using cryoSPARC v3.3.1 (*57*) or Relion-3.0 (*58*), and the resolutions reported here are calculated on the basis of the gold-standard Fourier shell correlation criterion (FSC = 0.143).

For the *S. cerevisiae* A-module bound to ATP (fig. S10, A and C), the contrast transfer function (CTF) parameters of the dataset (4543 micrographs) were determined using patch CTF estimation (multi). The exact processing scheme and data collection and refinement statistics are summarized in table S1. Initial particle picking was done on 2048 micrographs using Blob picker. The particles were subjected to 2D classification and ab initio reconstruction, and classes with clearly defined features were selected. The selected particles were used as input for a Topaz train job on 4543 micrographs. After three rounds of Topaz, 1,028,485 particles were extracted with a box size of 256 pixels and a pixel size of 1.059 Å. The particles were subjected to multiple rounds of 2D classification and heterogeneous refinement. The class that showed the most defined features was selected (327,293 particles) and used for further refinement. The final resolution of the ATP-bound A-module reconstruction after nonuniform refinement was 3.3 Å. To identify a subset of DNA-bound particles, particles were reextracted in Relion (fig. S10B) and subjected to three rounds of 3D classification. The A-module bound to ATP and DNA was reconstructed from 69,226 particles, and the final resolution after 3D refinement was 7.5 Å.

For the *S. cerevisiae* A-module bound to ADP (fig. S11, A and B), the CTF parameters of the dataset (5550 micrographs) were determined using CTFFIND4.1. All subsequent cryo-EM data processing steps were carried out using Relion-3.0 (*58*). Data collection and refinement statistics are summarized in table S1. A total of 2,264,013 particles were picked using Autopicking, and particles were extracted with a box size of 256 pixels and a pixel size of 1.059 Å. 3D classification with five classes was performed using the A-module bound to ATPγS (filtered to 40 Å) as reference. After another round of 3D classification, 970,407 particles were selected and used for further refinement. The final resolution of the ADP-bound A-module reconstruction after postprocessing was 3.2 Å.

### Cryo-EM data processing of *S. cerevisiae* A-module in the ATPγS state

Beam-induced motions of particles were corrected using MotionCor2 (Relion-3.0) in 5 × 5 patches per frame (*56*, *58*). CTF parameters were estimated from sums of three movie frames using CTFFIND4.1 (*59*). The particles were automatically picked ab initio and qualitatively filtered using WARP (fig. S12, A and B) (*60*). The particles were boxed and extracted from the micrographs in Relion with the particle coordinates exported from WARP using the PyEM scripts developed by D. Asarnow (https://github.com/asarnow/pyem). The initial 3D reconstructions were carried out ab initio using *cis*TEM (fig. S12C). Iterative rounds of 3D classifications were carried out using Relion (*58*). The initial 3D refinements were carried out in Relion-3 using the ab initio 3D reference generated in *cis*TEM (*61*). The final resolution of the ATPγS-bound A-module reconstruction after postprocessing was 3.2 Å. The exact processing scheme is depicted in fig. S12 (A to D). Data collection and refinement statistics are summarized in table S1.

### Cryo-EM data processing of *C. thermophilum* A-modules and INO80^ΔN^

Beam-induced motions of particles were corrected using MotionCor2 (Relion-3.0) in 5 × 5 patches per frame (*56*, *58*). CTF parameters were estimated from sums of three movie frames using CTFFIND4.1. The particles were automatically picked ab initio and qualitatively filtered using WARP (*60*). The particles were boxed and extracted from the micrographs in Relion with the particle coordinates exported from WARP using the PyEM scripts developed by D. Asarnow (https://github.com/asarnow/pyem). The initial 3D reconstructions were carried out ab initio using *cis*TEM. Iterative rounds of 3D classifications were carried out using Relion-3 to remove unbound nucleosomes and separate subtly different C- and A-module conformations. The initial 3D refinements were carried out in Relion-3 using the ab initio 3D reference generated in *cis*TEM (*61*). The exact processing schemes are depicted in figs. S13 to S15. Data collection and refinement statistics are summarized in table S1.

### Cryo-EM data processing of *C. thermophilum* INO80 C-module and nucleosome (ADP∙BeF*_x_*)

The movie frames were motion-corrected using MotionCor2 (*56*). All subsequent processing steps were performed in cryoSPARC v3.2.0 (*57*), and the resolutions reported here are calculated on the basis of the gold-standard Fourier shell correlation criterion (FSC = 0.143). The CTF parameters of the dataset (6064 micrographs) were determined using patch CTF estimation (multi) in cryoSPARC (v3.2.0). The exact processing scheme is depicted in fig. S16A. Data collection and refinement statistics are summarized in table S1.

Initial particle picking was done using Blob picker. Particles were subjected to 2D classification and ab initio reconstruction. Classes with clearly defined features were selected and used as input for a Topaz train job on all micrographs, followed by particle extraction and 2D classification. After three rounds of Topaz, 304,000 particles were extracted with a box size of 360 pixels and a pixel size of 1.059 Å. After selecting 2D classes with clearly defined features, one round of ab initio reconstruction with three classes was performed. Classes with the most defined features were selected and subjected to heterogeneous refinement with two classes. The ab initio reconstructions were used as input volumes for the heterogeneous refinement job. Both classes were selected for further refinement. The final resolution of the reconstruction after nonuniform refinement were 3.5 Å for parallel grappler and 3.8 Å for the cross grappler (fig. S16C).

For a detailed analysis of Ino80^motor^, beam-induced motions of particles were corrected using MotionCor2 (Relion-3.0) in 5 × 5 patches per frame (*56*, *58*). CTF parameters were estimated from sums of three movie frames using CTFFIND4.1. The exact processing scheme is depicted in fig. S16B. A total of 13,704,000 particles were automatically picked ab initio*.* In total, 1,242,248 manually picked particles were extracted with a box size of 360 pixels and a pixel size of 1.059 Å. Iterative rounds of 3D classifications and 3D refinement were carried out. After the last round of 3D classification, 137,900 particles were selected and used for further refinement. The final resolution of Ino80^motor^ after postprocessing was 3.6 Å (fig. S16D). Data collection and refinement statistics are summarized in table S1.

### Cryo-EM data processing of *H. sapiens* INO80 A-module

The movie frames were motion-corrected using MotionCor2 (*56*). All subsequent processing steps were performed in cryoSPARC v3.2.0 (*57*), and the resolutions reported here are calculated on the basis of the gold-standard Fourier shell correlation criterion (FSC = 0.143). The exact processing scheme is depicted in fig. S17A. Data collection and refinement statistics are summarized in table S1.

Initial particle picking was done using Blob picker. Particles were subjected to 2D classification. Classes with clearly defined features were selected and used as input for a Topaz train job on all micrographs, followed by particle extraction and 2D classification. After three rounds of Topaz, 15,000 particles were extracted with a box size of 256 pixels and a pixel size of 1.059 Å. After selecting 2D classes with clearly defined features, one round of ab initio reconstruction with one class was performed. The final resolution of the reconstruction after nonuniform refinement was 7.5 Å (fig. S17B).

### Model building and refinement

A-modules for *S. cerevisiae* and *C. thermophilum* were built with the crystal structure of the *S. cerevisiae* Arp8 module (*27*) as initial template. For each dataset, the model was manually placed into the unsharpened cryo-EM map followed by rigid-body refinement with ChimeraX (*62*). The model was then initially modified and corrected with COOT (*63*) against the sharpened cryo-EM map. Reciprocal space refinement using jelly-body restraints was done with SERVALCAT (*64*) against maximum-likelihood weighted structure factors calculated from cryo-EM half-maps. Further model building was done with COOT against the maximum-likelihood estimate of the expected true map calculated with SERVALCAT. Final model corrections were done with ISOLDE (*65*) against the same SERVALCAT map, followed by a final round of reciprocal space refinement using jelly-body restraints with SERVALCAT.

The structures of *C. thermophilum* INO80 C-module and *S. cerevisiae* A-module [Protein Data Bank (PDB): 6FML and 5NBN] were docked into the cryo-EM densities using MOLREP (CCP-EM) (*66*) and manually mutated and built in previously unobserved regions using COOT (*63*). All protein models were real space–refined using PHENIX (*67*) and evaluated using COOT and the MolProbity server. The reconstruction cryo-EM maps were deposited in the Electron Microscopy Databank (EMDB), and the coordinates of the atomic models were deposited in the PDB. The figures were generated using ChimeraX (*62*).

### Yeast manipulation and methods

All strains used (listed in table S3) were isogenic to W303 and were constructed via a diploid derivative of YCL076 (*39*). Briefly, knockouts of *INO80* and *ARP8* were generated in a diploid strain using a PCR-based strategy and confirmed by PCR with locus-specific primers (*68*, *69*). Mutant or WT alleles of either gene were cloned into the YIplac211 vector with endogenous promoter sequences and mutations as indicated and were then integrated at the *URA3* locus. Single-copy integration was tested by PCR. Diploid strains were subsequently sporulated, and tetrads were dissected for tetrad analysis and to obtain haploid knockout and point mutant strains for phenotypic analysis.

For growth assays, cells were grown overnight and adjusted to 0.5 OD_600_ (optical density at 600 nm) units and fivefold serial dilutions were spotted on YPD plates (1% yeast extract, 2% peptone, and 2% glucose), YP + Gal plates (2% galactose), or SD-inositol plates [yeast nitrogen base (6.9 g/liter) without inositol, Formedium CYN37CFG, supplemented with adenine (40 mg/liter), uracil (40 mg/liter), tryptophan (40 mg/liter), histidine (40 mg/liter), leucine (80 mg/liter), and 2% glucose]. Cells were then grown at 30°C for 2 to 5 days unless indicated otherwise. For anaerobic growth conditions, plates were incubated in an anaerobic chamber.

Protein expression levels were determined by total protein extraction from a logarithmic culture using alkaline lysis followed by trichloroacetic acid precipitation as described (*68*). Proteins were separated by SDS-PAGE and analyzed by western blotting using anti-FLAG® (Sigma-Aldrich, A8592) and Pgk1 (Invitrogen, #459250) antibodies.

### Recombination assay

To measure the efficiency of homologous recombination, a quantitative PCR (qPCR)–based gene conversion assay was used as described previously (*39*). Briefly, yeast strains were deficient of endogenous HO endonuclease cleavage sites and engineered with galactose-inducible HO endonuclease, a single HO cut site at ChrIV 491 kb, and a recombination donor sequence at ChrIV 795 kb with a mutated HO cut site and an additional unique 23-bp sequence to allow qPCR analysis. Yeast cells of the indicated genotypes were grown to logarithmic phase in YP + 2% raffinose medium, and HO endonuclease expression was induced by addition of 2% galactose. Aliquots equivalent to one OD_600_ unit were harvested at the indicated time points, and genomic DNA was isolated using the Epicentre MasterPure Yeast DNA Purification Kit (MPY80200). qPCR was performed on a LightCycler 480 instrument (Roche) using LightCycler 480 SYBR Green I Master (Roche 04707516001) with primers designed to detect the completed recombination product (5′-CATACTGTCTCACTCGCTTGGA-3′ and 5′-TTGTTTGCCATTTCGTCAGCTAG-3′). Data were normalized to an unrelated control locus (*MDV1* locus, primers 5′-GCGTGCCTGGTCACAGGTTCATACGAC-3′ and 5′-TCATACGGCCCAAATATTTACGTCCC-3′) and plotted using the GraphPad Prism software as the relative amount of recombination product over time (where 100% recombination = 1). Notably, yeast growth on YP + Gal plates in spot dilution provided a qualitative readout for homologous recombination efficiency as well.

### Protein cross-linking

Snap-frozen stock solutions of *H. sapiens* INO80 complex [20 mM Hepes/NaOH (pH 8.0), 200 mM NaCl, 0.5 mM CaCl_2_, 20 μM ZnCl_2_, and 0.5 mM DTT] and 0N80 nucleosome [20 mM Hepes/NaOH (pH 7.5), 50 mM NaCl, 0.5 mM DTT, and 10% glycerol] were thawed on ice and mixed in equimolar amounts in reconstitution buffer [20 mM Hepes/NaOH (pH 7.9), 60 mM KCl, 0.5 mM CaCl_2_, 20 μM ZnCl_2_, and freshly added 0.5 mM DTT]. The INO80-nucleosome complex mixture was incubated on ice for 30 min to allow for reconstitution. Afterward, 4 μl of freshly prepared BS3 cross-linker stock solution (2 μg/μl in reconstitution buffer; Thermo Fisher Scientific) was added to the reconstituted complex. The complex was cross-linked at 4°C for 2 hours. After that, the reaction was quenched by adding 4 μl of 2 M ammonium bicarbonate, followed by incubation at 4°C for 30 min. Thereafter, half of the cross-linked product was processed by in-gel digestion, and the other half was processed by ethanol precipitation and in-solution digestion.

### In-gel digestion

One half of the cross-linked product was mixed with LDS sample buffer, separated in a 4 to 12% NuPAGE bis-tris gel, and stained with Coomassie blue (Colloidal Blue Staining Kit; Thermo Fisher Scientific). The highlighted area of the gel (fig. S4C) was excised and cut into small gel cubes, followed by destaining in 50% ethanol/50 mM ammonium bicarbonate. The proteins were then reduced in 10 mM DTT at 56°C and alkylated by 50 mM iodoacetamide in the dark at room temperature. Afterward, proteins were digested by trypsin (1 μg per sample) in 50 mM ammonium bicarbonate at 37°C overnight. Following peptide extraction sequentially using extraction buffer (0.1% formic acid in 30% acetonitrile) and 100% acetonitrile, the sample volume was reduced in a centrifugal evaporator to remove residual acetonitrile. The peptides were then acidified with 0.1% formic acid and purified by solid-phase extraction in C18 StageTip (*70*).

### Ethanol precipitation and in-solution digestion

The other half of the cross-linked product was mixed with 1 μl of GlycoBlue coprecipitant (15 μg/μl) (Thermo Fisher Scientific), filled with reconstitution buffer to 100 μl, and then transferred to a new 2-ml Eppendorf tube. The tube was then filled with pure ethanol to a final sample volume of 2 ml and incubated at 4°C overnight. Following centrifugation at 4°C for 1 hour, the supernatant was aspirated and the protein pellet was allowed to air-dry.

The protein pellet was resolubilized in 8 M urea/50 mM ammonium bicarbonate. The proteins were reduced in 5 mM DTT for 30 min and alkylated in 15 mM iodoacetamide for 25 min. Afterward, an additional 5 mM DTT was used to quench the iodoacetamide. The proteins were first digested by 0.5 μg of Lys-C for 3 hours. After diluting the urea concentration to 2 M with 50 mM ammonium bicarbonate, 1 μg of trypsin was added to digest the proteins overnight. All procedures were carried out at room temperature of 22°C. Following acidification to 0.5% trifluoroacetic acid, the resultant peptide solution was purified by solid-phase extraction in C18 StageTip.

### Liquid chromatography tandem mass spectrometry

Cross-linked peptides were analyzed using an Orbitrap Exploris 480 mass spectrometer (Thermo Fisher Scientific) coupled to EASY-nLC 1200 UHPLC system (Thermo Fisher Scientific). Peptides were separated in an in-house packed 55-cm analytical column (inner diameter: 75 μm; ReproSil-Pur 120 C18-AQ 1.9-μm silica particles, Dr. Maisch GmbH) by online reversed-phase chromatography through a 90-min gradient of 2.4 to 33.6% acetonitrile with 0.1% formic acid at a nanoflow rate of 250 nl/min. The eluted peptides were sprayed directly by electrospray ionization into the mass spectrometer. Each sample was injected twice and measured using two different combinations of collision energies in stepped mode (*71*). Mass spectrometry measurement was conducted in data-dependent acquisition mode using a top15 method with one full scan [resolution, 60,000; scan range, 300 to 1650 mass/charge ratio (*m*/*z*); target value, 3 × 10^6^; maximum injection time, 40 ms] followed by 15 fragmentation scans via higher-energy collision dissociation (HCD; normalized collision energy in stepped mode, 25, 30, and 35% or 27, 30, and 33%; resolution, 15,000; target value, 1 × 10^5^; maximum injection time, 40 ms; isolation window, 1.4 *m*/*z*). Only precursor ions of +3 to +8 charge state were selected for fragmentation scans. In addition, precursor ions already isolated for fragmentation were dynamically excluded for 25 s.

### Mass spectrometry data analysis

Raw data files were preprocessed by MaxQuant software package (version 1.6.5.0) (*72*) as described (*73*). The peak lists (*.HCD.FTMS.sil0.apl files) were searched using xiSEARCH (version 1.7.4) (*74*) against a target-decoy database consisting of the protein sequences of the *Hs*INO80 complex and nucleosome members. The following settings were used: enzyme specificity, trypsin; allowed maximum number of missed cleavages, 3; BS3 specificity linking K, S, T, Y, and protein N-terminus; fixed modification, carbamidomethyl (C); variable modifications, oxidation (M) and mono-links for linear peptides on K, S, T, and Y with dead-ends amidated or hydrolyzed; MS1 tolerance, 6 parts per million (ppm); MS2 tolerance, 20 ppm; boosting option activated for residue pairs; residue-level false discovery rate was set at 5%.

## Supplementary Materials

### Other Supplementary Material for this manuscript includes the following:

Movies S1 to S3

Raw data file

## Notes

View/request a protocol for this paper from *Bio-protocol*.
