# Supplementary material for: Structural mechanism of extranucleosomal DNA readout by the INO80 complex
Source: Sci Adv. 2022 Dec 9;8(49):eadd3189. doi: 10.1126/sciadv.add3189 (PMC9733932; doi:10.1126/sciadv.add3189)
Supplement: Supplementary file 1 — Figs. S1 to S17 Tables S1 to S5 [file sciadv.add3189_sm.pdf]

Science Advances
AAAS

Supplementary Materials for

Structural mechanism of extranucleosomal DNA readout by the INO80 complex

Franziska Kunert *et al.*

Corresponding author: Karl-Peter Hopfner, hopfner@genzentrum.lmu.de

Sci. Adv. **8**, eadd3189 (2022)
DOI: 10.1126/sciadv.add3189

The PDF file includes:

Figs. S1 to S17
Tables S1 to S5
Legends for movies S1 to S3
Legend for raw data file

Other Supplementary Material for this manuscript includes the following:

Movies S1 to S3
Raw data file

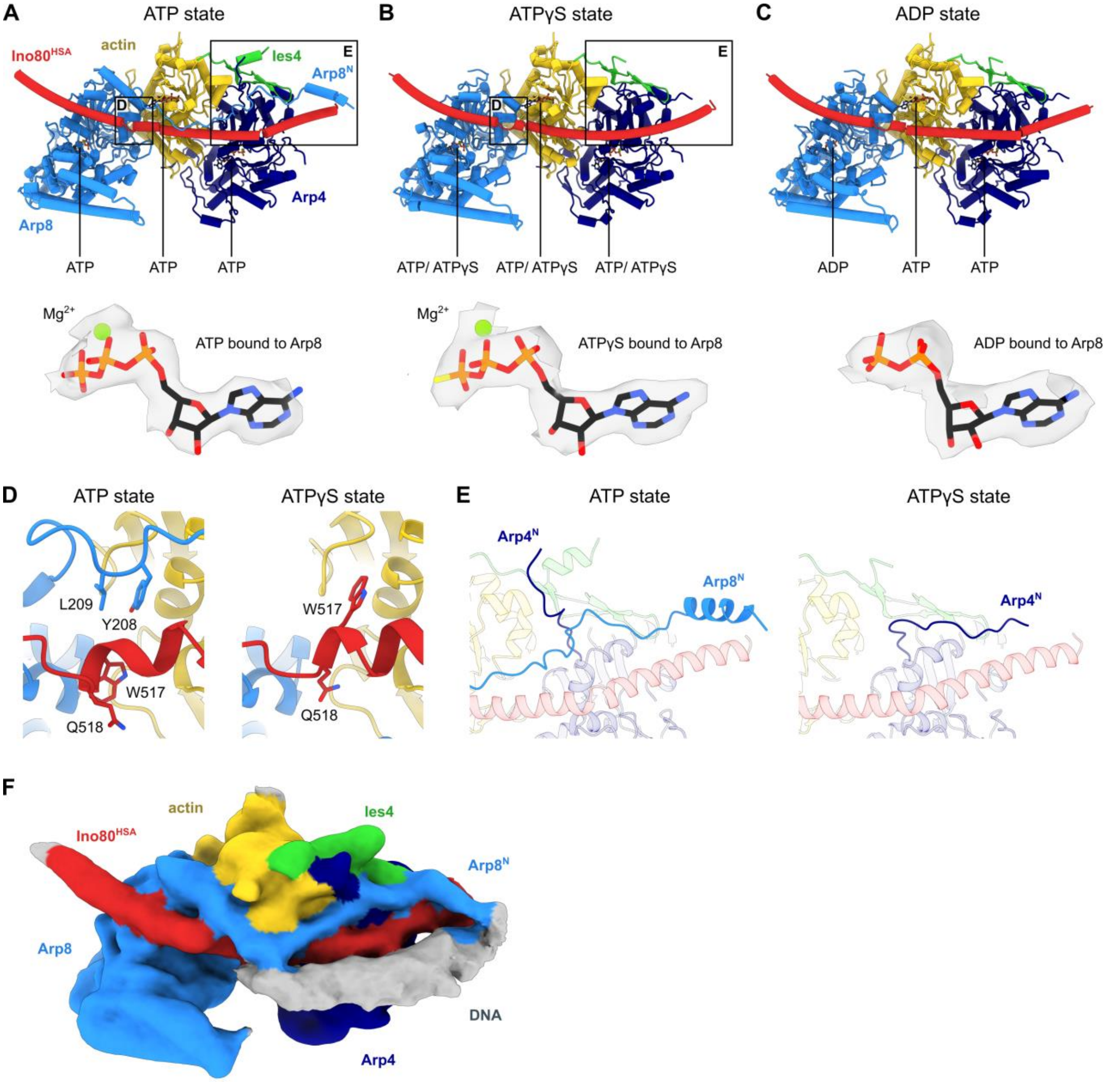

Fig. S1. The Arp8 N-terminus of the *S. cerevisiae* A-module binds DNA. (A-C) Structural model of the *S. cerevisiae (Sc)* A-module in (**A**) ATP state, (**B**) ATPγS state and (**C**) ADP state (ADP state is not a fully refined model, ATPγS state docked into ADP state reconstruction and ADP modelled into Arp8 nucleotide binding site). The protein subunits are color coded and annotated and the nucleotide states of Arp8, actin and Arp4 are indicated. (Bottom) Cryo-EM density maps of nucleotides bound to Arp8 (surface cutoff: 2 Å) *(62)* (**D**) Detailed view of the hydrophobic anchors of $Ino80^{HSA}$ (W517) and $Arp8^{N}$ (Y208) (left: ATP state, right: ATPγS state). Upon binding of $Arp8^{N}$ along the A-module, Arp8 Y208 takes the position of Ino80 W517. (**E**) Detailed view of the N-termini of Arp4 and Arp8. In the ATP state (left), $Arp8^{N}$ extends along $Ino80^{HSA}$. In the ATPγS state (right), $Arp8^{N}$ is not resolved and $Arp4^{N}$ takes the position of $Arp8^{N}$. (**F**) Cryo-EM reconstructions of A-module bound to DNA (ATP state). The protein subunits are color coded and annotated.

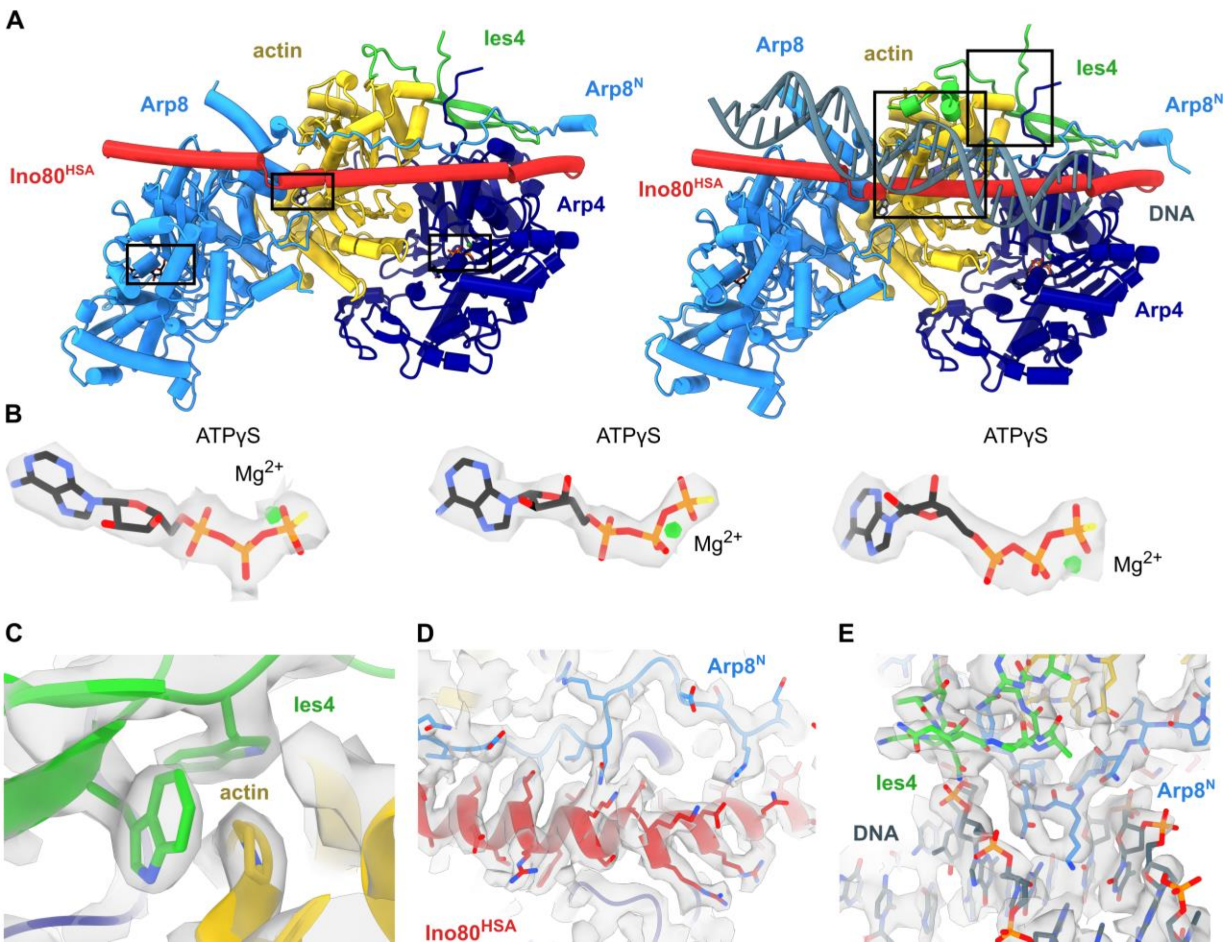

Fig S2: Examples of cryo-EM density map of the *C. thermophilum* A-module. (**A**) Structural model of *C. thermophilum (Ct)* A-module (left) without DNA or (right) with DNA bound. The protein subunits are color coded and annotated. (**B**) Cryo-EM density maps of nucleotides (ATPγS) bound to (left to right) Arp8, actin and Arp4 (surface cutoff: 2 Å) *(62)*. (**C**) Detailed view of the Ies4-actin interface in the cryo-EM density map of the *Ct*A-module. (**D**) Detailed view of the Ino80^{HSA}/Arp8^{N} interface in the cryo-EM density map of the *Ct*A-module. (**E**) Detailed view of the Ies4/Arp8^{N}-DNA interface in the cryo-EM density map of the *Ct*A-module bound to DNA.

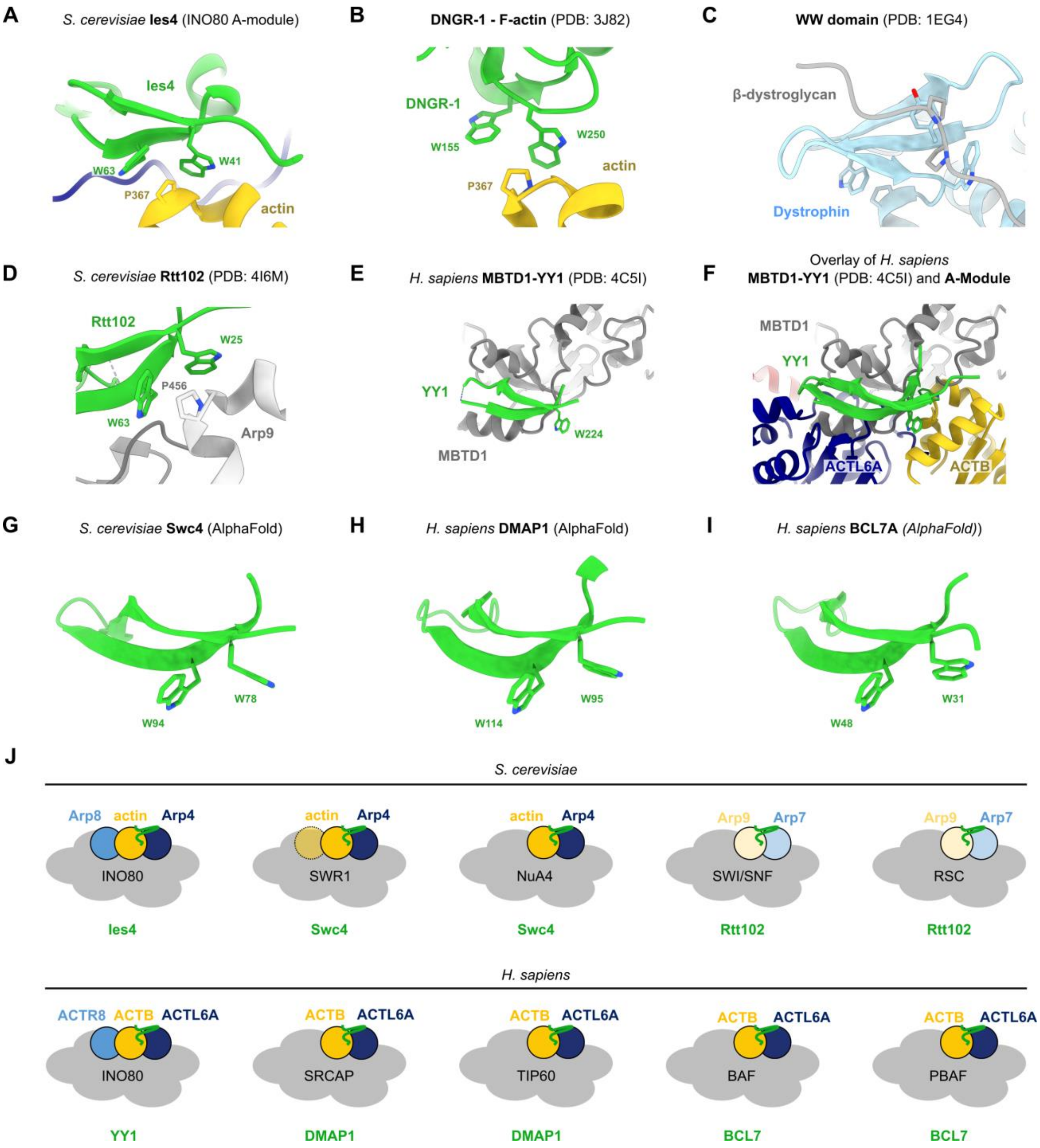

Fig. S3. The 2W-hairpin shows a conserved interaction mode with actin/ARPs. (**A**) Detailed view of the Ies4-actin interface in the *S. cerevisiae* A-module. The conserved tryptophan and proline residues are shown. (**B**) Detailed view of the DNGR-1-actin interface (PDB: 3J82). The conserved tryptophan and proline residues are shown. (**C**) Structure of the dysotrophin WW-domain in complex with a β-dystroglycan peptide (PDB: 1EG4). Conserved tryptophan and proline residues are shown. (**D**) Detailed view of the Rtt102-Arp9 interface in *S. cerevisiae* SWI/SNF A-module (PDB: 4I6M). The conserved tryptophan and proline residues are shown.

(**E**) Structure of *H. sapiens* YY1 bound to MBTD1 (PDB: 4C5I). The conserved tryptophan residue is shown. (**F**) Structural comparison of alternative YY1 binding modes. Structures of *H. sapiens* YY1 bound to MBTD1 (PDB: 4C5I) and *H. sapiens* YY1 bound to INO80 A-module are aligned onto the YY1 subunit. The conserved tryptophan and proline residues are shown. (**G**) AlphaFold-prediction of 2W-hairpin of *S. cerevisiae* Swc4. The conserved tryptophan residues are shown. (**H**) AlphaFold-prediction of 2W-hairpin of *H. sapiens* DMAP1. The conserved tryptophan residues are shown. (**I**) AlphaFold-prediction of 2W-hairpin of *H. sapiens* BCL7A. The conserved tryptophan residues are shown. (**J**) Illustration of the conservation of the Arp4-actin heterodimer in *S. cerevisiae* (Arp4-actin, Arp9-Arp7) and *H. sapiens* (ACTL6A-ACTB) INO80 and SWI/SNF family chromatin remodeling complexes. The respective 2W-hairpin containing complex subunits are indicated in green.

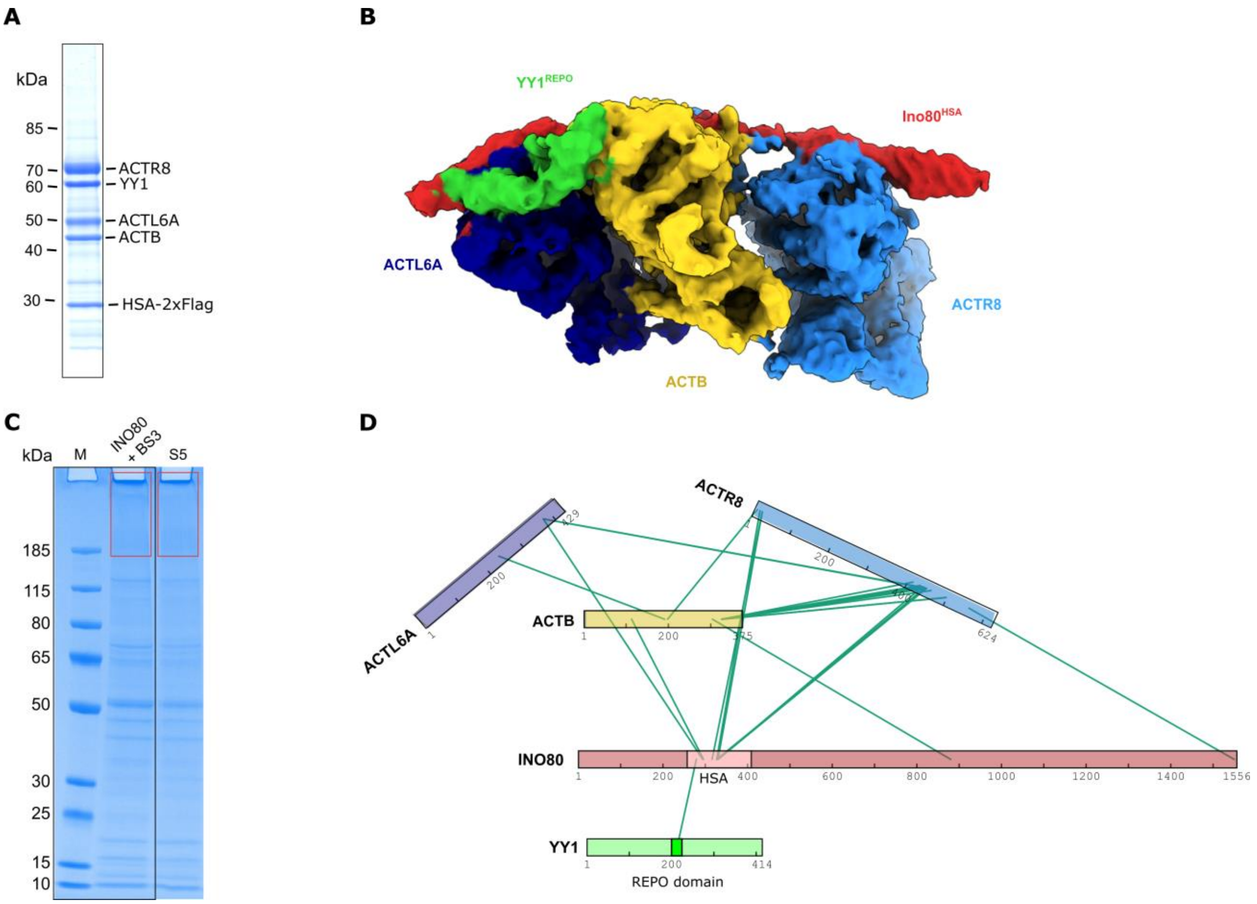

Fig. S4. YY1 interacts with the *H. sapiens* Ino80^{HSA}. (**A**) Coomassie-stained SDS-PAGE gel of the purified *H. sapiens (Hs)* A-module (**B**) Cryo-EM reconstruction of *Hs*A-module. The density is colored according to the underlying protein subunits. (**C**) Coomassie-stained SDS-PAGE gel showing the BS3 crosslinked INO80 complex. The red square comprising the high-molecular weight crosslinked species indicates the cut-out region for in gel digest used for mass spectrometry (MS) analysis. (**D**) Topological crosslink-MS scheme of *Hs*INO80 A-module subunits, showing inter-protein links (green).

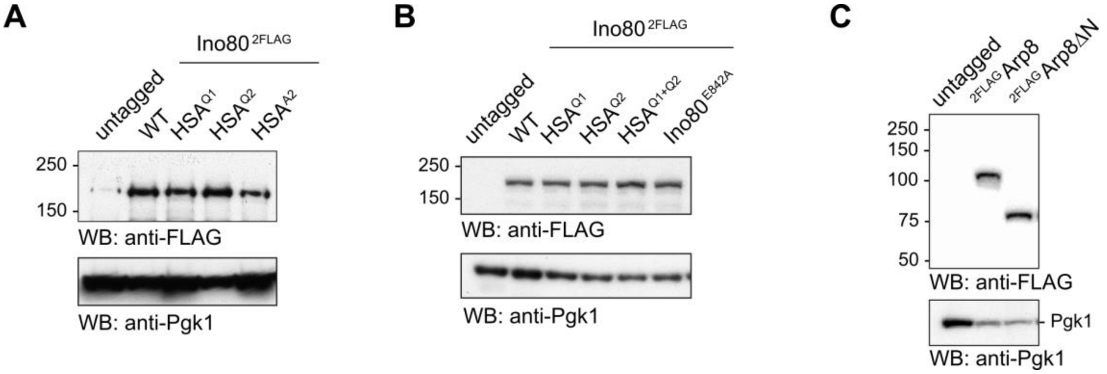

Fig. S5. HSA surface residues are critical for INO80 function in budding yeast. (**A**) Ino80 variants are expressed at levels similar to the Ino80 WT protein. Yeast cells as in Fig. 2A expressing the indicated 2FLAG-tagged Ino80 variants under the control of the endogenous *INO80* promoter were subjected to total protein analysis by western blotting using an anti-FLAG antibody. Pgk1 levels served as control. (**B**) Ino80 variants are expressed at levels similar to the Ino80 WT protein. Diploid yeast cells used for tetrad analysis in Fig. 2B expressing the indicated 2FLAG-tagged Ino80 variants under the control of the endogenous *INO80* promoter were subjected to total protein analysis by western blotting using an anti-FLAG antibody. Pgk1 levels served as control. (**C**) The Arp8ΔN variant is expressed similar to Arp8 WT levels. Yeast cells as in Fig. 2C and D expressing the indicated 2FLAG-tagged Arp8 variants under the control of the endogenous *ARP8* promoter were subjected to total protein analysis by western blotting using an anti-FLAG antibody. Pgk1 levels served as control.

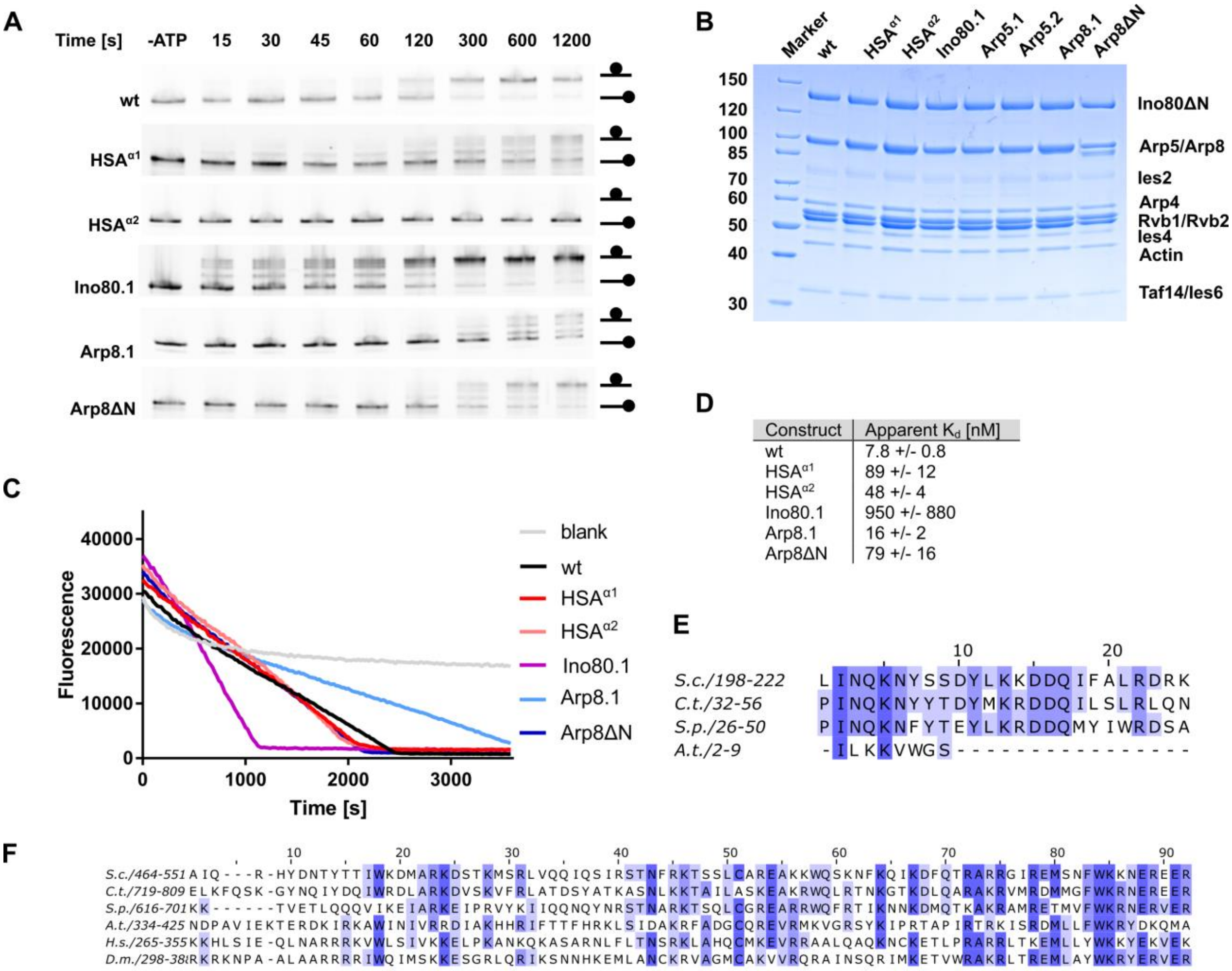

Fig. S6. ***C. thermophilum*** **INO80 mutants influence sliding and ATPase activity.** (**A**) Sliding of 0N80 nucleosomes by *Ct*INO80$^{\Delta N}$ and mutants analyzed by native PAGE. (**B**) Coomassie stained SDS-PAGE gel of *Ct*INO80$^{\Delta N}$ and mutants (**C**) Raw data of ATPase assays. ATPase rates were determined for *Ct*INO80$^{\Delta N}$ wild type (WT) and the mutants, along with nucleosome-stimulated rates. (**D**) Fluorescence anisotropy assay to monitor the binding of *Ct*A-module and mutants to a 50bp DNA. The data were fitted to a non-linear non-cooperative 1:1 binding model and the apparent K_d values were calculated. The mean +/- SEM of three independent experiments are shown. (**E**) Multiple sequence alignment *(75)* of Arp8 N-terminus (Arp8 hook). *S.c., S. cerevisiae; C.t., C. thermophilum; S.p., S. pombe; A.t., A. thaliana.* (**F**) Multiple sequence alignment *(75)* of the Ino80^{HSA}. *S.c., S. cerevisiae; C.t., C. thermophilum; S.p., S. pombe; A.t., A. thaliana; H.s., Homo sapiens; D.m., D. melanogaster.*

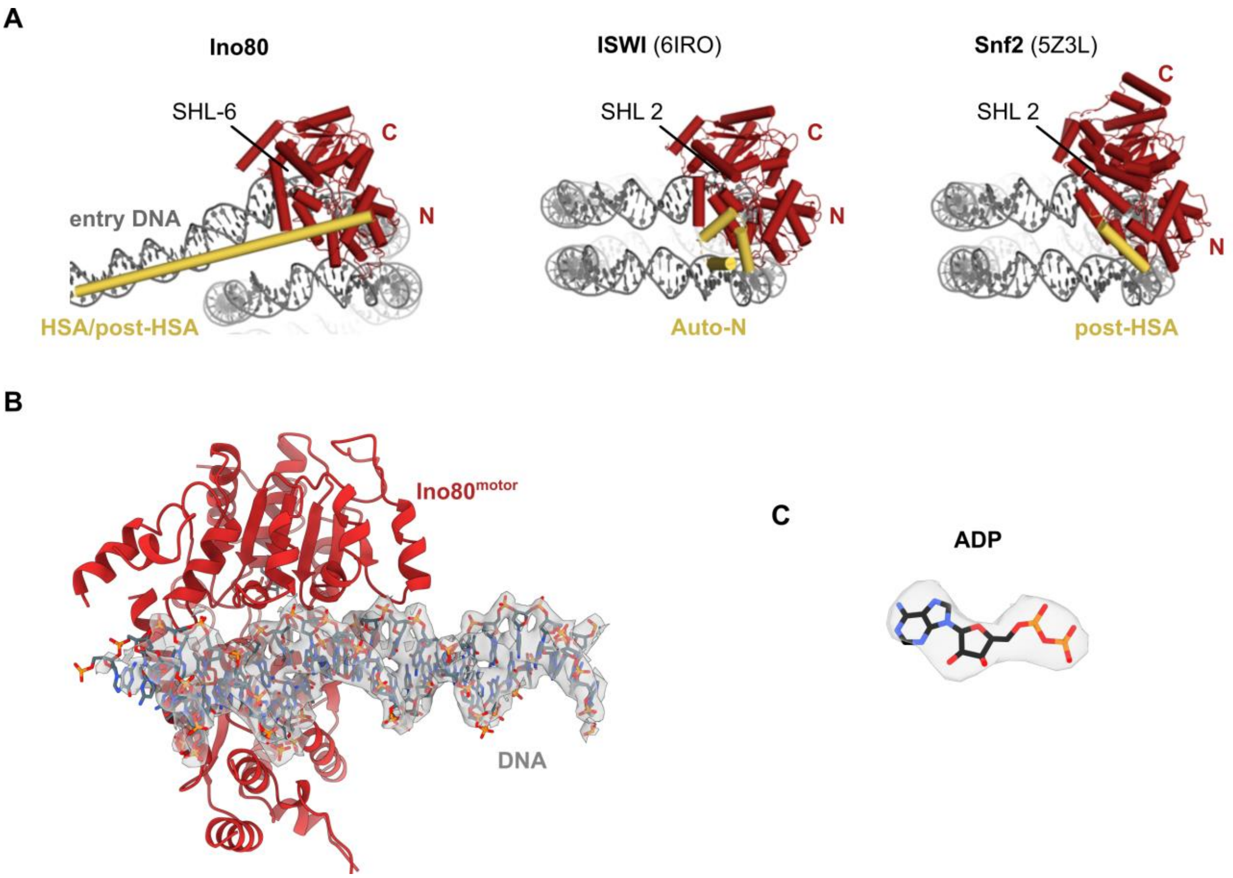

Fig. S7. Example of cryo-EM density map of the Ino80^{motor} (ADP·BeF$_x$ state). (**A**) Comparison of Ino80, ISWI and Snf2 interacting with nucleosomes and similarity of post-HSA and Auto-N. Ino80 binds the nucleosome at SHL-6 while Isw1 and Snf2 bind at SHL-2. (**B**) Detailed view of the cryo-EM density map of the Ino80^{motor}-bound DNA of the *C. thermophilum* C-module. The protein subunit is color coded and annotated (surface cutoff: 2 Å) *(62)*. (**C**) Cryo-EM density map of ADP bound to the Ino80^{motor} (surface cutoff: 2 Å) *(62)*. Note, the light BeF$_x$ moiety is not visible in the density map, in line with other studies *(19)*.

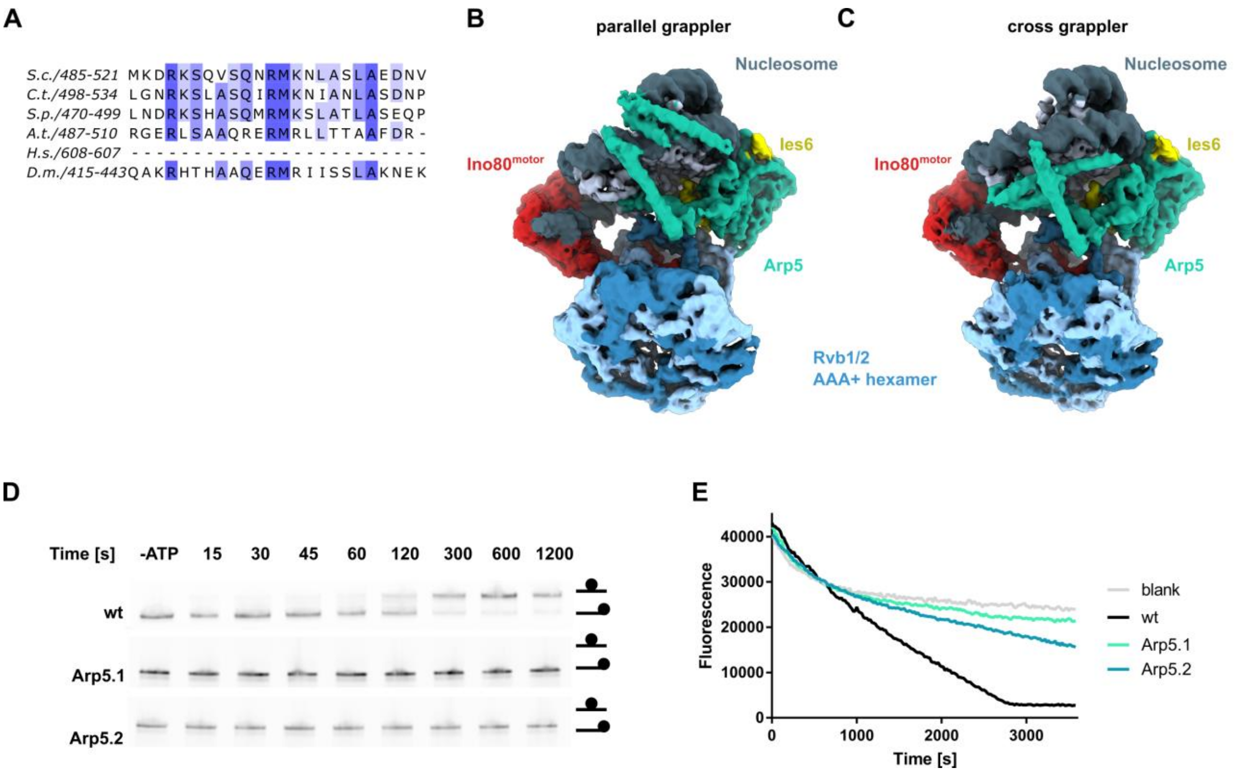

Fig. S8. Arp5 entry DNA interaction is important for *Ct*INO80$^{\Delta N}$ sliding and ATPase activity. (**A**) Multiple sequence alignment *(75)* of the grappler "foot". *S.c., S. cerevisiae; C.t., C. thermophilum; S.p., S. pombe; A.t., A. thaliana; H.s., Homo sapiens; D.m., D. melanogaster*. (**B**-**C**) Cryo-EM reconstitution of the nucleosome bound INO80 C-module complex with the Arp5 grappler in (**B**) parallel and (**C**) cross conformation. The protein subunits are color coded and annotated. (**D**) Sliding of 0N80 nucleosomes by *Ct*INO80$^{\Delta N}$ and mutants analyzed by native PAGE. (**E**) Raw data of ATPase assays. ATPase rates were determined for *Ct*INO80$^{\Delta N}$ wild type (wt) and the mutants, along with nucleosome-stimulated rates.

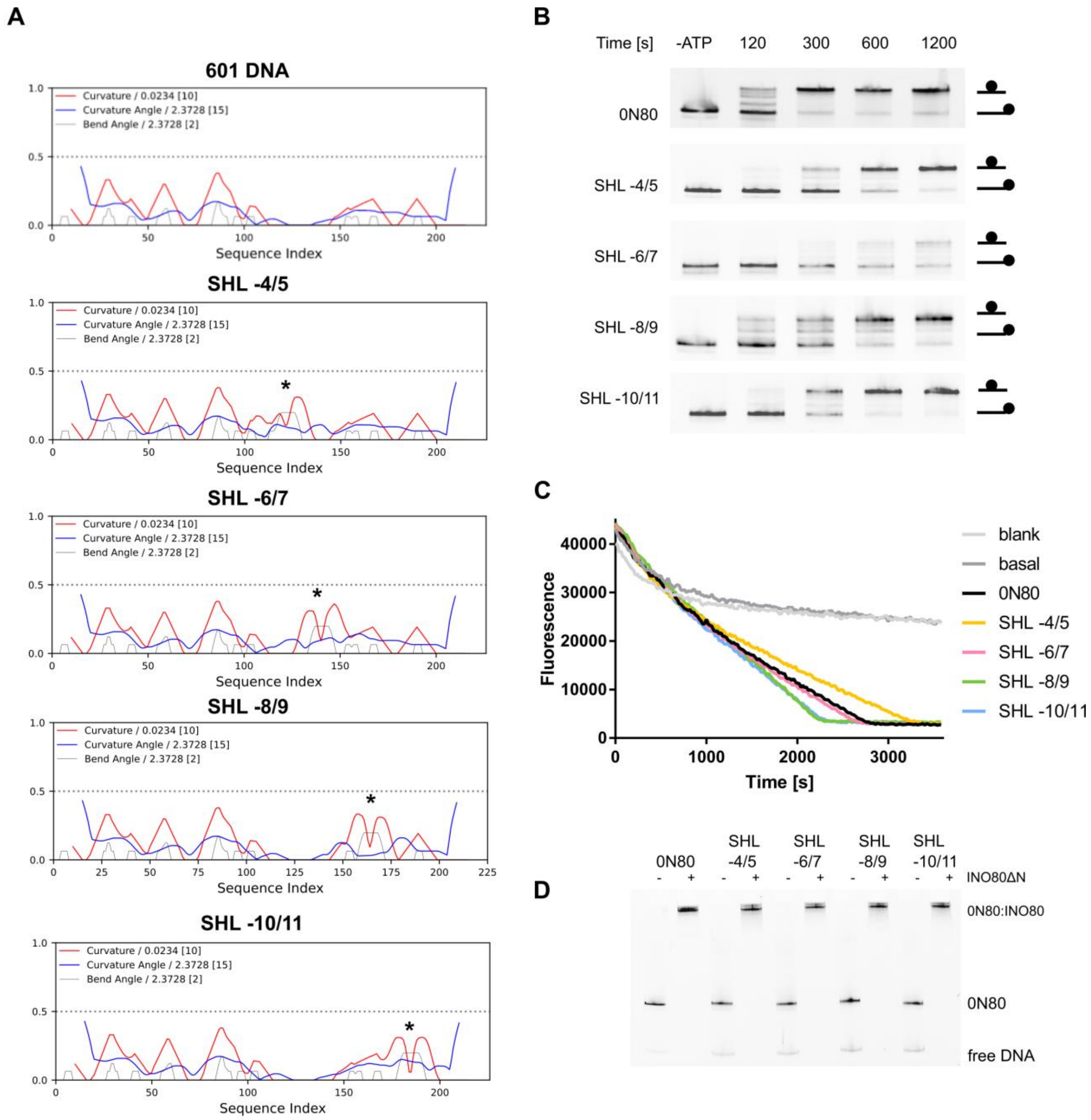

Fig. S9. Influence of A/T-rich DNA on *Ct*INO80$^{\Delta N}$ nucleosome remodeling. (**A**) DNA curvature analysis of the 601-based 0N80 DNA template sequence and sequences with replaced A/T-rich DNA cassettes (https://github.com/cgohlke/dnacurve). (**B**) Sliding of different 0N80 nucleosomes by *Ct*INO80$^{\Delta N}$ analyzed by native PAGE. (**C**) Raw data of ATPase assays in presence of different 0N80 nucleosomes. ATPase rates were determined for *Ct*INO80$^{\Delta N}$ wild type (wt) along with nucleosome-stimulated rates. (**D**) Electrophoretic mobility shift assay of different 0N80 nucleosome substrates bound to *Ct*INO80$^{\Delta N}$ analyzed by native page.

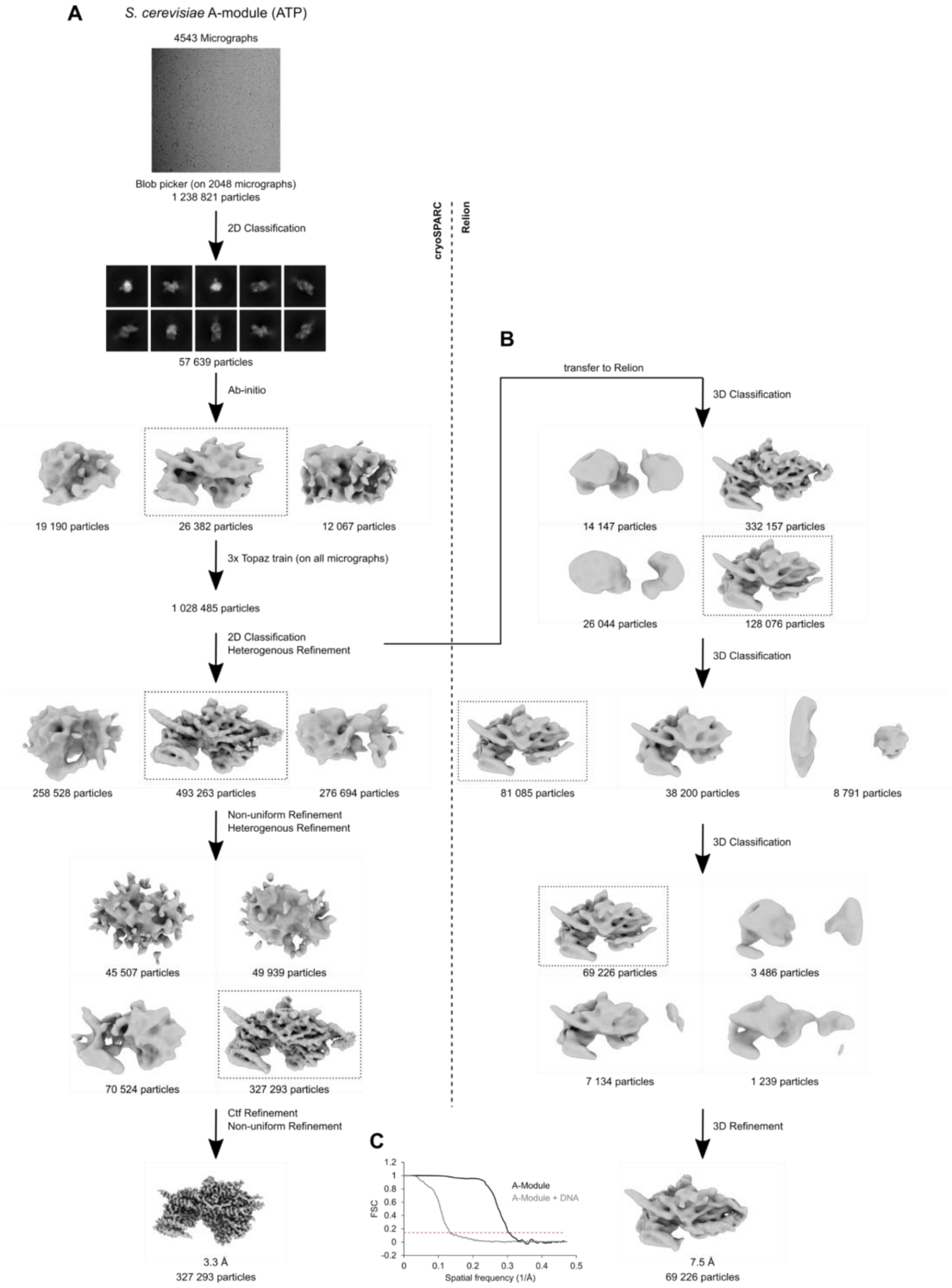

Fig. S10. Cryo-EM data analysis of *S. cerevisiae* A-module (ATP-state). (**A**) Cryo-EM data processing workflow of *S. cerevisiae* A-module using cryoSPARC v3.2.0 *(57)* and (**B**) A-module bound to DNA using cryoSPARC v3.2.0 *(57)* and Relion-3.0 *(58)*. A representative micrograph, representative 2D classes and the cryo-EM data processing workflow are shown. (**C**) Gold-standard Fourier shell correlation (FSC) curves of the final A-module and DNA bound A-module reconstructions. The red line indicates the 0.143 cutoff criterion.

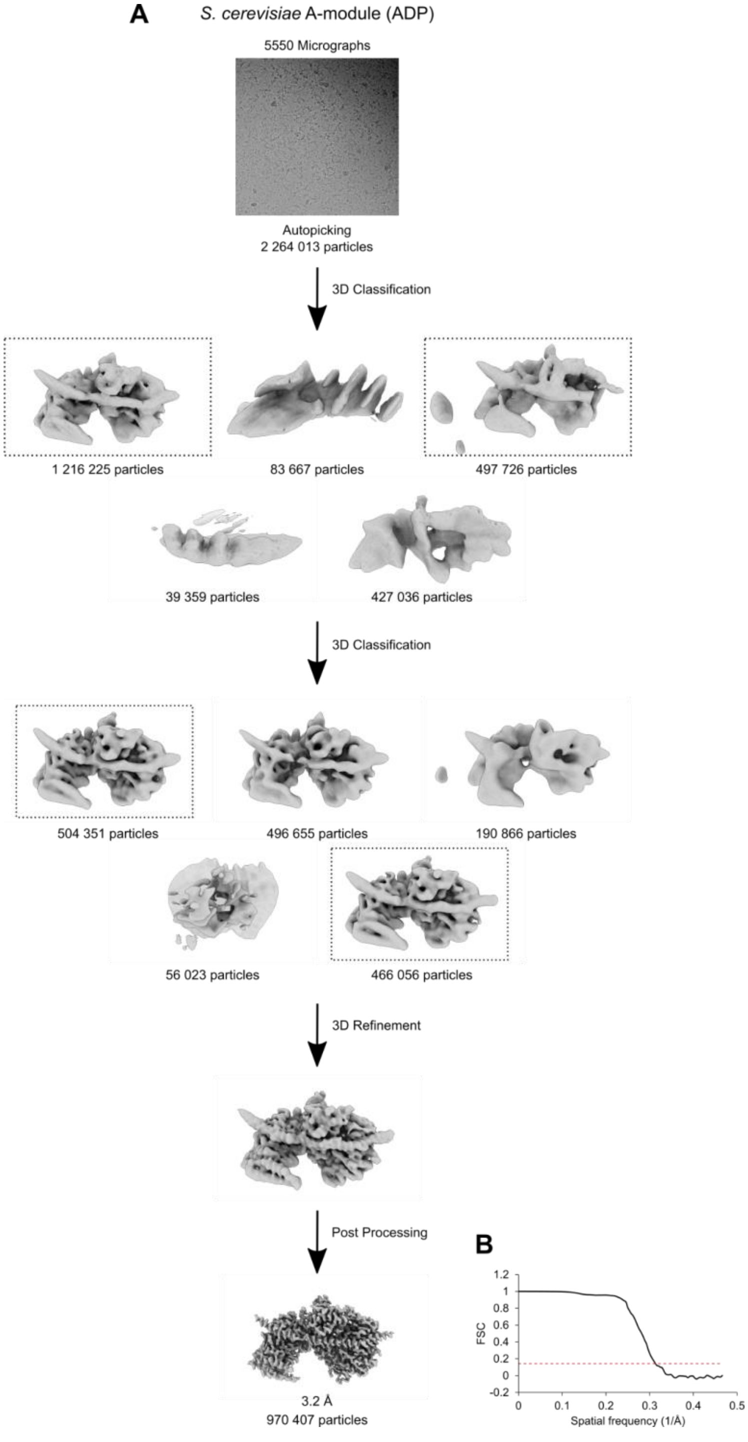

Fig. S11. Cryo-EM data analysis of *S. cerevisiae* A-module (ADP-state). (**A**) Cryo-EM data processing workflow of *S. cerevisiae* A-module using Relion-3.0 *(58)*. A representative micrograph and the cryo-EM data processing workflow are shown. (**B**) Gold-standard Fourier shell correlation (FSC) curve of the final A-module reconstruction. The red line indicates the 0.143 cutoff criterion.

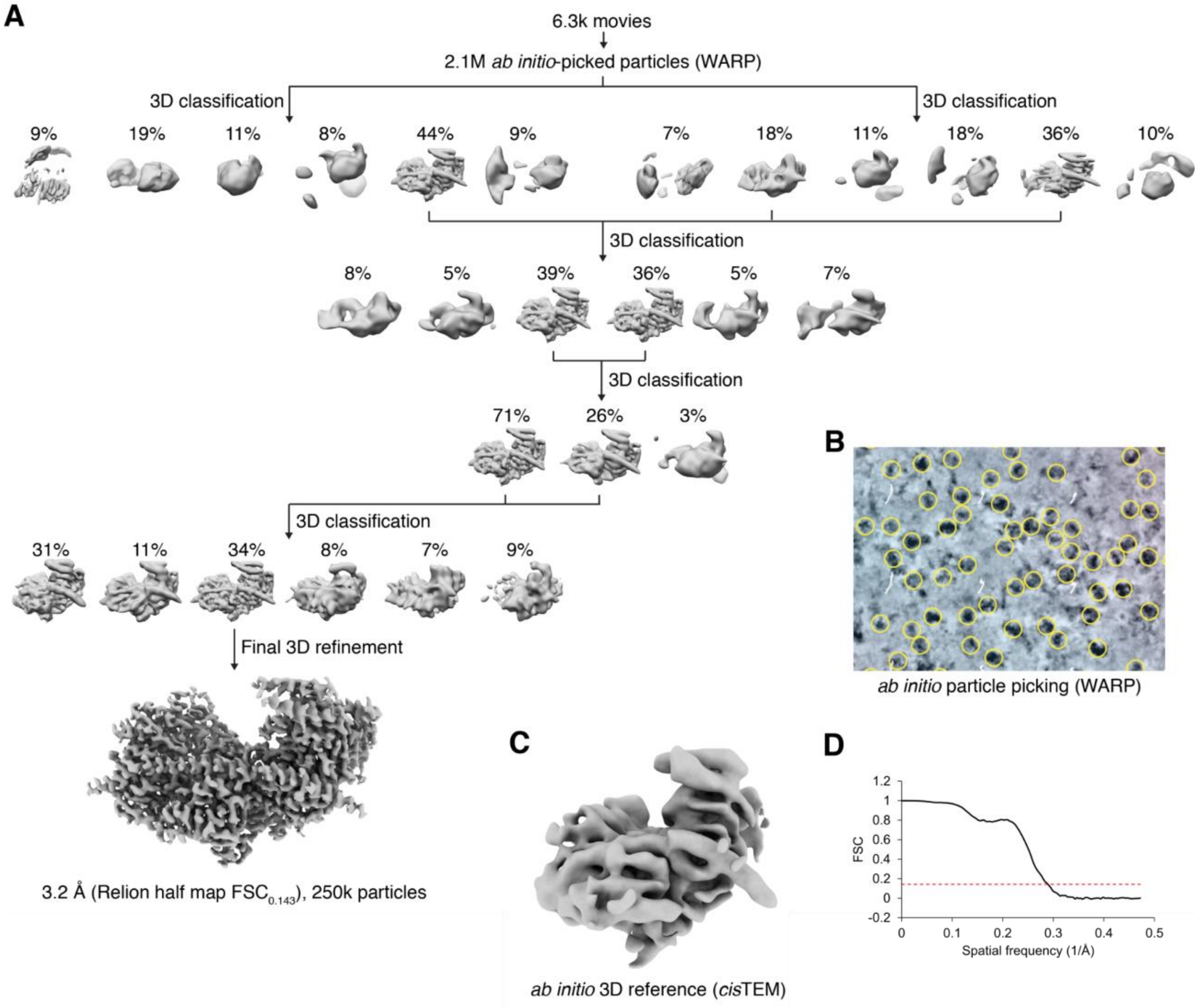

Fig. S12. Cryo-EM data analysis of *S. cerevisiae* A-module (ATPγS-state). (**A**) Good particles were selected through a series of focused 3D classifications and subsequently polished in Relion *(58)*. The final 3D reconstructions were generated and the resolution values calculated by Relion independent half map $FSC_{0.143}$ criterion. (**B**) The particles were picked *ab initio* and qualitatively filtered using WARP *(60)*. (**C**) The *ab initio* 3D model without DNA bound was generated in *cis*TEM *(61)* and used as the 3D reference for DNA-bound datasets to avoid bias in DNA presence and conformation. (**D**) Gold-standard Fourier shell correlation (FSC) curve of the final A-module reconstruction (no mask applied). The red line indicates the 0.143 cutoff criterion.

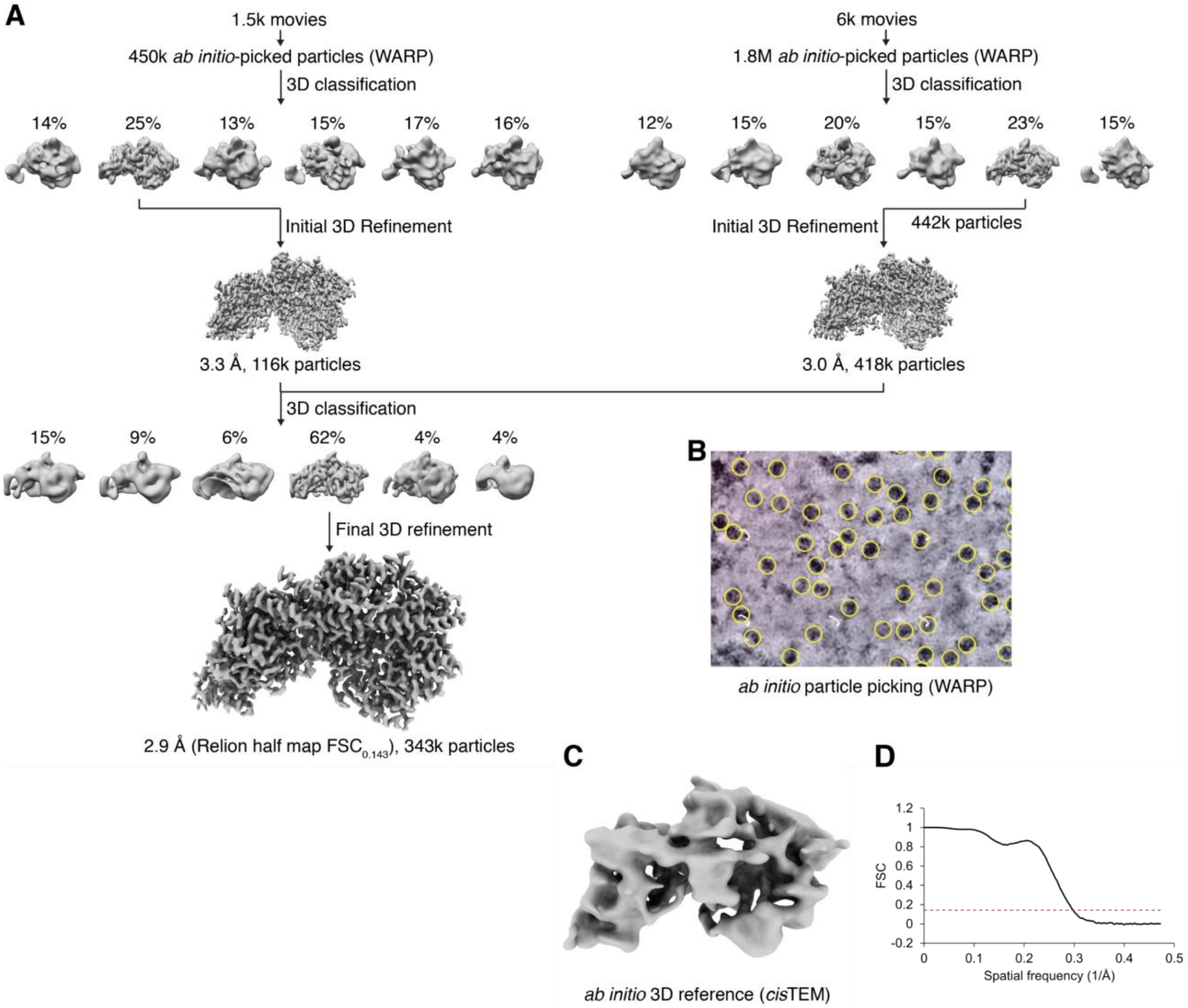

Fig. S13. Cryo-EM data analysis of *C. thermophilum* A-module (ATPγS-state). (**A**) Good particles were selected through a series of focused 3D classifications and subsequently polished in Relion *(58)*. The final 3D reconstructions were generated and the resolution values calculated by Relion independent half map $FSC_{0.143}$ criterion. (**B**) The particles were picked *ab initio* and qualitatively filtered using WARP *(60)*. (**C**) The *ab initio* 3D model without DNA bound was generated in *cis*TEM *(61)* and used as the 3D reference for DNA-bound datasets to avoid bias in DNA presence and conformation. (**D**) Gold-standard Fourier shell correlation (FSC) curve of the final A-module reconstruction (no mask applied). The red line indicates the 0.143 cutoff criterion.

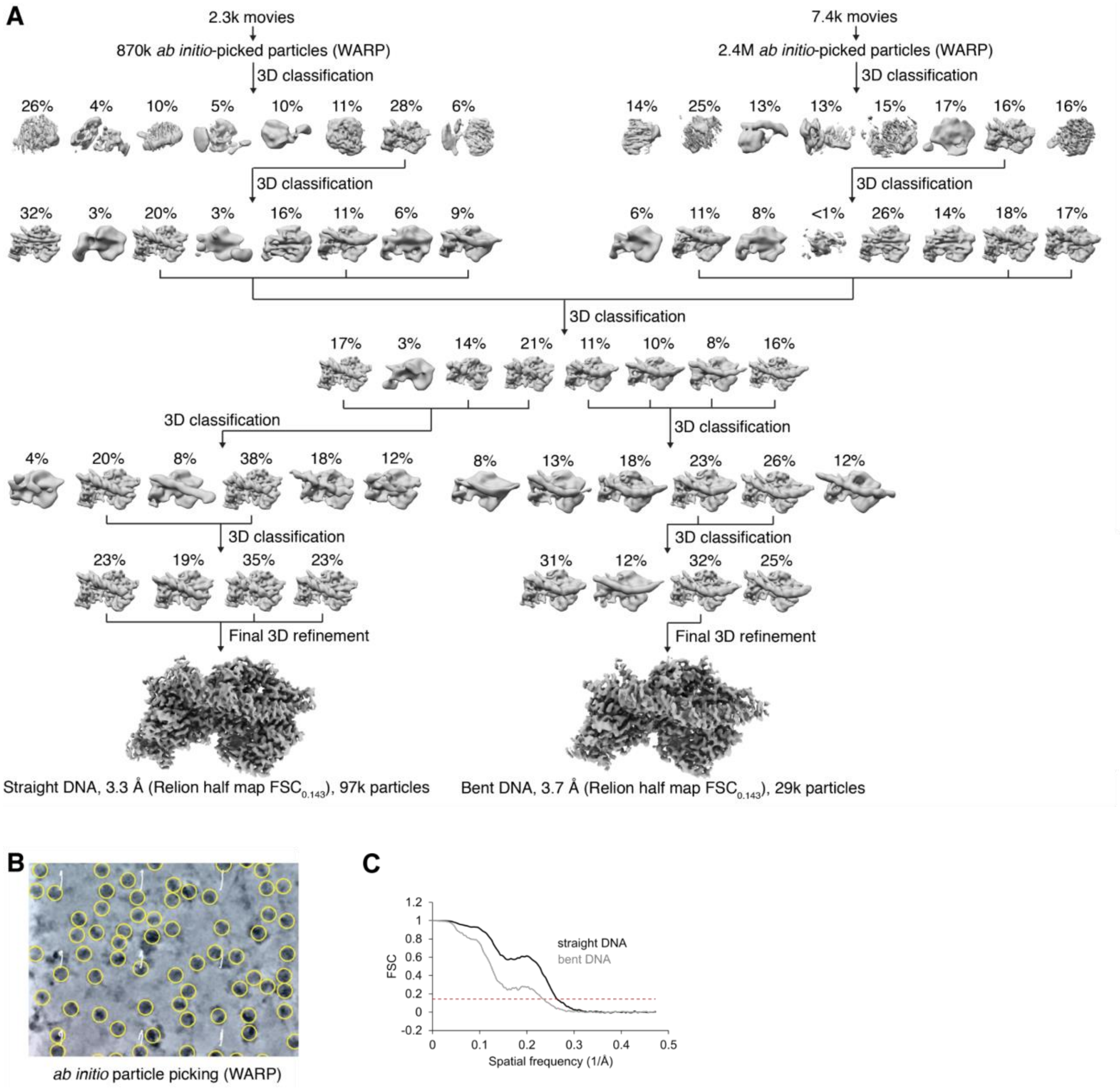

Fig. S14. Cryo-EM data analysis of *C. thermophilum* A-module and DNA (ADP·AlF_x). The *ab initio* 3D model without DNA bound was generated in *cis*TEM *(61)* and used to avoid bias in DNA presence and conformation. (**A**) DNA-bound A-module species were isolated from DNA-free species, followed by discernment and isolation of A-modules with straight and curved DNA bound through a series of focused 3D classifications in Relion *(58)*. The final 3D reconstructions were generated and the resolution values calculated by Relion independent half map $FSC_{0.143}$ criterion. (**B**) The particles were picked *ab initio* and qualitatively filtered using WARP *(60)*. (**C**) Gold-standard Fourier shell correlation (FSC) curves of the final A-module reconstructions (no mask applied). The red line indicates the 0.143 cutoff criterion.

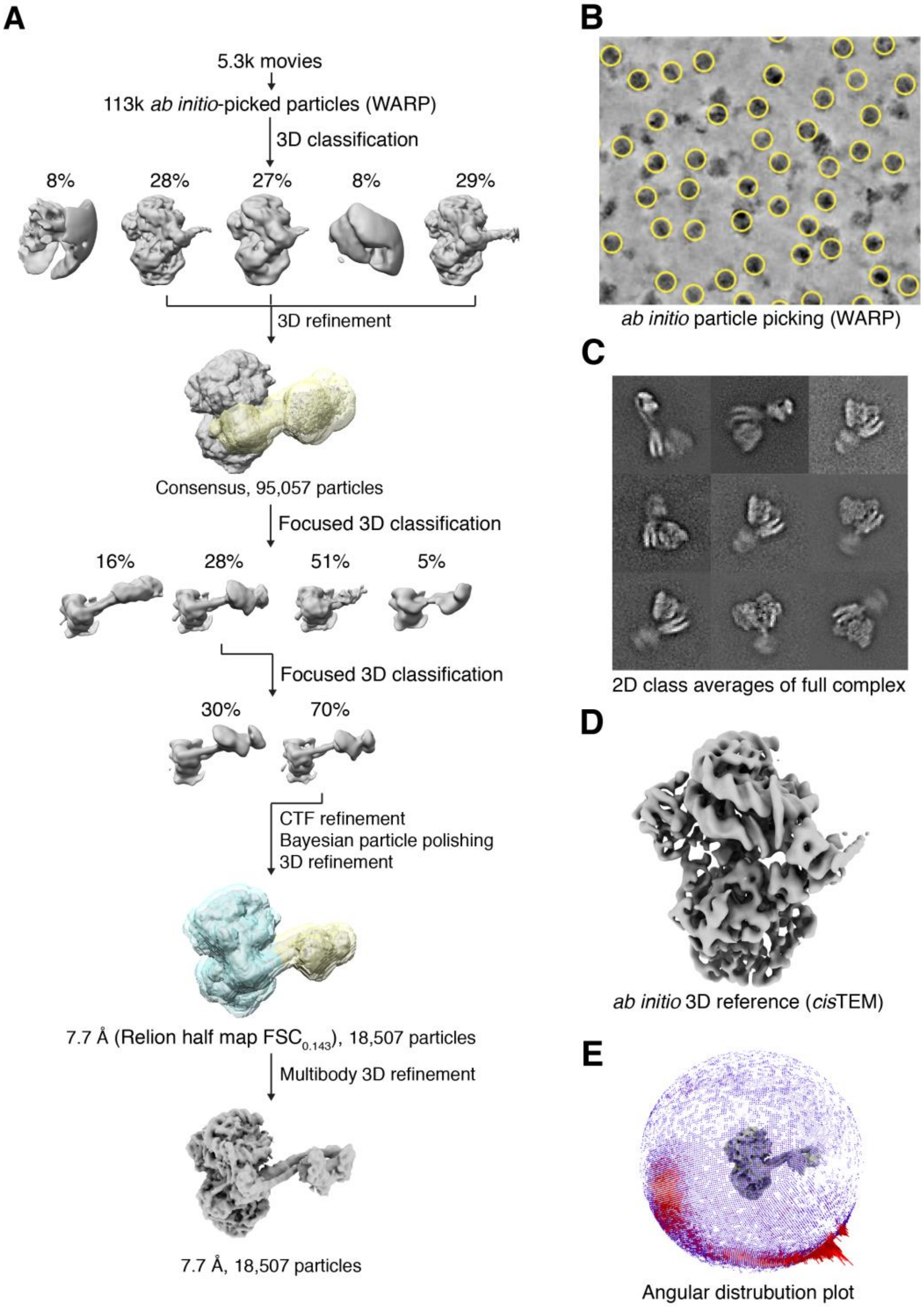

Fig. S15. Cryo-EM data analysis of *C. thermophilum* INO80$^{\Delta N}$. (**A**) A small subpopulation with the C- and A-modules more coherently placed were isolated through a series of focused 3D classifications in Relion and the final 3D reconstruction was generated by Relion multibody 3D refinement *(58)*. (**B**) The particles were picked *ab initio* and qualitatively filtered using WARP *(60)*. (**C**) 2D class averages of isolated full INO80 complex (**D**) The *ab initio* 3D model was generated in cisTEM *(61)*. (**E**) Angular distribution plot of the full INO80 complex 3D reconstruction.

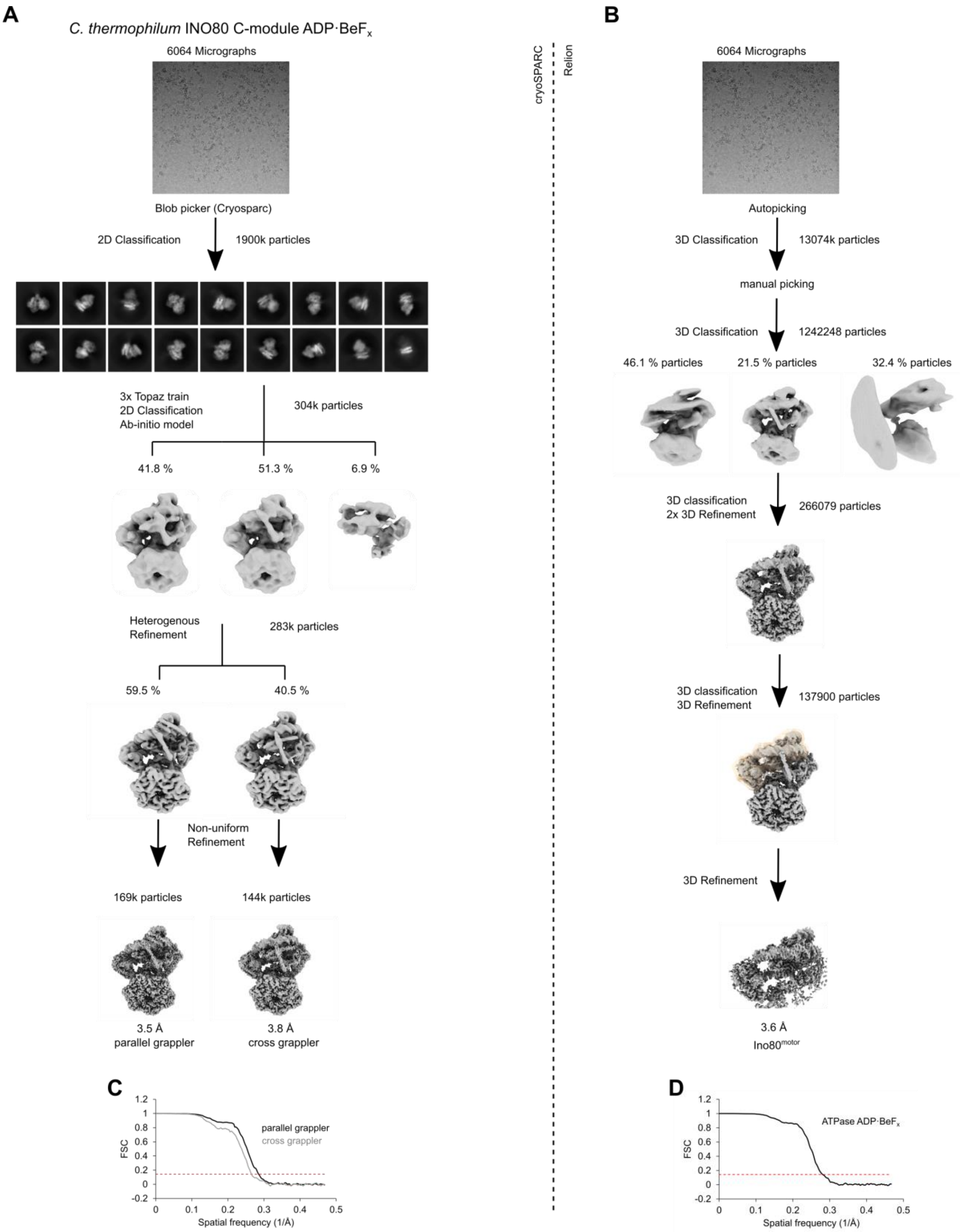

Fig. S16. Cryo-EM data analysis of *C. thermophilum* INO80 C-module with 0N80 nucleosomes. (**A**) Cryo-EM data processing workflow o*f C. thermophilum* INO80 C-module with

ADP·BeF_x using cryoSPARC v3.2.0 *(57)*. Representative micrograph of INO80 and representative classes of a 2D classification of the particles used for the final INO80 C-module reconstruction. (**B**) Local Refinement of the $Ino80^{motor}$ bound to nucleosome (**C-D**) Gold-standard Fourier shell correlation (FSC) curves of the final (**C**) INO80 C-module bound to the nucleosome with Arp5 grappler in parallel and cross conformation and (**D**) $Ino80^{motor}$ bound to the nucleosome (ADP·BeF_x). The red line indicates the 0.143 cutoff criterion.

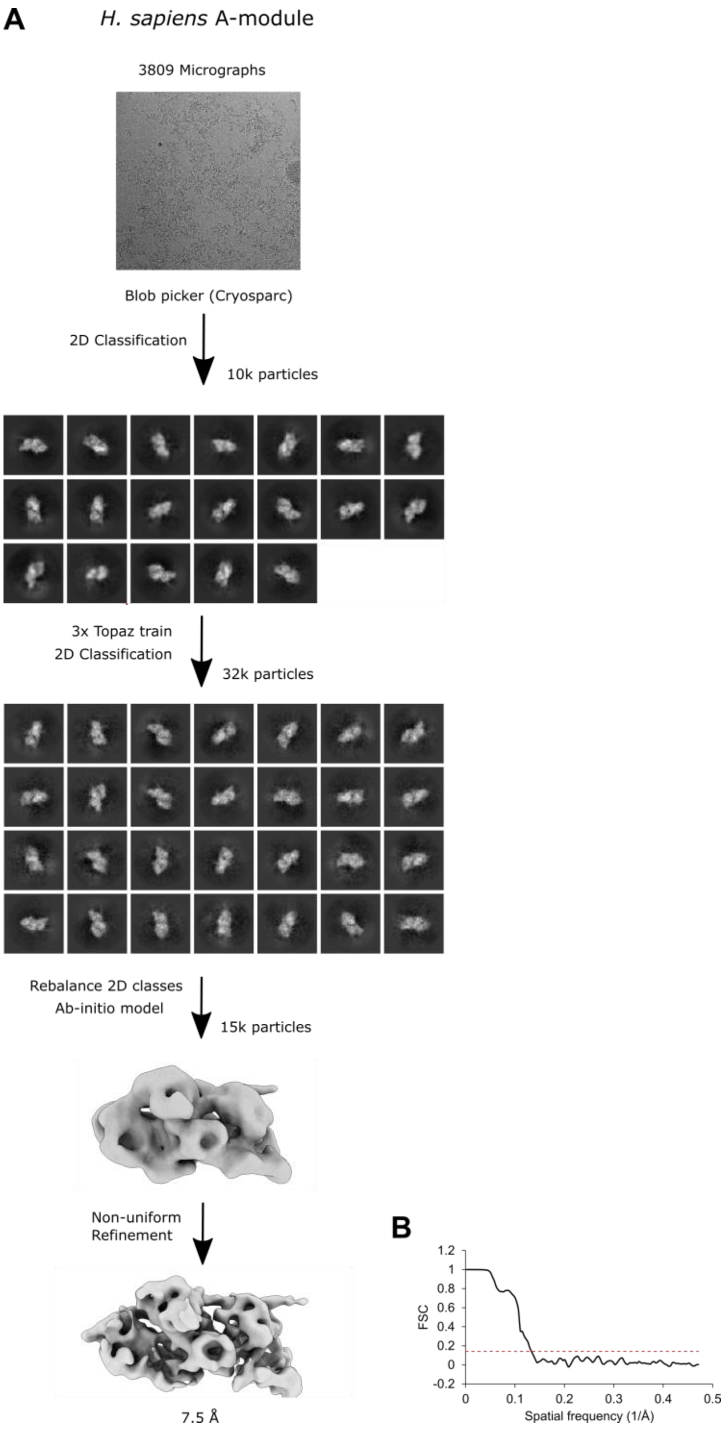

Fig. S17. Cryo-EM data analysis of *H. sapiens* A-module. (**A**) Cryo-EM data processing workflow of *Hs*A-module using cryoSPARC v3.2.0 *(57)*. Representative micrograph of *Hs*A-module and representative classes of a 2D classification of the particles used for the final A-module reconstruction after an *ab initio* model. (**B**) Gold-standard Fourier shell correlation (FSC) curve of the final A-module reconstruction. The red line indicates the 0.143 cutoff criterion.

Table S1. Cryo-EM data collection, refinement and validation statistics.

Complex	S.c A-module + ATPγS	S.c. A-module + ATP	S.c. A-module + ATP+ DNA	S.c. A-module + ADP	C.t. A-module + ATPγS	C.t. A-module + curved DNA (*apo*)	C.t. A-module + curved DNA (ADP-AlF_x)	C.t. A-module + straight DNA (ADP-AlF_x)	C.t. Ino80 C-module: cross grappler (ADP-BeF_x)	C.t. Ino80 C-module: parallel grappler (ADP-BeF_x)	C.t. Ino80motor + 0N80 nucleosome (ADP-BeF_x)	C.t. Ino80ΔN+0N80 nucleosome (*apo*)	h.s. A-module (*apo*)	C.t. Ino80 C-module (PDB: 6FML and EMDB 4277 and 4278)
**Data collection and Processing**														
Magnification	130	130	130	130	130	130	130	130	130	130	130	130	130	
Voltage (kV)	300	300	300	300	300	300	300	300	300	300	300	300	300	
Electron exposure (e-/Å^2)	47.37	44.68	44.68	45	44	50.5	42.4	42.4	48.4	48.4	48.4	50.5	52	
Defocus range (μm)	-1.1 to -2.9	-1.1 to -2.9	-1.1 to -2.9	-1.1 to -2.9	-1.1 to -2.9	-1.1 to -2.9	-1.1 to -2.9	-1.1 to -2.9	-1.1 to -2.9	-1.1 to -2.9	-1.1 to -2.9	-1.1 to -2.9	-1.1 to -2.9	
Pixel size (Å)	1059	1059	1059	1059	1059	1059	1059	1059	1059	1059	1059	1059	1059	
Symmetry imposed	C1	C1	C1	C1	C1	C1	C1	C1	C1	C1	C1	C1	C1	
Initial particle images (no.)	2100k	1230k	1230k	2300k	2250k	1180k	3240k	3240k	1900k	1900k	13074k	113k	2787k	
Final particle images (no.)	250k	327k	69k	970k	343k	325k	29k	97k	169k	144k	138k	18k	15k	
Map Resolution [Å]	3.2	3.3	7.5	3.2	2.9	3.4	3.7	3.3	3.8	3.5	3.6	7.7	7.5	
R-factor	0.29	0.27		0.27	0.27	0.28	0.29	0.28			0.24			
FSC	0.84	0.88		0.88	0.86	0.86	0.84	0.83			0.89			
**Refinement**														
Initial model used														
Model composition														
Non-hydrogen atoms	12571	13193			12225	13937		13537			15719			45030
Protein residues	1555	1629			1519	1550		1552			1219			4996
DNA nucleotides	–	–			–	72		52			286			290
Ligands (ATPγS, ATP, ADP)	3	3			3	3		3			1			8
Ions (Mg^{2+})	3	3			3	3		3			–			–
B-factors [Å^2]														
Protein	122.6	197.4			109.8	146.3		117.6			142.1			197.3
DNA	–	–			–	478.5		348.6			177.1			291.0
Ligands & Ions	73.7	138.6			77.5	98.7		74.2			145.1			129.8
RMS deviations (REFMAC5)														
Bond lengths [Å]	0.010	0.011			0.012	0.009		0.008			0.010			0.009
Bond angles [°]	1.71	1.88			1.75	1.82		1.72			2.31			1.78
Molprobity statistics/validation														
Molprobity Score	1.02	1.49			0.90	1.23		0.97			1.65			1.68
All-Atom Clashscore	1	2.44			0.86	2.33		1.13			4.15			4.15
Rotamer Outliers [%]	0.43	1.58			0.23	0.9		0.38			1.81			1.81
Ramachandran plot														
Favored [%]	96.5	95.47			97.27	96.61		97.2			96.23			96.23
Allowed [%]	3.31	4.22			2.60	3.26		2.74			3.1			3.1
Outliers [%]	0.19	0.31			0.13	0.13		0.07			0.67			0.67

Table S2. ***In vivo*** **yeast strains.**

name	gene	mutation
HSA^{Q1}	INO80	R482Q, K483Q, K487Q, R490Q, R504Q, K505Q, R512Q, K515Q, K516Q
HSA^{Q2}	INO80	K523Q, R533Q, R534Q, R537Q, K544Q, K545Q, R551Q, K554Q, K555Q
HSA^{Q1+Q2}	INO80	R482Q, K483Q, K487Q, R490Q, R504Q, K505Q, R512Q, K515Q, K516Q, K523Q, R533Q, R534Q, R537Q, K544Q, K545Q, R551Q, K554Q, K555Q
HSA^{A2}	INO80	K523A, K526A, R533A, R534A, R537A, K544A, K545A, R548A
$ino80^{E842A}$	INO80	E842A
arp8ΔN	ARP8	Delta of 1-197

Table S3. *In vivo* yeast mutants.

BP5148	*MATa, ade3::PGAL-HO, hmlΔ::pRS-1 hmrΔ::pRS-2, ChrIV491kb::GFPHOcs-hphNT1, ChrIV795kb::GFPHOinc-kanMX4, matHOcsΔ::pBR-1, URA3::YIplac211-empty*
BP5150	*MATa, ade3::PGAL-HO, hmlΔ::pRS-1 hmrΔ::pRS-2, ChrIV491kb::GFPHOcs-hphNT1, ChrIV795kb::GFPHOinc-kanMX4, matHOcsΔ::pBR-1, ino80::natNT2, URA3::INO80-2FLAG*
BP5155	*MATa, ade3::PGAL-HO, hmlΔ::pRS-1 hmrΔ::pRS-2, ChrIV491kb::GFPHOcs-hphNT1, ChrIV795kb::GFPHOinc-kanMX4, matHOcsΔ::pBR-1, ino80::natNT2, URA3::ino80-HSA-Q1-2FLAG*
BP5159	*MATa, ade3::PGAL-HO, hmlΔ::pRS-1 hmrΔ::pRS-2, ChrIV491kb::GFPHOcs-hphNT1, ChrIV795kb::GFPHOinc-kanMX4, matHOcsΔ::pBR-1, ino80::natNT2, URA3::ino80-HSA-Q2-2FLAG*
BP5415	*MATa, ade3::PGAL-HO, hmlΔ::pRS-1 hmrΔ::pRS-2, ChrIV491kb::GFPHOcs-hphNT1, ChrIV795kb::GFPHOinc-kanMX4, matHOcsΔ::pBR-1, ino80::natNT2, URA3::ino80-HSA-A2-2FLAG*
BP5137	*MATa/MATα, 2n[ade3::PGAL-HO, hmlΔ::pRS-1 hmrΔ::pRS-2, ChrIV491kb::GFPHOcs-hphNT1, ChrIV795kb::GFPHOinc-kanMX4], matHOcsΔ::pBR-1, ino80::natNT2, Δura3/URA3::ino80-HSA-Q1-2FLAG*
BP5138	*MATa/MATα, 2n[ade3::PGAL-HO, hmlΔ::pRS-1 hmrΔ::pRS-2, ChrIV491kb::GFPHOcs-hphNT1, ChrIV795kb::GFPHOinc-kanMX4], matHOcsΔ::pBR-1, ino80::natNT2, Δura3/URA3::ino80-HSA-Q2-2FLAG*
BP5139	*MATa/MATα, 2n[ade3::PGAL-HO, hmlΔ::pRS-1 hmrΔ::pRS-2, ChrIV491kb::GFPHOcs-hphNT1, ChrIV795kb::GFPHOinc-kanMX4], matHOcsΔ::pBR-1, ino80::natNT2, Δura3/URA3::ino80-HSA-Q1+Q2-2FLAG*
BP5140	*MATa/MATα, 2n[ade3::PGAL-HO, hmlΔ::pRS-1 hmrΔ::pRS-2, ChrIV491kb::GFPHOcs-hphNT1, ChrIV795kb::GFPHOinc-kanMX4], matHOcsΔ::pBR-1, ino80::natNT2, Δura3/URA3::ino80-E842A-2FLAG*
BP5128	*MATa, ade3::PGAL-HO, hmlΔ::pRS-1 hmrΔ::pRS-2, ChrIV491kb::GFPHOcs-hphNT1, ChrIV795kb::GFPHOinc-kanMX4, matHOcsΔ::pBR-1, arp8::natNT2, URA3::2FLAG-ARP8*
BP5125	*MATa, ade3::PGAL-HO, hmlΔ::pRS-1 hmrΔ::pRS-2, ChrIV491kb::GFPHOcs-hphNT1, ChrIV795kb::GFPHOinc-kanMX4, matHOcsΔ::pBR-1, ura3Δ, arp8::natNT2*
BP5133	*MATa, ade3::PGAL-HO, hmlΔ::pRS-1 hmrΔ::pRS-2, ChrIV491kb::GFPHOcs-hphNT1, ChrIV795kb::GFPHOinc-kanMX4, matHOcsΔ::pBR-1, arp8::natNT2, URA3::2FLAG-arp8-aa1-197Δ*

Table S4. Oligonucleotides (5’ to 3’) for *C. thermophilum* and *S. cerevisiae* cloning.

C. thermophilum	
Ino80-720-1855: open-vector-fwd	ATATTTATAGGTTTTTTTATTACAAAACTG
Ino80-720-1855: open-vector-rev	TCTAGAGCCTGCAGTCTCG
Ino80dN-rev	ACTGCAGGCTCTAGATTAATTAATCACTTGTCGTCATCA
Ino80dN- fwd	AGCTCAAGTTTCAGTCCAAAGGCTACAACC
Ino80-1-850:NA- fwd	CGCAAAGAAGAAGCCGACTACAAGGACGACGATGACAAG
Ino80-1-850NA-rev	CGTCGTCCTTGTAGTCGGCTTCTTCTTTGCGAGCATTC
Ino80-545-850A- fwd	ATGCAGCGTAAGCGGCGTCGCGAGAAGTCG
Ino80-545-850A-rev	ATTTATAGGTTTTTTTATTACAAAACTG
HSA1-insert- fwd	ACCAGATTTACGATCAGATCTGGCGTGATCTGGCTGCCG
HSA1-insert-rev	CGTTTCGCCCTAGCCTGGAGATCCTTTGTGCCCTTGTTGGTACGC
HSA1-vector- fwd	CTCCAGGCTAGGGCGAAACGTGTCATGCG
HSA1-vector-rev	GATCTGATCGTAAATCTGGTTGTAGCCTTTGGACTGAAACTTGAGCTCC
Insert HSA1	TGGCGTGATCTGGCTGCCGCGGATGTTAGCGCAGTTTTCGCTCTGGCGACGGACTCGTACGCTACCAAAGCATCCAACCTCAAGGCGACGGCCATCCTGGCTTCCGCGGAAGCAAAAGCCTGGCAACTGCGTACCAACAAGGGCACAAAGGAT
HSA2-insert- fwd	CTTCCAACCTCAAGAAGACGGCCATCCTGGCTTCCAAGGAAGC
HSA2-insert-rev	CGAGCATTCTCGAGTTCGAGCCTTTCAGCAGCCTTGCGTAAATCG
HSA2-vector- fwd	CTCGAACTCGAGAATGCTCGCAAAGAAGAAGC
HSA2-vector-rev	CGTCTTCTTGAGGTTGGAAGCTTTGG
Insert HSA2	GCCATCCTGGCTTCCAAGGAAGCCAAACGCTGGCAACTGCGTACCAACGCTGGCACAGCCGATCTCCAGGCTAGGGCGGCAGCGGTCATGGCTGACATGATGGGCTTCTGGGCCGCAAACGAGGCGGAAGAGCGCGATTTACGCAAGGCTGCTGAAAGG
Ino80.1- fwd	CCCCTGGAGCTCGCGTTTCAGTCCGCAGGCTACAACCAG
Ino80.1-rev	CTGGTTGTAGCCTGCGGACTGAAACGCGAGCTCCAGGGG
Ino80.2- fwd	GATCAGATCTGGGCTGATCTGGCTGCCAAAGATGTTAGC
Ino80.2-rev	GCTAACATCTTTGGCAGCCAGATCAGCCCAGATCTGATC
Arp5.1- fwd	TGCGGCAGGCGGCGCCGGCGCAGAC
Arp5.1-rev	GCTGCGGCGCTTCCCGAGCCGGTCGGGTTG
Arp5.2- fwd	GAAAGCGCAGCGGAAATCGCGGCTCTCCTCGACG
Arp5.2-rev	CGTCGAGGAGAGCCGCGATTTCCGCTGCGCTTTC
Arp8.1-vector-rev	CATATTTATAGGTTTTTTTATTACAAAACTGTTACGAAAACAGTAAAATACTTATTTATTTGCGAGATGG
Arp8.1-insert-fwd	TAAAAAAACCTATAAATATGGTGGGAAAAGTGAGCGAGGCCG
Arp8.1-vector- fwd	GATCAGATCCTGTCGCTGCGCCTCCAGAACGAGG
Arp8.1-insert-rev	CGCAGCGACAGGATCTGATCATCGCGGGCCATATAATCAGTATAGGCG
Insert Arp8.1	GTGGGAAAAGTGAGCGAGGCCGTGCTTGCCGCCGAAGGGCTGGAACGCACCGACAATGGCATGAGGCAAACAAGCTGGCCTGAGGTGCAGCCCATTGCCGCAGCTGCCGCATATACTGATTATATGGCCCGCGAT
Arp8dN-rev	CATATTTATAGGTTTTTTTATTACAAAACTGTTACGAAAACAG
Arp8dN- fwd	CTGATTAACCAAAAGAACTATTATACTGATTATATGAAGCGCG
S. cerevisiae	
Ino80-598-fwd	GTATTCGCATTTCATTGGAAGGAAAGACTACAAGGACGACGATGACAAG
Ino80-598-rev	CGTCCTTGTAGTCTTTCCTTCCAATGAAATGCGAATACAATTC

Table S5. Oligonucleotides (5’ to 3’) for nucleosome preparation, anisotropy and cryo-EM.

Nucleosome-0N80-fwd	CTGGAGAATCCCGGTGCCGAGG
Nucleosome-0N80-rev	CGGTACCCGGGGATCCTCTAG
**DNA templates (5´FAM)**	
601 sequence	CTGGAGAATCCCGGTGCCGAGGCCGCTCAATTGGTCGT AGCAAGCTCTAGCACCGCTTAAACGCACGTACGCGCTG TCCCCCGCGTTTTAACCGCCAAGGGGATTACTCCCTAGT CTCCAGGCACGTGTCAGATATATACATCCTGTGCATGT ATTGAACAGCGACCTTGCCGGTGCCAGTCGGATAGTGT TCCGAGCTCCCACTCTAGAGGATCCCCGGGTACCGA
601 sequence SHL -4/5	CTGGAGAATCCCGGTGCCGAGGCCGCTCAATTGGTCGT AGCAAGCTCTAGCACCGCTTAAACGCACGTACGCGCTG TCCCCCGCGTTTTAACCGCCAAGGGGATTACTCCCTAGT TCTTTTTTTTTTTTCAGATATATACATCCTGTGCATGTAT TGAACAGCGACCTTGCCGGTGCCAGTCGGATAGTGTTC CGAGCTCCCACTCTAGAGGATCCCCGGGTACCGA
601 sequence SHL -6/7	CTGGAGAATCCCGGTGCCGAGGCCGCTCAATTGGTCGT AGCAAGCTCTAGCACCGCTTAAACGCACGTACGCGCTG TCCCCCGCGTTTTAACCGCCAAGGGGATTACTCCCTAGT CTCCAGGCACGTGTCAGATCTTTTTTTTTTTTGCATGTA TTGAACAGCGACCTTGCCGGTGCCAGTCGGATAGTGTT CCGAGCTCCCACTCTAGAGGATCCCCGGGTACCGA
601 sequence SHL -8/9	CTGGAGAATCCCGGTGCCGAGGCCGCTCAATTGGTCGT AGCAAGCTCTAGCACCGCTTAAACGCACGTACGCGCTG TCCCCCGCGTTTTAACCGCCAAGGGGATTACTCCCTAGT CTCCAGGCACGTGTCAGATATATACATCCTGTGCATGT ATTGATCTTTTTTTTTTTTGGTGCCAGTCGGATAGTGTTC CGAGCTCCCACTCTAGAGGATCCCCGGGTACCGA
601 sequence SHL -10/11	CTGGAGAATCCCGGTGCCGAGGCCGCTCAATTGGTCGT AGCAAGCTCTAGCACCGCTTAAACGCACGTACGCGCTG TCCCCCGCGTTTTAACCGCCAAGGGGATTACTCCCTAGT CTCCAGGCACGTGTCAGATATATACATCCTGTGCATGT ATTGAACAGCGACCTTGCCGGTGCTCTTTTTTTTTTTTTC CGAGCTCCCACTCTAGAGGATCCCCGGGTACCGA
**Anisotropy DNA**	
Aniso_fwd (5´FAM)	AATTGGTCGTAGCAAGCTCTAGCACCGCTTAAACGCAC GTACGCGCTGTC
Aniso_rev	GACAGCGCGTACGTGCGTTTAAGCGGTGCTAGAGCTTG CTACGACCAATT
**cryo-EM DNA**	
EM_fwd	CTTACCCTGCGTGCCCGCGCACCCTGGCGACTTCGCCTC GTTTTGGCGATTTTCTTAG
EM_rev	CTAAGAAAATCGCCAAAACGAGGCGAAGTCGCCAGGG TGCGCGGGCACGCAGGGTAAG

Supplementary Movie 1 legend

Movie 1 illustrates the conformational change of the DNA bound to the *Ct*A-module with different interaction sites (3D variability analysis in cryoSPARC).

Supplementary Movie 2 legend

Structural morph illustrating the conformational changes of the $Ino80^{motor}$ bound to the nucleosome in the apo-state or bound to $ADP{\cdot}BeF_x$.

Supplementary Movie 3 legend

Movie 3 illustrates the conformational change of the Arp5 grappler and its contacts to the entry DNA of the nucleosome (3D variability analysis in cryoSPARC).

Raw data file legend

Excel file including raw data for Fig. 2D, Fig. 4G to I, Fig. 7D and E, Fig. 8C and D, Fig. S4D, Fig. S5, Fig. S6B, Fig. S9D.
